# Fast Release of Carboxylic Acid inside Cells

**DOI:** 10.1002/cmdc.202500056

**Published:** 2025-02-17

**Authors:** Pascal Moser, Renaud Zelli, Leandro J. dos Santos, Mickaël Henry, Kevin Sanchez‐Garcia, Yvan Caspar, Florian C. Marro, Benoit Chovelon, Jaione Saez Cabodevilla, Sandrine Ollagnier de Choudens, Eric Faudry, Yung‐Sing Wong

**Affiliations:** ^1^ Univ. Grenoble Alpes CNRS DPM 38000 Grenoble France; ^2^ Univ. Federal de Viҫosa UFV-CAF 35690000 Florestal Brasil; ^3^ Laboratoire de Bactériologie-Hygiène Hospitalière CHU Grenoble Alpes CS10317 38043 Grenoble cedex 9 France; ^4^ Univ. Grenoble Alpes CNRS CEA IBS Bact.Path. & Cel. Resp. 38000 Grenoble France; ^5^ Biozentrum University of Basel Basel 4056 Switzerland; ^6^ Univ. Grenoble Alpes CEA CNRS LCBM, UMR 5249 38000 Grenoble France

## Abstract

Delivering carboxylic acid functions into cells is challenging due to their poor permeability across lipophilic membranes at physiological pH, where they are ionized. Masking carboxylic acids as esters improves cell entry, but once inside the cell, its rapid release is essential to maintain spatiotemporal control which can be beneficial for therapeutic and diagnostic applications. This study evaluates the 2‐hydroxyethyl‐dithio‐benzyl ester functional group which undergoes selective and rapid cleavage of the disulfide bond by thioredoxin (Trx), triggering rapid self‐immolation of the thio‐benzyl ester releasing the carboxylic acid. Fluorescence‐based assays using the pro‐fluorescent BODIPY structure have demonstrated the rapid intracellular release of carboxylic acids within minutes in both eukaryotic and prokaryotic cells. The approach was tested on antibiotics, and among them, levofloxacin ester prodrug, having the 2‐hydroxyethyl‐dithio‐benzyl ester functional group, showed significantly enhanced antimicrobial activity against resistant and intracellular bacteria compared to its methyl ester analogue.

## Graphical abstract

Masking carboxylic acids as esters enhances cellular entry, but rapid intracellular release is crucial to improve efficacy. 2‐Hydroxyethyl‐dithio‐benzyl, rapidly cleaved by intracellular thioredoxin, followed by fast carboxylic acid release by self‐immolation, is designed with a fluorogenic carboxylic acid BODIPY. A levofloxacin prodrug containing this ester, compared with a simple ester, exhibits enhanced antimicrobial activity against resistant and intracellular bacteria.

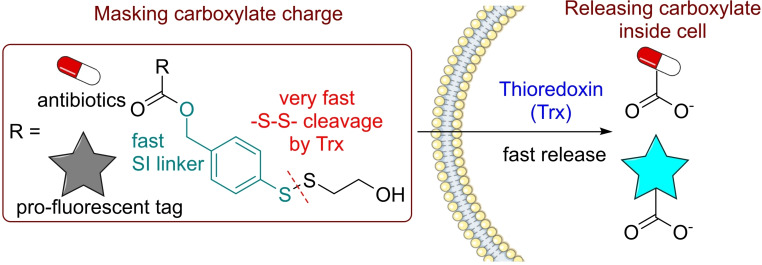

## Introduction

The presence of a carboxylic acid function, essential for the activity of a drug or a molecular probe can be a real challenge when it is necessary to cross cell membranes or hydrophobic physiological barriers. At physiological pH, this group is ionized, carrying a negative charge, which impedes or retards its passive diffusion through lipophilic membranes. To facilitate or accelerate entry into cells, the carboxylic acid group must be masked in the form of a neutral function, usually an ester. In addition, the speed at which the carboxylic acid is released once inside the cell plays a crucial role in determining the spatio‐temporal precision of its action. This precision is especially important for applications requiring a rapid localized increase in concentration inside cell, such as enhancing the sensitivity of bioreporters or boosting the biological activity of drugs.

**Scheme 1:**
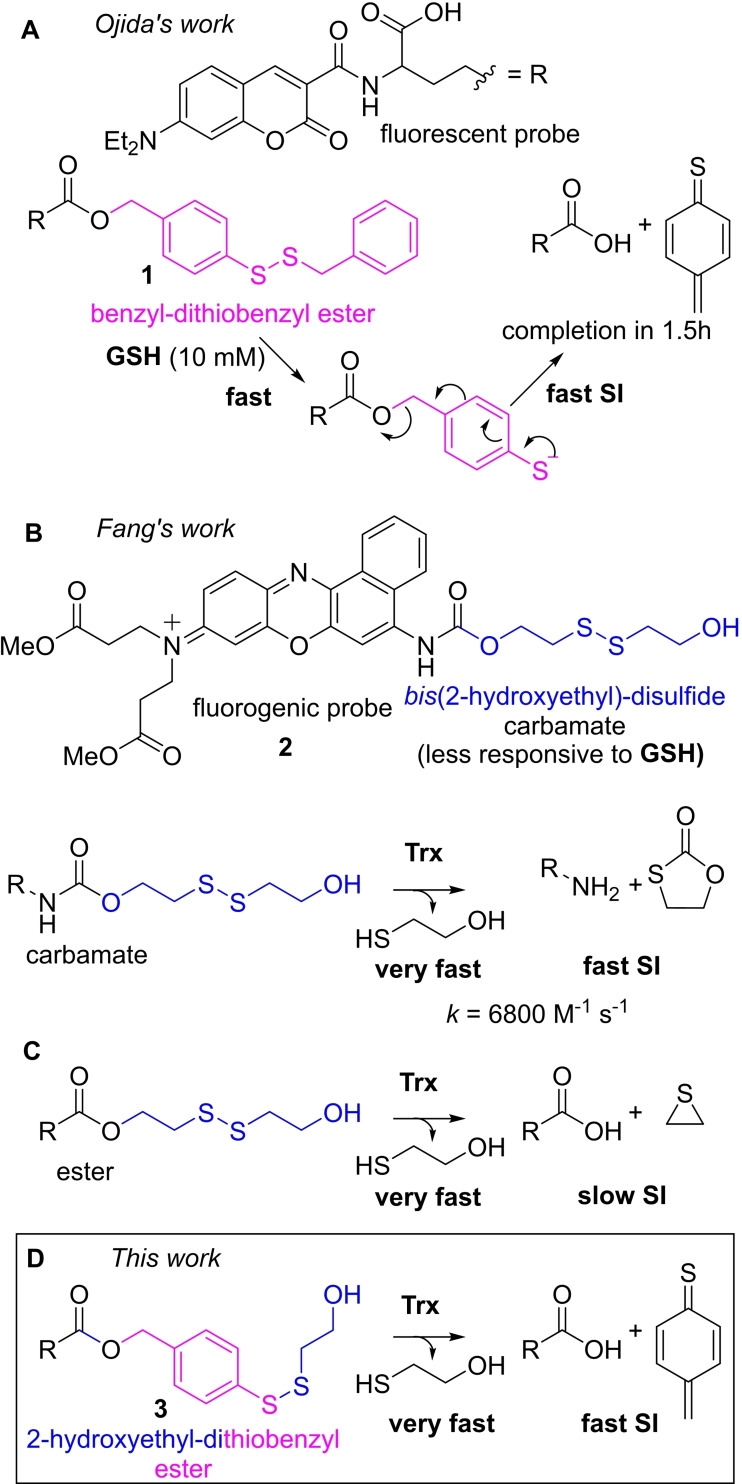
**A**: Ojida's glutathione (GSH)‐responsive fluorescent probe **1** with the benzyl‐dithiobenzyl ester functional group for carboxylic acid delivery. **B**: Fluorogenic Nile blue carbamate thioredoxin (Trx) sensor **2** described by Fand and coworkers with the rapid cleavage of the −S−S− bond by Trx followed by a fast self‐immolation (SI) release of aniline through rapid formation of the five‐membered thiocarbonate ring. **C**: Extension to the ester group may lead to slow carboxylic acid release due to slow formation of the three‐membered thio‐epoxide ring. **D**: Fast and selective self‐immolative ester **3** combining very fast cleavage of the −S−S− bond by Trx and rapid SI release of carboxylic acid.

To achieve precise and rapid release of a carboxylic acid from an ester, three main strategies have been explored, namely, physico‐, chemo‐ and bio‐stimulations. Among physical triggers, light has gained attention due to advancements in photoremovable ester protecting groups, such as coumarins and BODIPYs, which are cleavable in demand under biocompatible visible light (up to 538 nm and 689 nm, respectively).^[1]^ However, *in vitro* applications on cells remain sparse,^[2]^ and *in vivo* application is hampered by tissue penetration issues. Chemical triggers, such as bioorthogonal cleavage of tetrazine‐caged esters activated by *trans*‐cyclooctene, provide another alternative.[^3^, ^4^] However, the slow penetration of the triggering agent into cells (often requiring hours of pre‐incubation) compromises spatio‐temporal control. On the other hand, bio‐stimulation by endogenous agents localized within the cell offers a simpler and more effective approach. Certain ester forms of carboxylic acids can be selectively recognized, especially by the O‐alkylated part, and then rapidly cleaved by specific intracellular esterases.[^5^, ^6^, ^7^] However, their presence inside cells is not guaranteed in all cell types. Hemiacetal esters have been employed as functional group irreversibly cleavable by enzymes, which can accelerate the release process. Notable examples include their use as prodrug groups for β‐lactams targeting bacterial periplasm, such as Pivampicilin and Bacampicilin, or in the context of the tumor‐target drug delivery using nanoparticules.^[8]^ Other bio‐stimuli to unlock carboxylic acid from esters with more specificity for intracellular media have been reported, particularly in bacteria, such as the reductions of nitro‐benzyl group by nitro reductases followed by self‐immolation (SI) of the resulting amino‐benzyl group[^9^, ^10^] or the cleavage of the disulfide bond by high intracellular concentration of glutathione (GSH).^[11]^ However, detailed data on the release rates of these mechanisms remain scarce. Ojida and colleagues^[12]^ conducted pioneering work on the controlled release of carboxylic acid function from a benzyl‐dithiobenzyl ester group. This system was designed to undergo cleavage through the reduction of disulfide bonds by GSH, followed by rapid release of carboxylic acid by the self‐immolation (SI) of the thiobenzyl ester group (Scheme 1A). Compound **1**, carrying the fluorescent coumarine probe, was used to study its cleavage *in vitro* and its internalisation *in cellulo*. The high concentration of glutathione inside the cell was expected to make release rapid. Although the fluorescence was visible inside the eucaryotic cell, the ester and acid forms have the same fluorescence, ruling out the possibility of tracking carboxylic acid release inside the cell. More recently, Fang and co‐workers identified that thioredoxin (Trx), an essential redox‐regulating protein located within cell, can selectively cleave *bis*(2‐hydroxyethyl) disulfide bond in eukaryotic cell to unlock aniline function from a carbamate group (Scheme 1B).^[13]^ Interestingly, this reaction, coupled with a fast ring‐forming SI mechanism driven by the favourable formation of the five‐membered thiocarbonate ring,^[14]^ proceeds at a remarkably high rate (*k*=6800 M^−1^ s^−1^). In addition, the uses of the pro‐fluorescent Nile Blue probe, which emits fluorescence upon the release of aniline function, enabled the effective monitoring of its intracellular release.

Inspired by this result, we wonder if this rapid *bis*(2‐hydroxyethyl) disulfide bond cleavage by Trx can be adapted for the fast release of carboxylic acid. However, in the case of an ester, the direct grafting of the *bis*(2‐hydroxyethyl) disulfide bond to the ester group means that the release mechanism would require the formation of a more strained three‐membered thio‐epoxy ring, which might delay the release (Scheme 1C). In the case of carbamates designed to release amines, the speed of this SI cyclization can be increased by introducing substituents on the ring,^[15]^ or by using the self‐immolative dithio‐benzyl function group to accelerate this releasing step.[^16^, ^17^] In parallel, Thorn‐Seshold and co‐workers recently introduced a bicyclic disulfide structure, selectively cleaved by Trx to release phenol functionalized probes.^[18]^ Building on the works of Ojida and Fang, we combined their approaches to design a novel ester group that serves as a fast‐releasing carboxylic acid linker specific to intracellular environments (Scheme 1D). The linear 2‐hydroxyethyl‐dithio‐benzyl ester **3** was designed to be selectively recognized and very rapidly cleaved by Trx, triggering a subsequent fast self‐immolative process to release carboxylic acid within the cell. This strategy for the intracellular rapid release of carboxylic acids was evaluated using antibiotics, including a β‐lactam, a quinolone, and a fluoroquinolone. In the context of prodrug application, the success lies in its ability to release the active compound selectively once it reaches its destination, i. e. in the case of our antibiotics, in the periplasm (β‐lactam) or cytoplasm (fluoroquinolone and quinolone) of the bacteria. However, these antibiotics in native form may benefit from a natural internalization pathway, such as via porins.[^19^, ^20^] It would be interesting to see whether our approach designed for enhanced passive diffusion and rapid intracellular release could compete with the natural internalization of antibiotics. Additionally, Kwong and co‐workers have demonstrated that the effectiveness of prodrug antibiotics relies on their ability to achieve a sufficiently high intracellular concentration to inhibit bacterial growth.^[21]^ Prodrugs may fail if bacterial growth outpaces the rate of prodrug activation. To address this, the new antibiotics carrying the rapidly cleaving ester function were compared to their simple ester counterparts to evaluate their potential advantages.

## Results and Discussion

**Figure 1:**
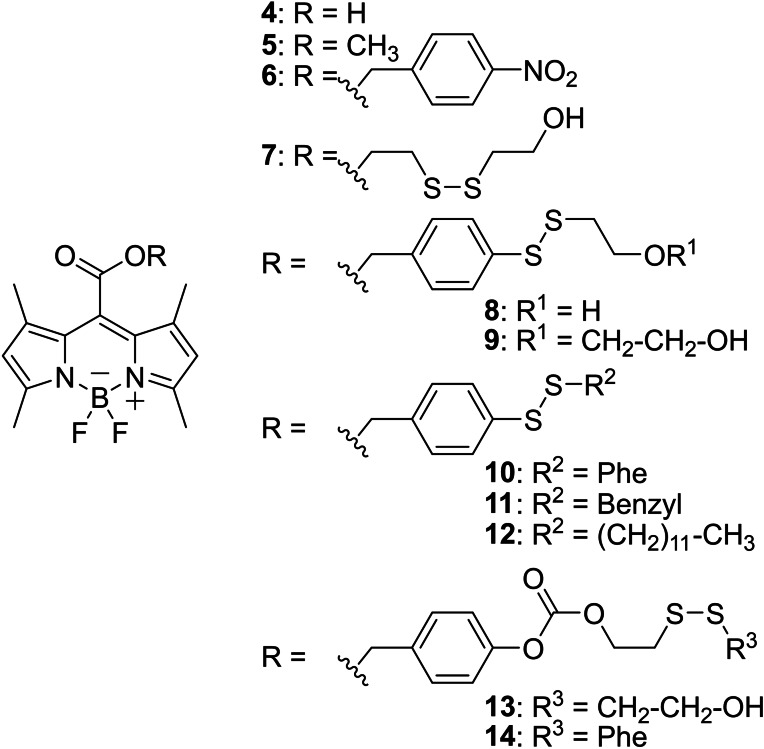
Fluorescent carboxylic acid BODIPY **4** and corresponding pro‐fluorescent esters **5**–**14**.

For this study, we developed a probe capable of detecting the cleavage inside prokaryotic and eukaryotic cells. A strong fluorescent chromophore was therefore needed to detect the release of carboxylic acid, as bacteria are much smaller than eukaryotic cells. We selected BODIPY carboxylic acid **4** (Figure 1) as a fluorogenic probe to be able to monitor its release inside the cell. Moreover, the presence of methyl groups close to the ester function protects it from esterase action.^[22]^

Methyl ester **5**,^[23]^ *p*‐nitro‐benzyl ester **6** and *bis*(2‐hydroxyethyl) disulfide ester **7** were synthesized to compare the action of esterases, nitro reductases and Trx on its original motif, respectively. For the subsequent compounds **8**–**12**, the thiobenzyl ester moiety was retained as a fast carboxylic acid SI releasing motif, implying that the speed of the overall carboxylic acid release process would be dependent on the rate of disulfide bridge cleavage. Esters **8** and **9** feature the studied 2‐hydroxyethyl‐dithio‐benzyl group, with the addition of a further 2‐hydroxyethyl motif in the case of **9**. Derivatives bearing a phenyl **10**, benzyl **11** and alkyl **12** group have also been synthesized. Finally, benzyl carbonates bearing either the *bis*(2‐hydroxyethyl) disulfide motif **13** or the 2‐hydroxyethyl‐dithio‐phenyl motif **14**, were obtained, involving, after cleavage of the −S−S− bond, two successive SI mechanisms to release the acid.

The reductive cleavage of self‐immolative disulfide or dithio‐benzyl bonds has been described to be dependent on glutathione (GSH) concentration.[^12^, ^24^] The significant difference in GSH concentration in serum (1–10 μM) and in the cytosolic compartment (1–10 mM) enables the selective cleavage of these bonds inside the cell. First, the behaviour of our compounds was tested at 1 μM for 1 h in the presence of extra‐ and intracellular GSH concentrations (1 μM and 1 mM, respectively) at the same concentrations reported by Fang and coworkers.^[13]^ They were also placed for 1 h in the presence of dithiothreitol (DTT), a non‐physiological dithiol and a stronger reducing agent that can mimic Trx behaviour to some extent. In GSH at 1 μM, all the compounds were stable or underwent a very slow release, such as compound **13** (Figure S1, ESI†). At 1 mM, releases were still slow, with no plateau reached after 1 h. For DTT, at both 1 μM and 1 mM concentrations, the appearance of a higher rise in fluorescence intensity indicates an increase in the rate of the carboxylic acid dye release, but again no total cleavage of compound was observed after 1 h Figure S2, ESI†). All these results indicate that the cleavage of our products was slow in the presence of GSH and are resistant to DTT at low concentration. Interestingly, we observed that the ratio between the carboxylic acid release rate and concentration of reducing agent (GSH or DTT) varied among the products. This difference may be explained from the fact that carboxylic acid release is governed by multiple and consecutive reactions, each with distinct kinetics. For instance, compound **13** was the only compound that exhibits some reactivity at low GSH concentrations (Figure S1A, ESI†), but remains at roughly the same release rate at higher GSH concentrations (Figure S1B, ESI†). This behaviour could be attributed to the involvement of three consecutive reactions in the release process, where one of the two SI reactions may proceed at a slower rate.

**Figure 2:**
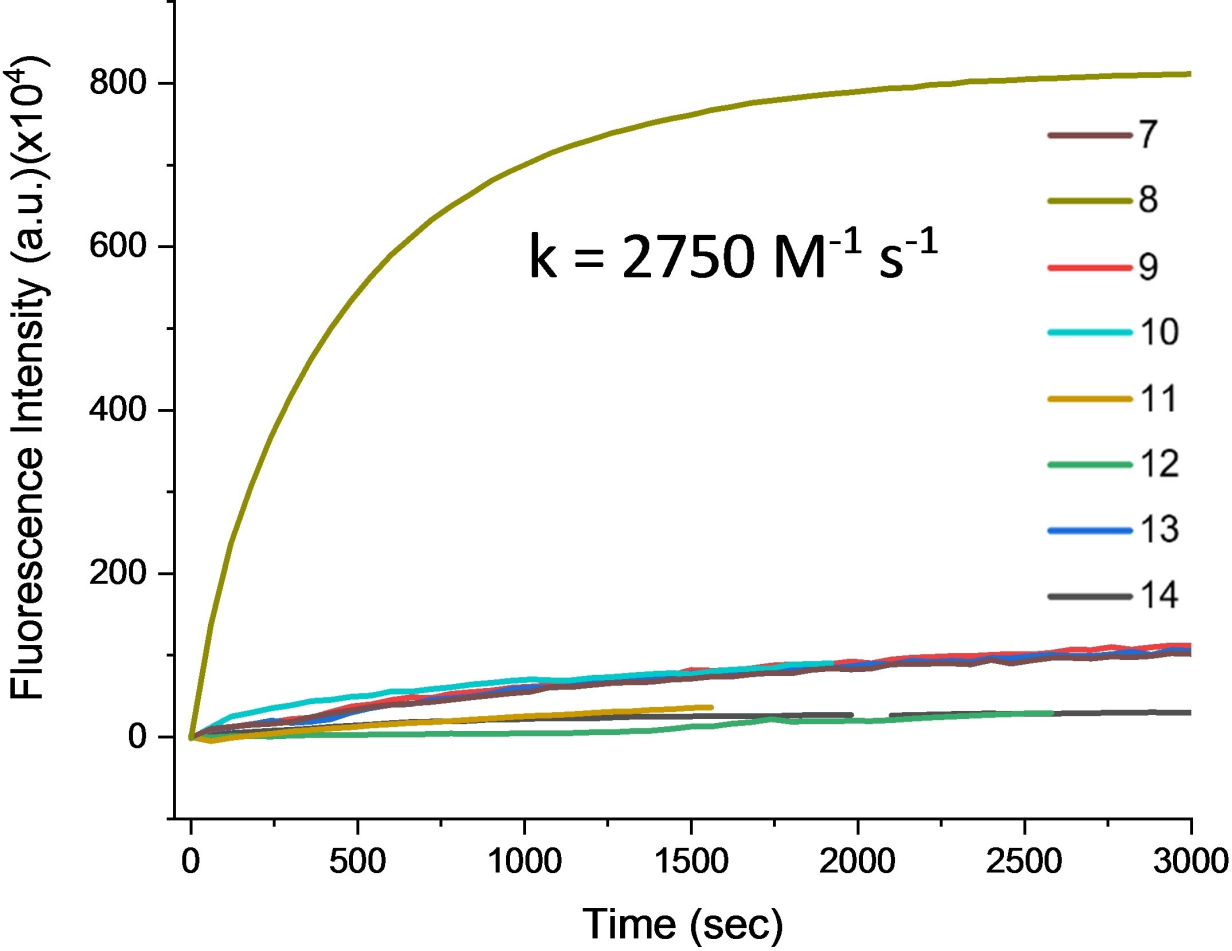
Reaction monitoring of the fluorogenic probes (1 μm in phosphate buffer (50 mm) w/ 1 % DMSO, pH=7.4) with Trx (658 nm) at 25 °C.

The compounds **7**–**14** containing an −S−S− functional group were then tested in the presence of reduced recombinant *Escherichia coli* Trx (Figure 2). We were delighted to observe that, as expected, compound **8** exhibited the fastest release of the carboxylic acid fluorescent dye **4**, triggered by Trx, reaching a plateau within just 25 min. In contrast, all other compounds demonstrated significantly slower release rate, likely due to either poor recognition by TrX or incomplete release of dye **4** by SI reaction following cleavage of the −S−S− bond. These results indicate that only compound **8** undergoes successive rapid reactions. The release kinetic of **4** from **8** was evaluated at *k*=2750 M^−1^ s^−1^ (Figure S3 and Table S1, ESI†).

To provide additional elements to this rapid mechanism after the cleavage of −S−S− bond, an HPLC/MS study of the release of fluorescent carboxylic acid **4** from compound **8** was carried out at a higher concentration (100 μM), to be able to detect intermediates, in the presence of DTT (200 mM, Figure S4, ESI†). The gradual disappearance of **8** to give compound **4** was visible without detecting the presence of other BODIPY intermediates between the two products. This suggests that once the −S−S− bond has been broken by Trx, cleavage of the self‐immolative dithio‐benzyl function occurs instantaneously in a traceless manner.

**Figure 3:**
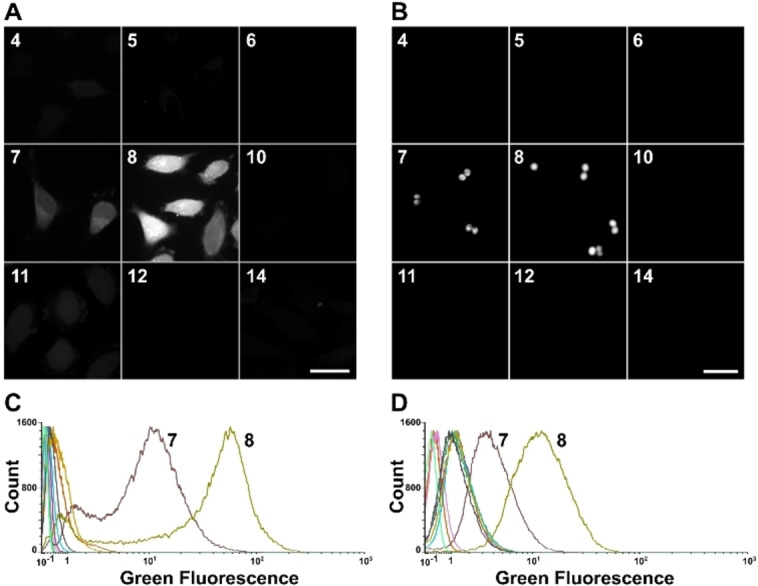
Microscopy images and fluorescence quantification of HeLa and S. aureus cells incubated with 9 different compounds. Eukaryotic and bacteria cells incubated with compounds **4**–**8**, **10**–**12** and **14** incubated for 10 to 15 min at 100 μM in culture medium. Confocal microscopy images of (**A**) HeLa (scale bar: 40 μm) and (**B**) *S. aureus* (scale bar: 10 μm) cells and cytometry distributions of fluorescence intensities of (**C**) HeLa and (**D**) *S. aureus* cells. Median Fluorescence Intensities determined for compounds **7** and **8** are 6.7 and 45.4, respectively for HeLa cells and 3.1 and 10.3, respectively for *S. aureus*.

To obtain visible labelling on cells, and especially on small bacterial cells, a higher concentration of compound was required. The toxicity of all the molecules was evaluated on human HeLa cells at 20 μM to exclude highly toxic molecules. The compounds **9** and **13** were found to be toxic at this concentration (Figure S5, ESI†), which ruled them out for further cell assays. To determine the ability of the remaining molecules to be internalized and rapidly release **4**, the assays were performed on HeLa cells and on the Gram‐positive *Staphylococcus aureus* bacterial cells by incubating our compounds (100 μM) with these cells at 37 °C, for 10 min (HeLa) and 15 min (*S. aureus*). No toxicity was observed under this condition for *S. aureus* with these molecules (Figure S6, ESI†). Fluorescence microscopy on both cell types showed that the compound releasing the fluorescent carboxylic acid probe **4** most rapidly in the cell was indeed compound **8** (Figure 3A and 3B). Compound **7** also proved effective, albeit producing less intense labelling than compound **8**, as shown by the cytometry results (Figure 3C and D), with three times less intensity in the case of *S. aureus* (Figure 3D). The labelling of **8** was homogeneous and cytoplasmic as shown in confocal images (Figure S9, ESI†). It is interesting to note that most of the tested ester functions, namely **4**–**5** and **10**–**12**, or the one carrying the *p*‐nitro‐benzyl function **6** did not undergo detectable cleavage by their respective enzymes, in a short time scale. In particular, the Ojida's linker **11** seems not to be cleaved quickly enough to be detectable under these conditions. The fact that compound **7** showed some efficiency in releasing fluorescent compound **4** into HeLa and *S. aureus* cells over a short period of time may seem surprising considering that in *in vitro* testing on Trx, cleavage of **7** took place very slowly (Figure 2). Alternatively, certain esterases could also rapidly cleave the ester bond, releasing carboxylic acid. This cleavage may occur because the enzymes recognize either the *bis*(2‐hydroxyethyl) disulfide ester motif, or its reduced form, the 2‐mercaptoethyl ester.

The β‐lactam penicillin G **15 a**, the fluoroquinolone levofloxacin **16 a** and the quinolone nalidixic acid **17 a** were next chosen as model antibiotics. Their respective 2‐hydroxyethyl‐dithio‐benzyl ester analogues **15 b**, **16 b** and **17 b**, as well as their methyl ester version **15 c**, **16 c** and **17 c** have been synthesized (Table 1). A preliminary screen was undergone to identify the most potent compound and to see if this new linker can be widely applied as a good prodrug functional group. Antimicrobial activity of these compounds was first screened *in vitro* to establish their minimum inhibitory concentration (MIC) against different laboratory reference strains for antibiotic susceptibility assays of *S. aureus*, *Bacillus subtilis*, for Gram‐positive bacteria, and *E. coli* and *Pseudomonas aeruginosa* for Gram‐negative bacteria (Table 1).

**Table 1:** MIC values of antibiotic **15 a**, **16 a** and **17 a** and their esterified analogues **15 b, c**, **16 b, c** and **17 b, c**.

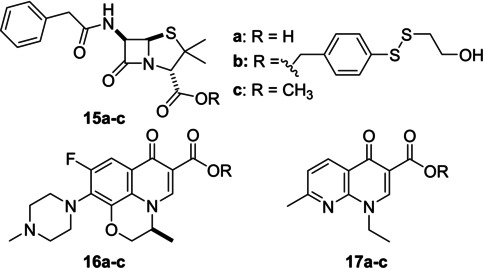				
	MIC (μg/mL)			
	Gram‐positive		Gram‐negative	
drug/ prodrug	*S. aureus* ATC29213	*B. subtilis* ATCC6633	*E. coli* ATCC25922	*P. aeruginosa* ATCC27853
**15 a**	1	0.25	32	>128
**15 b**	8	2	>128	>128
**15 c**	4	8	>128	>128
**16 a**	0.25	0.06	0.03	2
**16 b**	2	2	0.12	32
**16 c**	8	4	1	64
**17 a**	64	8	2	>128
**17 b**	>128	64	>128	>128
**17 c**	>128	>128	>128	>128

Among the compounds tested, only compound **16 b** demonstrated significant superiority in inhibiting *E. coli* compared to its simpler methyl analogue **16 c**. The MIC of **16 b** was approximately 10 times lower than that of **16 c**, or about 15 times lower when compared by molarity. In the case of β‐lactam penicillin G, the absence of any significant beneficial effect with **15 b** compared to its methyl ester **15 c** with all strains tested may be consistent with the fact that penicillin G **15 a** is ineffective on Gram‐negative bacteria and in the case of Gram‐positive bacteria, the target is outside the bacterium and therefore not subject to Trx action. The poor inhibitions obtained with **17 b,c** could reflect their difficulties in cell penetration. Noteworthy, none of 2‐hydroxyethyl‐dithio‐benzyl ester prodrug has demonstrated the ability to outperform the parent compound, likely because native antibiotics can rely on pores specifically dedicated to transport charged molecules, which seems to be more efficient than the passive diffusion of our uncharged ester analogues. Alternatively, more efficient efflux pumps may also be available for pumping out more hydrophobic (uncharged) compounds.

Compounds in the levofloxacin **16 a**–**c** series were further tested on drug resistant mutant strains. An improvement in inhibition for **16 b** compared with the methylated analog **16 c** was observed in all cases, confirming that the 2‐hydroxyethyl‐dithio‐benzyl group acts as a better prodrug function for levofloxacin compared to the methylated prodrug (Table 2). Interestingly, *S. aureus* SA‐1199B is known to overexpress the NorA efflux pump, which could explain its lower sensitivity to the action of **16 a**–**c** compared to ATCC 29213. Fluoroquinolones provide a relevant model here, as they affect intracellular bacterial targets. Their β‐ketoacid moiety is essential for antibiotic activity, inhibiting bacterial DNA gyrase and type IV topoisomerase.^[25]^

**Table 2:** MIC (μg/mL) of compounds **16 a**–**c** with different clinical and mutant strains.

	tested range (mg/L)	*S. aureus* ATCC 29213	*S. aureus* SA‐1199B	*S. aureus* ATCC 106414	*S. aureus* ATCC BAA44	*S. aureus* ATCC 700698	*S. aureus* ATCC 700699	*E. coli* CHUGA‐2018‐Eco1	*E. coli* CHUGA‐2018‐Eco2	*E. coli* CHUGA‐2018‐Eco10	*E. coli* CHUGA‐2018‐Eco24	*E. coli* ATCC 25922
**16 a**	0,25–128	≤0,25	1	16	4	16	≤0,25	32	32	2	1	≤0,25
**16 b**	0,25–128	2	4	64	16	64	1	>128	128	16	2	≤0,25
**16 c**	0,25–128	8	64	>128	>128	>128	8	>128	>128	64	32	1

**Figure 4:**
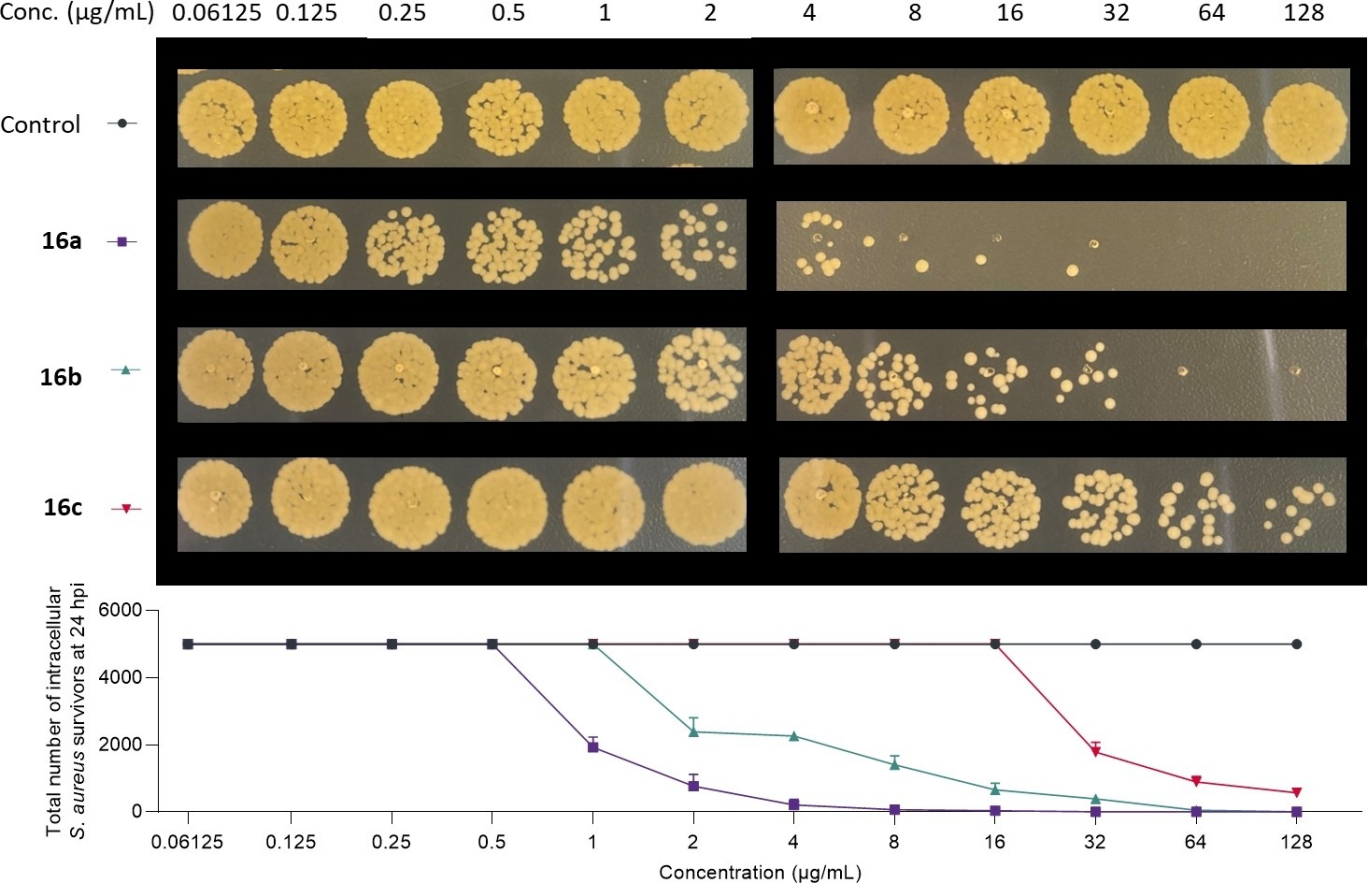
Ability of compounds **16 a**–**c** to kill intracellular *S. aureus* in A549 cells. Colony numbers were enumerated per spot.

Finally, the ability of molecules **16 a**–**c** (Figure 4) to kill intracellular bacteria^[26]^ was also evaluated and assessed on epithelial cells infected with *S. aureus*. Compounds **15 a**–**c** and **17 a**–**c** were also tested as control (Figure S10, ESI†). Interestingly, compound **16 b** again proved to be more effective than its methylated analogue **16 c** (Figure 4) with IMBC of 64 μg/mL and >128 μg/mL, respectively. However, once again, the native compound remained the most effective.

## Conclusions

This study introduces a method for the rapid incorporation of a carboxylic acid inside cells, by masking it with a 2‐hydroxyethyl‐dithio‐benzyl ester function that facilitates its cellular permeation and allowing a rapid and selectively cleavage once inside the cell through the action of intracellular Trx. This function, which is rapidly cleavable in all cells, offers new prospects in terms of intracellular biomarker applications and new therapeutic applications. Its enhanced passive cell penetration and its rapid cleavage properties have been used herein to improve the action of certain esterified antibiotic prodrug to kill both resistant bacteria and those localized in host cells. Compound **16 b** has been identified as being much more effective than the parent simple ester **16 c** and deserves further investigations, for instance, in its evaluation as prodrug. However, the native antibiotic **16 a** still outperformed their esterified prodrug analogues, highlighting the need to improve the kinetics of carboxylic acid release while maintaining good stability in biological media. This novel ability to rapidly bring molecules with carboxylic acid functions of interest into cells will certainly find other therapeutic applications or pave the way for developing new molecular tools.

## Conflict of Interests

The authors declare no competing interests.

1

## Supporting information

As a service to our authors and readers, this journal provides supporting information supplied by the authors. Such materials are peer reviewed and may be re‐organized for online delivery, but are not copy‐edited or typeset. Technical support issues arising from supporting information (other than missing files) should be addressed to the authors.

Supporting Information

## Data Availability

Data are available on request from the authors.
