# Supplementary material for: Fast Release of Carboxylic Acid inside Cells
Source: ChemMedChem. 2025 Feb 17;20(9):e202500056. doi: 10.1002/cmdc.202500056 (PMC12058233; doi:10.1002/cmdc.202500056)
Supplement: Supplementary file 1 — Supporting Information [file CMDC-20-e202500056-s001.pdf]

ChemMedChem

Supporting Information

Fast Release of Carboxylic Acid inside Cells

Pascal Moser, Renaud Zelli, Leandro J. dos Santos, Mickaël Henry, Kevin Sanchez-Garcia, Yvan Caspar, Florian C. Marro, Benoit Chovelon, Jaione Saez Cabodevilla, Sandrine Ollagnier de Choudens, Eric Faudry,* and Yung-Sing Wong*

Supporting Informations

Table des matières

1. Materials and methods with supplementary figures, schemes and tables S2

1.1. Fluorescence measurements S2

1.2. HPLC/MS analysis of BODIPY 2-hydroxyethyl-dithio-benzyl ester reduced by large amount of DTT S5

1.3. Cellular experiments S6

1.4. Condition to determine the Minimum Inhibitory Concentrations (MICs) S10

1.5. Eukaryotic cell infection and compounds evaluation S10

2. Synthesis and characterization of molecules 6 to 17b S12

3. NMR spectra S23

4. References S42

1 Materials and methods with supplementary figures, schemes and tables

1.1. Fluorescence measurements

Method: Fluorescence measurements were performed on a FluoroMax®-4, HORIBA Scientific, localized in DPM (UGA), or on a Jasco FP-8500 Fluorescence Spectrometer equipped with an FCT-816 Water thermostatted 4-position cell changer, localized in LCBM (CEA). Excitation was set at 474 nm (slit = 2 nm), and emission was collected at 535 nm (slit = 10 nm). Fluorescence intensity was measured every minute for one hour using Hellma® fluorescence cuvettes (ultra-micro cell, optical path length 3×3 mm, chamber volume 70 µL, Hellma GmbH, Germany). Fluorescence spectra were recorded on a Horiba Fluoromax-4P (DPM, Grenoble) or a Jasco FP-8500 Fluorescence Spectrometer equipped with an FCT-816 Water thermostatted 4-position cell changer (CEA, LCBM, Grenoble).

A

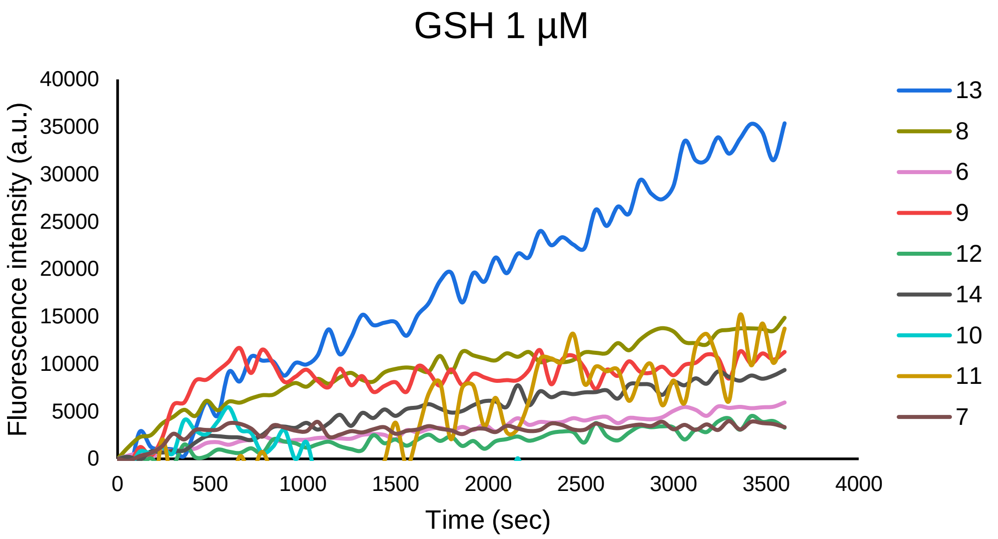

B

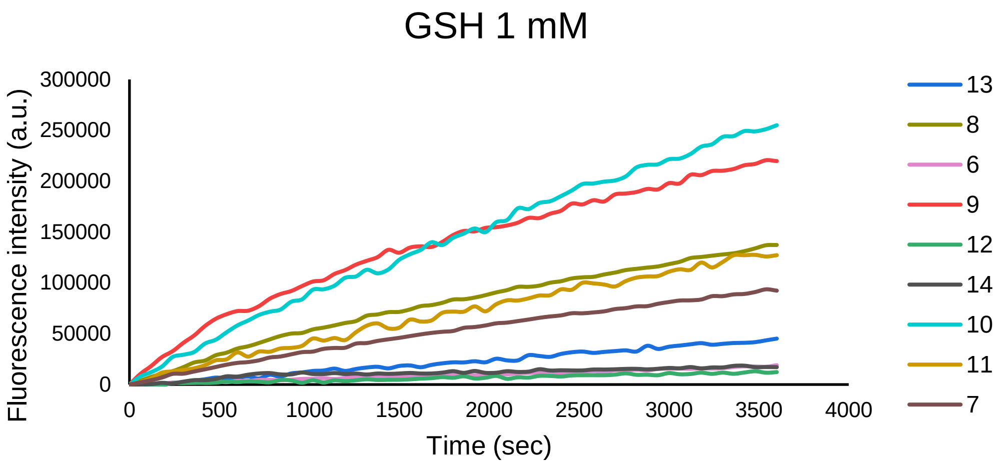

Fig. 1S Fluorescence intensity evolution of compounds **7-13** (1 µM) in phosphate buffer (50 mM w/ 1% DMSO, pH = 7) with **A**: 1 µM GSH; **B**: 1 mM GSH at 25°C. Measurements done at $\lambda_{excitation}$ = 474 nm and $\lambda_{emission}$ = 535 nm (Horiba Fluoromax-4P).

A

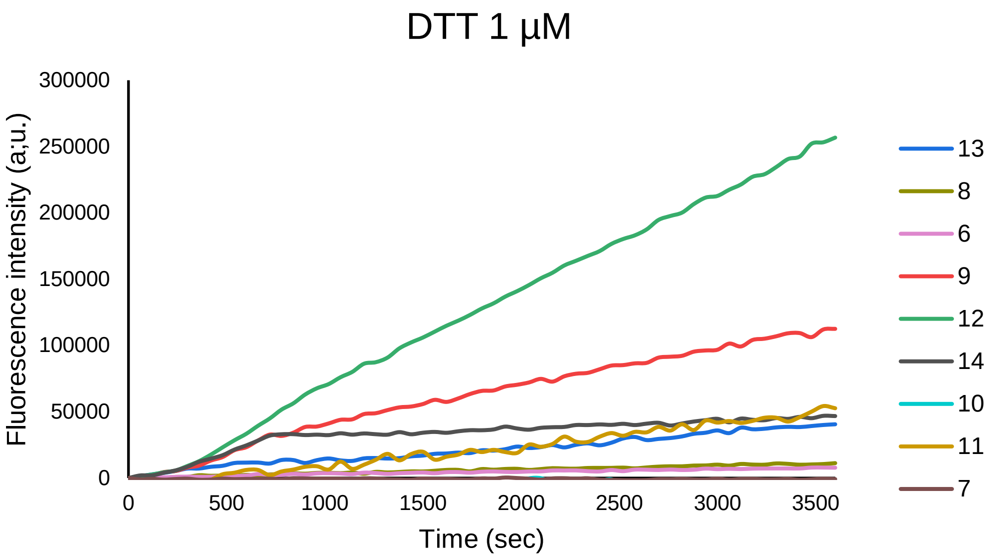

B

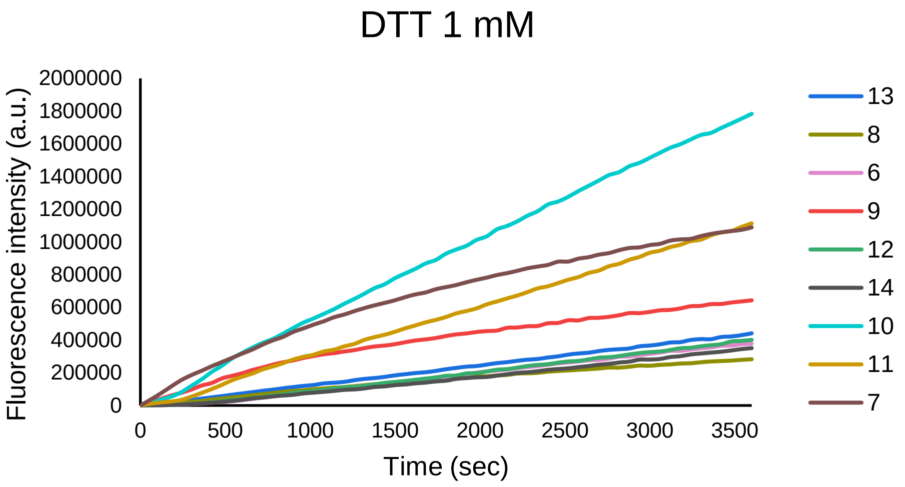

Fig. 2S Fluorescence intensity evolution of compounds **7-13** (1 µM) in phosphate buffer (50 mM w/ 1% DMSO, pH = 7) with **A**: 1 µM DTT; **B**: 1 mM DTT at 25°C. Measurements done at $\lambda_{excitation}$ = 474 nm and $\lambda_{emission}$ = 535 nm (Horiba Fluoromax-4P).

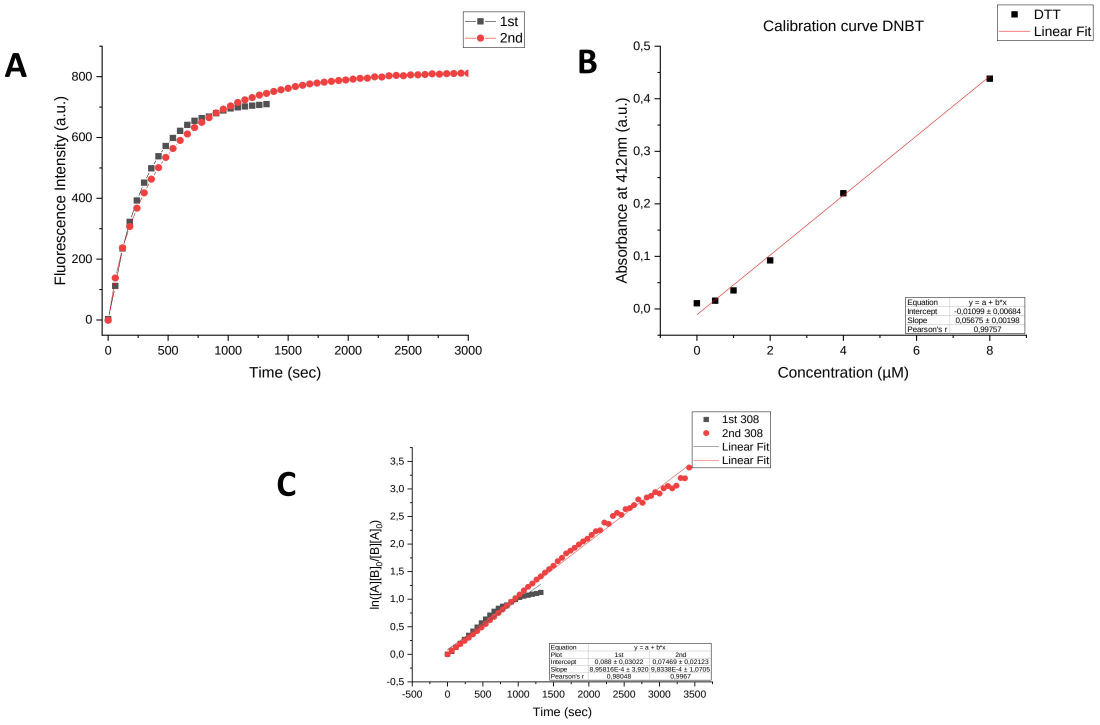

Fig. 3S Determination of the rate constant of compound **8** with recombinantTrx[1] from two independent measurements (Jasco FP-8500). **A**: Representative reaction monitoring of **8** (1 µM in phosphate buffer (50 mM) w/ 1% DMSO, pH = 7.4) with Trx (658 nM) . **B**: Calibration curve for the absorbance of 2-nitro-5-thiobenzoic acid (TNB) at 412 nm after reduction of Ellman's reagent (DTNB) with known concentrations of dithiothreitol (DTT) for 15 min, obtained from three independent measurements. **C**: Plot of $\ln([A][B]_0/[B][A]_0)$ *vs* time of **8** (1.0 µM) with Trx (658 nM). In $\ln([A][B]_0/[B][A]_0)$, [A] and [B] are $[A]_0 - (F/Fmax)[A]_0$ and $[B]_0 - (F/Fmax)[A]_0$, respectively, at given time. The second order rate constant (*k*) for reaction of probe **8** with Trx was obtained from repeat experiments (n = 2). The concentration of the reduced Trx was determined by titration with DTNB and measuring the absorption of TNB at 412 nm (n=3), reading from the previously recorded calibration curve (**B**) then gives the real concentration of reduced Trx. All kinetic experiments were performed in phosphate buffer (50 mM, pH = 7.4) at 25 °C.

Table 1S The second-order rate constant of **8** with Trx from two independent experiments.

Experiment	R [a]	$[A]_0$ [b]	$[B]_0$ [c]	$k_{EcTRX}([A]_0-[B]_0)$	k_{EcTRX} [d]
1st	0.9805	1.0×10^{-6}	6.58×10^{-7}	8.96×10^{-4}	2.62×10^{3}
2nd	0.9967	1.0×10^{-6}	6.58×10^{-7}	9.83×10^{-4}	2.88×10^{3}
				Average of k	**2.75×10^{3}**
				SD	1.28×10^{2}

[a] The linear correlation in the plots of ln([A][B]0/[B][A]0) vs time. [b] The initial concentration of **8** in mol/L. [c] The initial concentration of Trx in mol/L. [d] The slope of the plot which is the second-order rate constant k [$M^{-1}s^{-1}$].

1.2. HPLC/MS analysis of BODIPY 2-hydroxyethyl-dithio-benzyl ester reduced by large amount of DTT

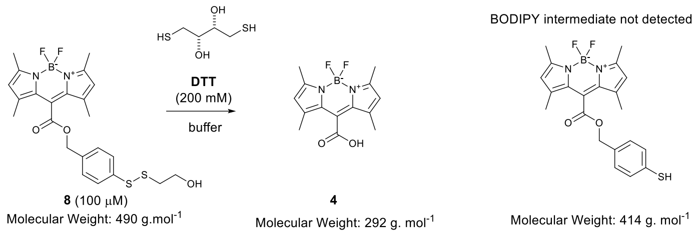

A

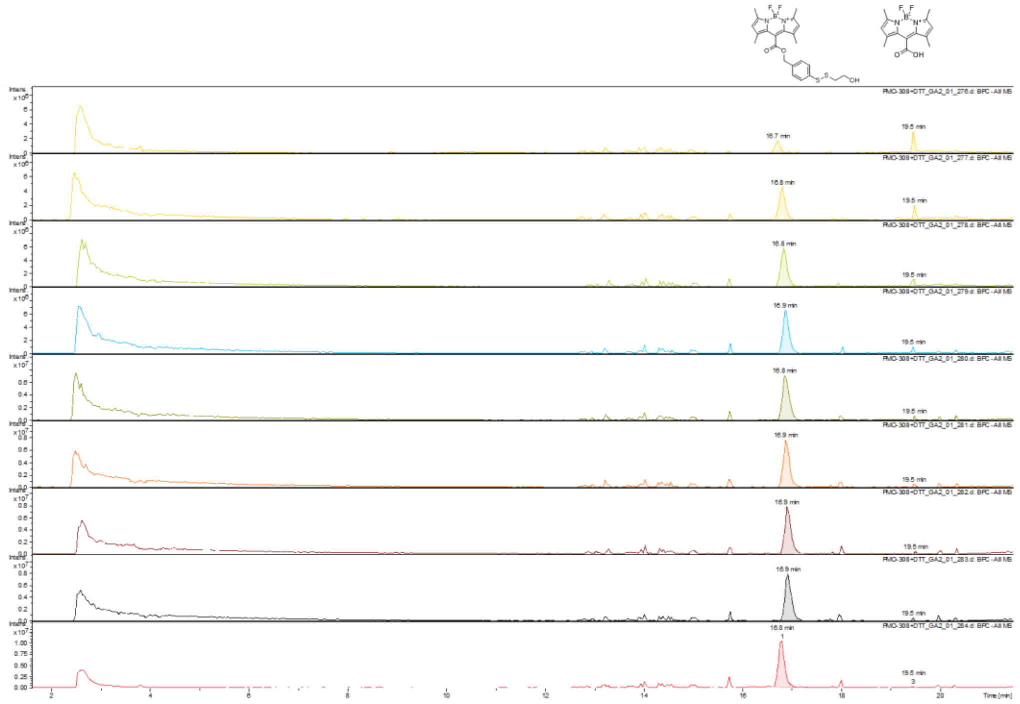

B

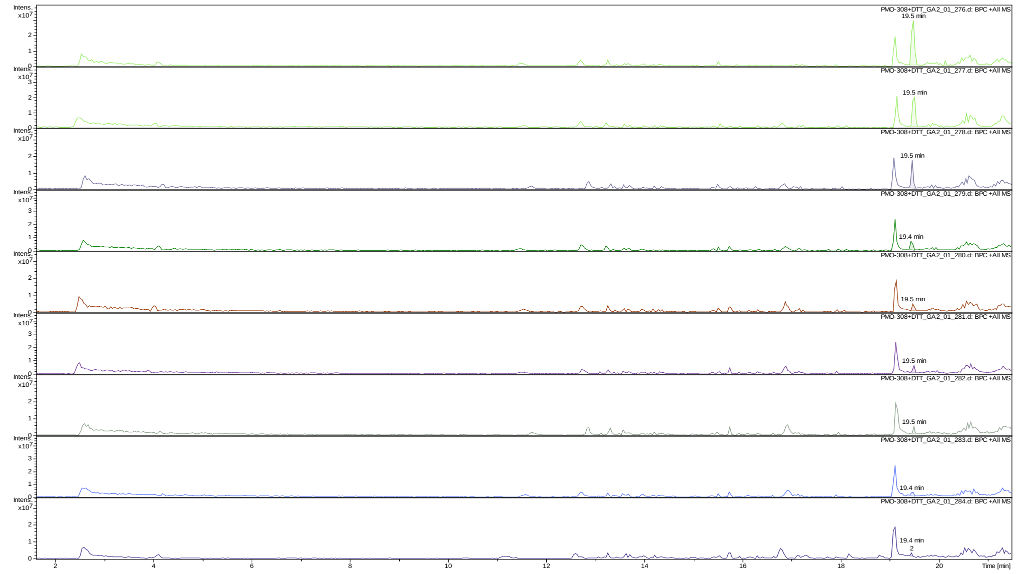

Fig. 4S HPLC conditions in (A) negative and (B) positive detection modes: flow rate : 0.6mL/min V_{inj} : 2µL, solvent A : H_2O+0.1 % HCOOH, solvent B : CH_3CN+ 0.1 % HCOOH (see gradient conditions below), column temperature 30°C (column: 5µC18 EVO PHENOMENEX KINETEX 150*3mm 100A), sampler temperature 25°C. MS conditions: target trap : 400 m/z , range masse : 100-800 m/z, nebulizer : 50 psi, dry gas : 10 L/min, dry temp : 200°C, polarity: alternative positive/negative.

HPLC gradient conditions:

t (min)	Solvent A (%)	Solvent B (%)
-7	95	5
0	95	5
10	85	15
20	5	95
25	0	100

1.3. Cellular experiments

HeLa cells were seeded one day before in a 96-well plate (5 000 cells per well) in DMEM containing 10% Foetal Bovine Serum (DMEM-FBS). The day of the experiment, medium was changed to DMEM-FBS supplemented with 0.1 % Pluronic Acid (DMEM-FBS-PA) and the cells were incubated for 1 h with the different compounds at 20 µM in DMEM-FBS-PA. After two washes with PBS, fresh DMEM-FBS was provided, and the cells were incubated for 48 h in an Incucyte automated microscope at 37°C with 5% CO_2. Every 4 h, images were taken in each well and cell confluence determined by automated image analysis. The increase of cell confluence reflects cell growth in all conditions except for compounds **9** and **13** that drastically reduced cell density and growth.

Staphylococcus aureus Wichita (ATCC29213) cultures were grown to late exponential phase in BHI medium (OD = 3.6 at 600 nm). The bacteria were incubated for 15 min with compounds at 100 µM in BHI supplemented with 0.1 % Pluronic Acid. They were then diluted 100-times in fresh BHI in a P96-well plate that was incubated for 8 h in a plate absorbance reader at 37°C with shaking. Every 10 min, ODs were measured at 595 nm. The increase of OD reflects bacteria growth in all conditions.

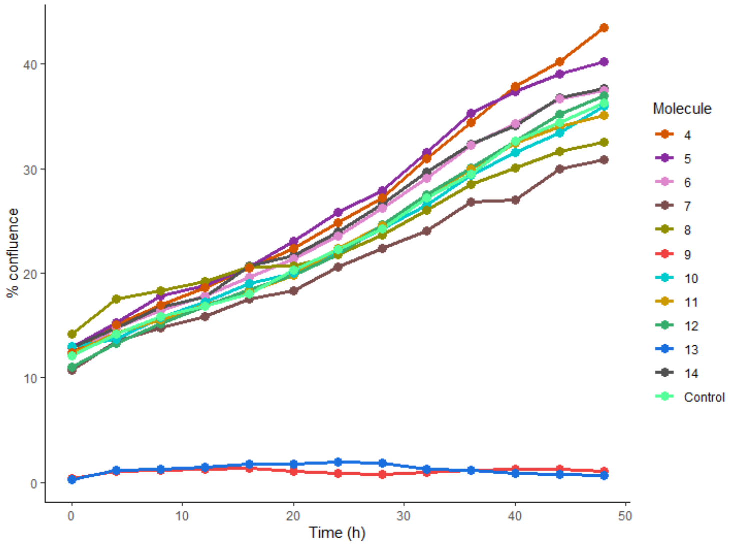

Fig. 5S Monitoring of human HeLa cells growth after 1 h incubation with the molecules at 20 µM.

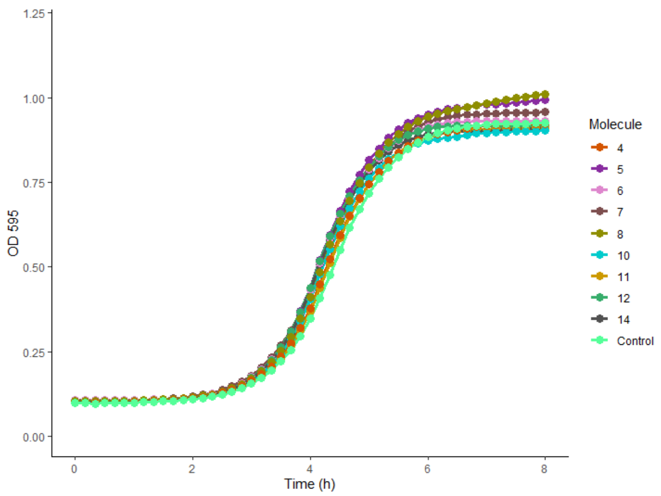

Fig. 6S Monitoring of *S. aureus* growth after 15 min incubation with the molecules at 100 µM.

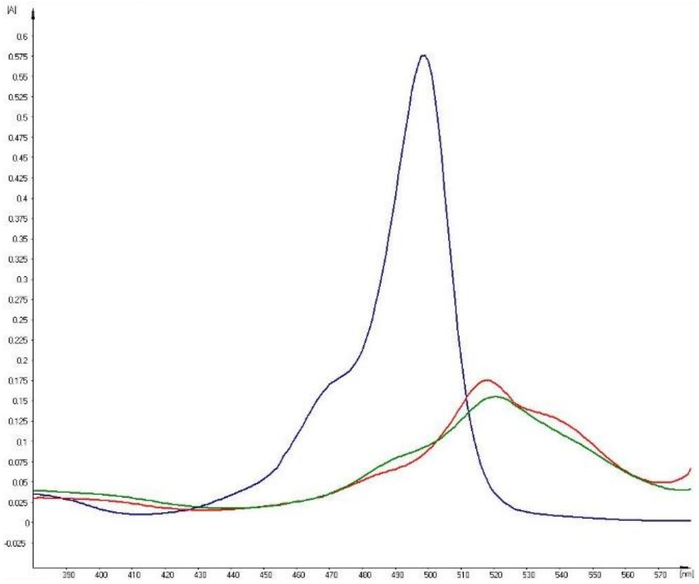

Fig. 7S Absorbance spectra of compounds **4**, **7**, and **8** (blue, red and green, respectively) record at 10 µM in PBS.

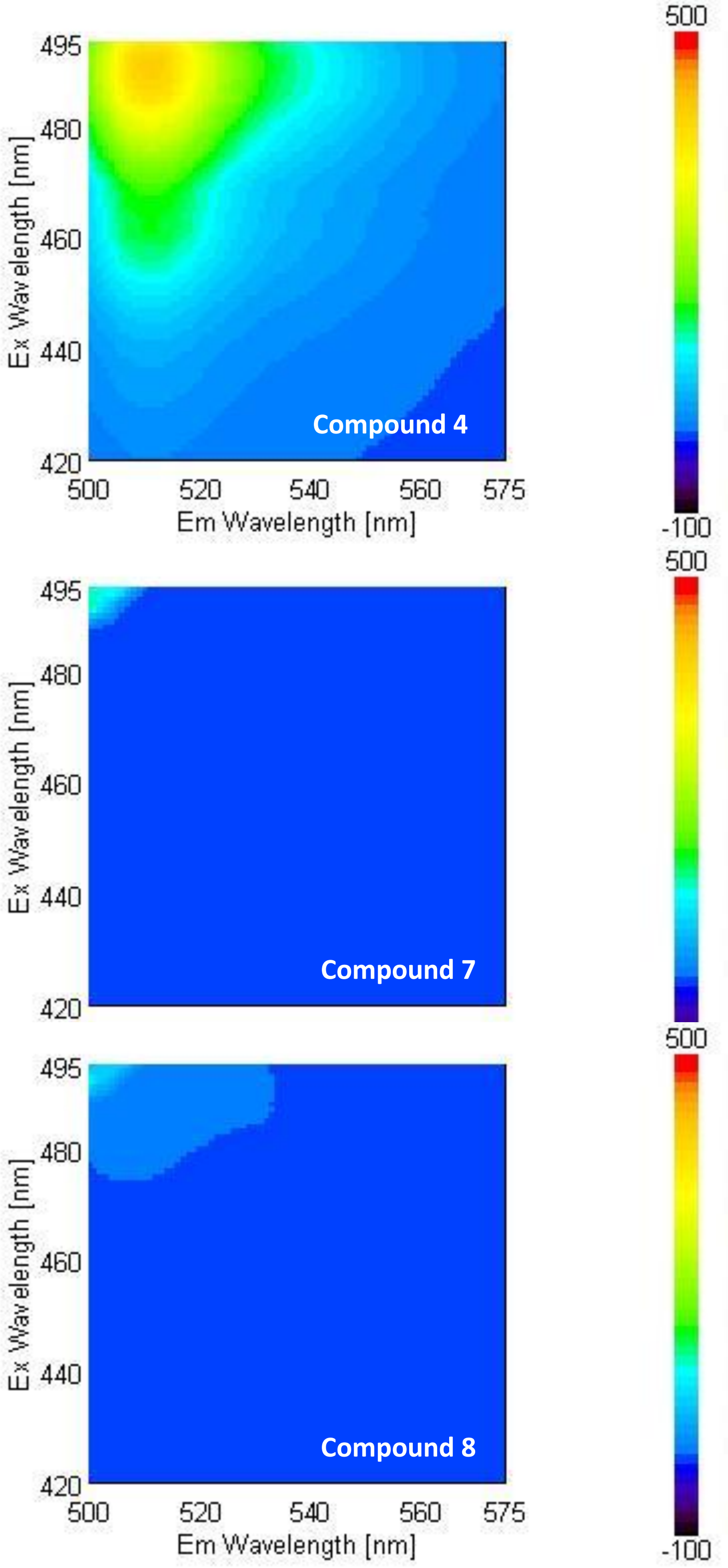

Fig. 8S 3D fluorescence spectra of compounds **4**, **7**, and **8** recorded at 10 µM in PBS. Emission spectra from 500 to 575 nm were acquired for every emission wavelength ranging from 420 to 495 nm. No maxima of excitation were observed for wavelengths ranging from 495 to 530 nm.

A

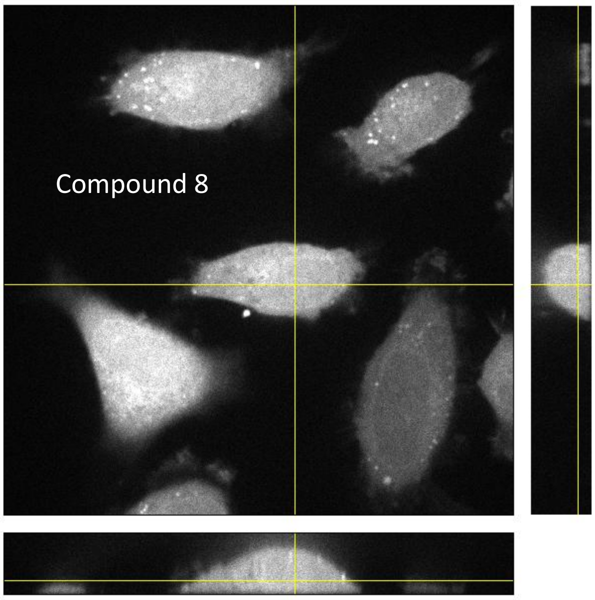

B

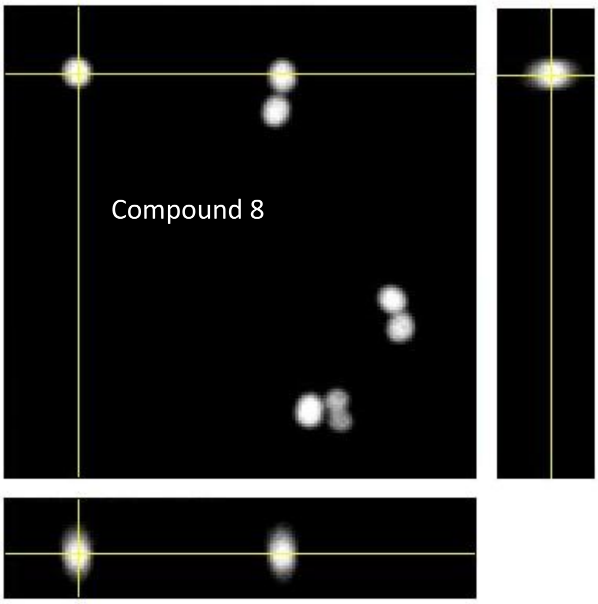

Fig. 9S Confocal fluorescence microscopy on HeLa (A) and *S. aureus* (B) cells showing homogeneous cytoplasmic labeling with compound **8**. Cells were incubated for 10 to 15 min with compound **8** at 100 µM in culture medium and observed with a confocal spinning disc microscope after two washes with PBS and addition of fresh medium. A 488 nm LED combined with standard GFP filters, 60× objective, 50 ms exposure and 0.2 µm z-steps were used for HeLa cells while 100× objective, 200 ms exposure and 0.132 µm z-steps were used for bacteria cells. 3D reconstruction and projections were performed using FIJI software.

1.4. Conditions to determine the Minimum Inhibitory Concentrations (MICs)

MICs of the compounds **15 a-c**, **16 a-c** and **17 a-c** were determined in triplicate against reference strains used for antibiotic susceptibility testing assays: *S. aureus* ATCC29213, *E. coli* ATCC 25922, *P. aeruginosa* ATCC27853 and *Bacillus subtilis* ATCC6633. Commercial levofloxacin (LEVOFLOXACINE KABI 5 mg/ml) was used as control. Minimum inhibitory concentrations (MICs) were determined in broth microdilution method, as recommended by the Clinical and Laboratory Standards Institute (CLSI; M07-Ed11) and/or EUCAST (European committee on antimicrobial susceptibility testing) using a cation-adjusted Mueller–Hinton 2 (MH2) broth (Millipore, 90922). For each bacterial strain, one row of a 96-well microtiter plate was filled with 75 µL of a 2X twofold serial dilutions of the compound to be tested, in MH2 broth with 8% DMSO, before addition of 75 µL of bacterial suspension calibrated at 10^6 CFU/mL as to obtain final concentrations ranging from 0.06 to 32 mg/L or 0.5 to 128 mg/L in 4% DMSO and final inoculum of 5×10^5 CFU/mL. A well containing DMSO at final concentrations found in working solutions of the tested compounds was used as DMSO toxicity controls (positive growth control) and a well with the culture medium and the last antibiotic dilution without bacterial suspension was used as a negative control. Microplates were incubated at 37°C in ambient air. MICs were read visually after 20 hours of incubation of cultures and corresponded to the minimum concentration of the compound that allowed complete inhibition of visual growth of the bacteria.

Additional MIC testing assays were then performed with compound **16a-c** with clinical E. coli strains[2] (*E .coli* CHUGA-2018-Eco1 ; *E. coli* CHUGA-2018-Eco2 ; *E. coli* CHUGA-2018-Eco10 ; *E. coli* CHUGA-2018-Eco24) with known fluoroquinolone resistance mechanism as their whole genome has been sequenced (ENA project accession number PRJEB72649) and reference *S. aureus* strains (*S. aureus* SA-1199B ; *S. aureus* ATCC 106414 ; *S. aureus* ATCC BAA44 ; *S. aureus* ATCC 700698 ; *S. aureus* ATCC 700699) showing variable resistance levels to levofloxacin.

1.5. Eukaryotic cell infection and compounds evaluation

A549 human alveolar basal epithelial cell line was cultured in RPMI-1640 medium (Sigma-Aldrich, 58758) supplemented with 10% fetal bovine serum (Gibco, 2726081). One day before infection, A549 cells were seeded at confluence in 96-well plates (Greiner, 655090). The infection was performed at a multiplicity of infection (MOI) of 10 using the *S. aureus* strain HG003_pVT13, which constitutively expresses the GFP. Following 2 h of co-incubation, continuous lysostaphin treatment was applied at 10 µg/mL to eradicate extracellular *S. aureus*, along with a range of concentrations of compounds **15a-c**, **16a-c**, and **17a-c** (from 0.06125 to 128 µg/mL) where indicated. 24 h after the start of compounds treatment, A549 cells were lysed in 200 µL H_2O to collect intracellular *S. aureus* survivors. 5 µL of the resulting lysates were spotted on agar plates, with 6 replicates per well. Colony numbers were enumerated per spot, except in cases of bacterial lawn formation, for which an arbitrary value of 5000 was assigned.

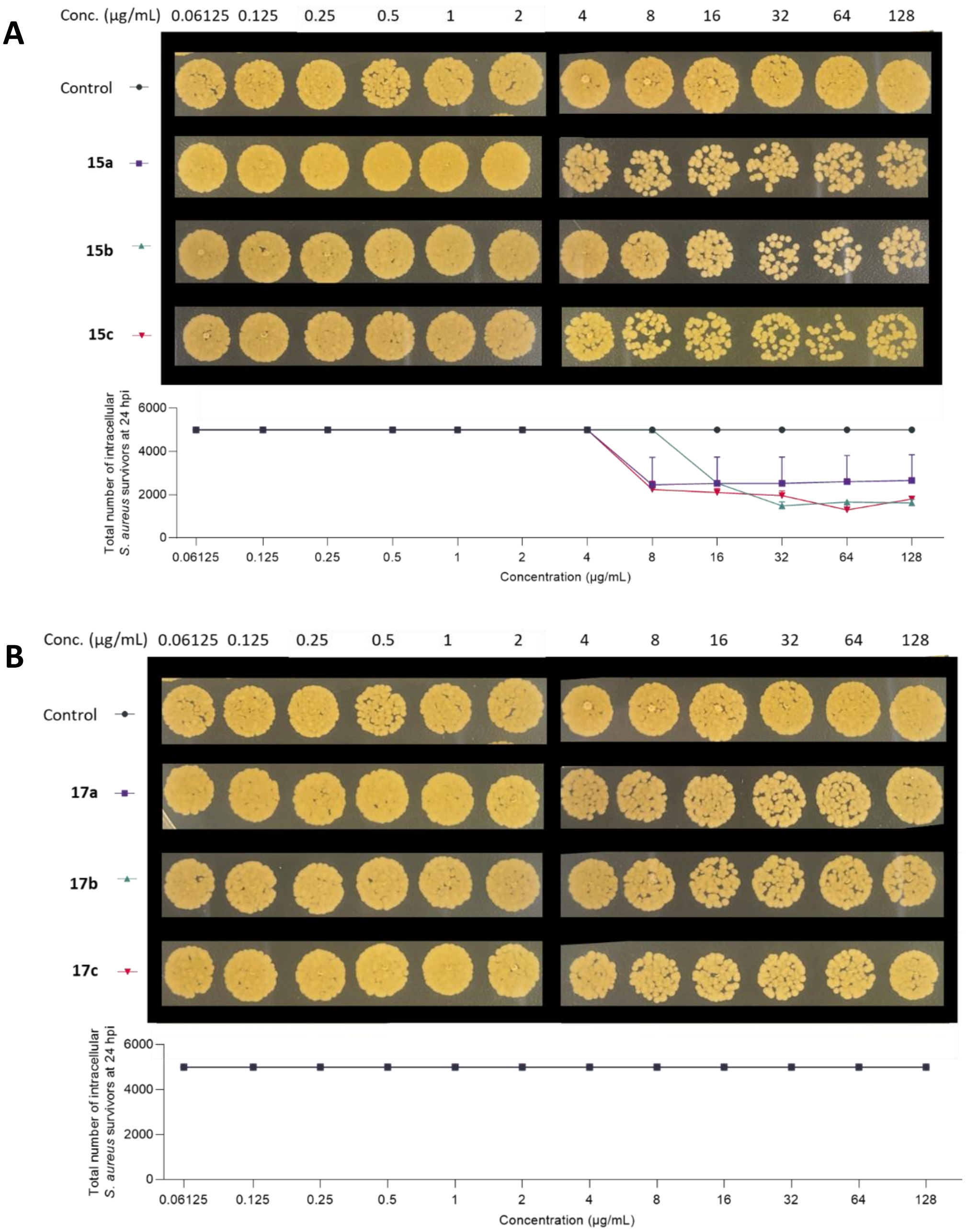

Fig. 10S Ability of compounds **15a-c** and **17a-c** to kill intracellular *S. aureus*. **(a, b)** A549 cells were infected at a MOI of 10 with *S. aureus* strain HG003_pVT13. After 2 h, lysostaphin treatment (10 µg/mL) was applied to eradicate extracellular *S. aureus*. Simultaneously, compounds (**a**) **15a-c** or (**b**) **17a-c** at a range of concentrations (0.06125 to 128 µg/mL) were added to the medium. After 24 h of treatment, intracellular *S. aureus* were collected and plated on agar plates. Colony numbers were enumerated per spot, except in cases of bacterial lawn formation, for which an arbitrary value of 5000 was assigned.

2. **Synthesis and characterization of molecules 6 to 17b**

The reactions were monitored by thin-layer chromatography (TLC) on Macherey-Nagel silica gel 60 with UV detection at 254 nm. Flash column chromatographies (FCC) were performed using Macherey-Nagel silica gel 60 M. Nuclear magnetic resonance (NMR) spectra were recorded either on a Brüker Avance Neo (400 MHz for ^{1}H, 100 MHz for ^{13}C) or on a Brüker Avance III (500 MHz for ^{1}H, 126 MHz for ^{13}C). High resolution mass spectrometry (HRMS) spectra were recorded by the Institut de Chimie Moléculaire de Grenoble (ICMG). The spectra were recorded on a LTQ Orbitrap XL Thermo Scientific with ElectroSpray Ionization (ESI).

4-Nitrobenzyl-5,5-difluoro-1,3,7,9-tetramethyl-5H-4λ4,5λ4-dipyrrolo[1,2-c:2',1'-f][1,3,2]diazaborinine-10-carboxylate **6**

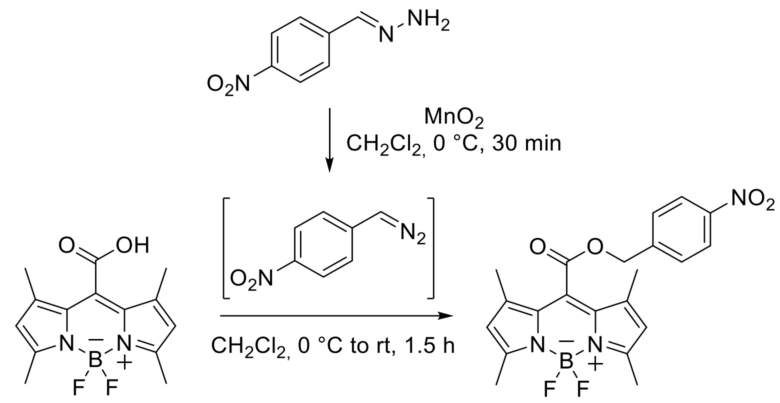

4 **6**, 51%

An oven-dried flask containing anhydrous MnO_2 (29.8 mg, 0.342 mmol) is wrapped in aluminum foil and placed in an ice-bath. Then a solution of 4-nitrobenzaldehyde hydrazone (30 mM in CH_2Cl_2, 2 mL, 10.0 mg, 0.06 mmol) is added and stirred for 30 min in the dark. In the meantime, a solution of BODIPY **4** (11 mM in CH_2Cl_2, 3.1 mL, 10 mg, 0.034 mmol) is placed into a flask wrapped in aluminum foil and cooled to 0 °C. Then the solution of hydrazone is filtered in the dark at 0 °C through a plug of celite, to obtain a bright-orange diazomethane solution. This solution is added to the solution of **4** and the mixture is allowed to warm to rt. After 1.5 h, the mixture is diluted with CH_2Cl_2 and the reaction is stopped by addition of citric acid (4wt%). The organic layer is separated, washed with an aqueous $NaHCO_3$ (4wt%) solution, H_2O and brine. The separated organic layer is dried with $MgSO_4$ and concentrated *in vacuo*. The residue was eluted from a column of silica gel with 10% of EtOAc in cyclohexane to afford BODIPY **6** (7.4 mg, 51%) as a red iridescent solid. **^{1}H NMR** ($CDCl_3$, 400 MHz) δ 8.26 (d, 2H, *J* = 8.8 Hz, 2 CH_{ar}), 7.62 (d, 2H, *J* = 8.8 Hz, 2 CH_{ar}), 6.06 (s, 2H, 2 CH_{ar}), 5.47 (s, 2H, CH_2), 2.53 (t, 6H, *J* = 1.2 Hz, 2 CH_3), 2.02 (s, 6H, 2 CH_3). **^{13}C NMR** ($CDCl_3$, 100 MHz) δ 164.9 (CO), 158.1 (t, *J* = 2.3 Hz, 2 C_Q), 148.2 (C_Q), 140.9 (dd, *J* = 1.5, 4.1 Hz, 2 C_Q), 140.6 (C_Q), 129.3 (2 CH), 128.8 (dt, *J* = 1.4, 3.8 Hz, 2 C_Q), 127.9 (C_Q), 124.1 (2 CH), 121.5 (dd, *J* =1.5, 5.1 Hz, 2 CH), 66.8 (CH_2), 14.9 (t, *J* = 2.1 Hz, 2 CH_3),12.8 (2 CH_3). **HRMS** (ESI) *m/z* calcd. for $C_{21}H_{21}O_4N_3BF_2$ $[M+H]^+$ 428.1588, found 428.1582.

2-((2-Hydroxyethyl)disulfanyl)ethyl-5,5-difluoro-1,3,7,9-tetramethyl-5H-4λ4,5λ4-dipyrrolo[1,2-c:2',1'-f][1,3,2]diazaborinine-10-carboxylate **7**

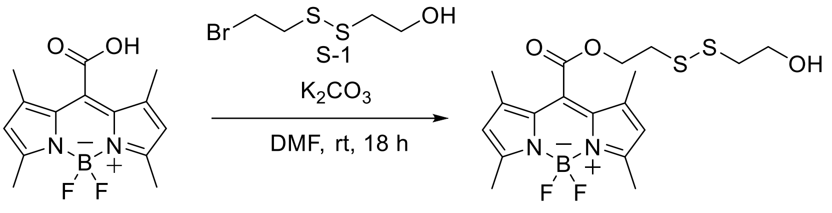

4 **7**, 19%

To a solution of BODIPY **4** (20 mg, 0.068 mmol) and bromide **S-1** (30 mg, 0.137 mmol) in dry DMF at r.t. was added K_2CO_3 (19 mg, 0.137 mmol). The resulting mixture was stirred 18 h at rt and then water and CH_2Cl_2 were added. The aqueous phase was extracted 3 times with CH_2Cl_2 and the resulting organic phase was dried over $MgSO_4$, filtered and concentrated *in vacuo*. The residue was eluted from a column of silica gel with 33% to 50% of EtOAc in cyclohexane to afford BODIPY **7** (5.5 mg, 19%) as a red iridescent solid.**^{1}H NMR** ($CDCl_3$, 500 MHz) δ 6.07 (s, 2H, CH_{ar}), 4.60 (t, 2H, *J* = 6.5 Hz, CH_2), 3.87 (t, 2H, *J* = 5.8 Hz, CH), 3.05 (t, 2H, *J* = 6.5 Hz, CH_2), 2.88 (t, 2H, *J* = 5.8 Hz, CH_2), 2.53 (s, 6H, 2 CH_3), 2.15 (s, 6H, 2 CH_3). **^{13}C NMR** ($CDCl_3$, 125 MHz) δ 165.3 (CO), 158.0 (2 C_Q), 141.2 (2 C_Q), 128.9 (2 C_Q), 128.5 (C_Q), 121.5 (2 CH-ar), 64.7 (CH_2), 60.4 (CH_2), 41.6 (CH_2), 36.5 (CH_2), 14.9 (2 CH_3), 13.0 (2 CH_3). **HRMS** (ESI) *m/z* calcd. for $C_{18}H_{22}O_3N_2BF_2S_3$ $[M-H]^-$ 427.1139, found 427.1140.

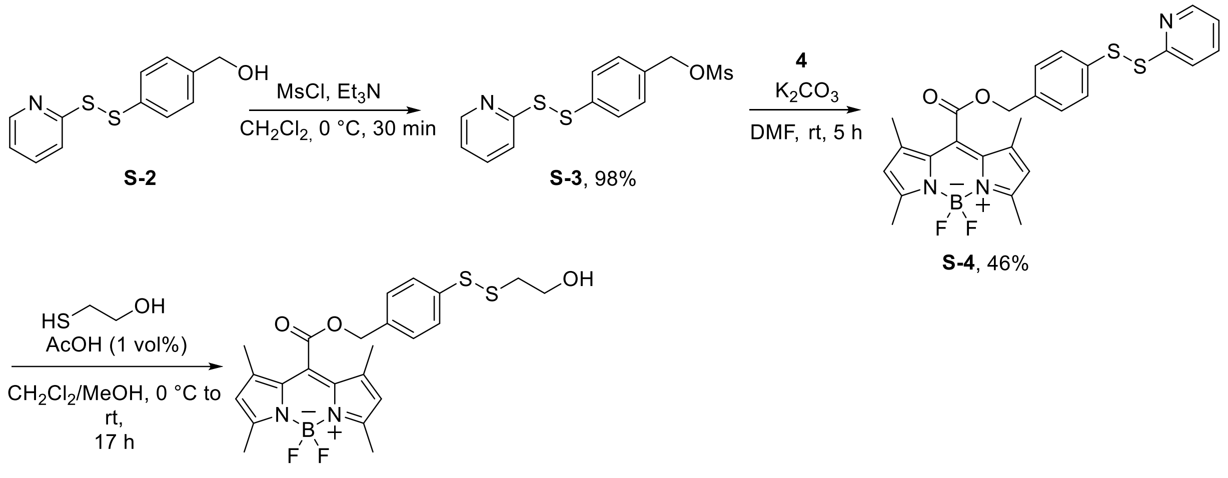

S-2 **S-3**, 98% **S-4**, 46% **8**, 85%

(4-(Pyridin-2-yldisulfanyl)phenyl)methanol **S-3**

To a flame-dried flask were added **S-2**[3] (100 mg, 0.4 mmol), triethylamine (108 µL, 0.8 mmol) and anhydrous CH_2Cl_2 (1.8 mL). The reaction was cooled to 0°C and MsCl (44 µL, 0.56 mmol) was added dropwise. The mixture was stirred at 0°C for 30 min. Then, the reaction mixture was diluted with EtOAc and washed with HCl (1M) and with a saturated $NaHCO_3$ solution. The combined organic layers were dried over $MgSO_4$, filtered and concentrated *in vacuo* to afford **S-3** (128 mg, 98%) as a slightly yellow oil. **^{1}H NMR** ($CDCl_3$, 400 MHz) δ 8.49-8.47 (m, 1H, CH_{ar}), 7.65-7.58 (m, 1H, CH_{ar}), 7.56 (d, 2H, *J* = 8.4 Hz, CH_{ar}), 7.36 (d, 2H, *J* = 8.4 Hz, CH_{ar}), 7.12 (ddd, 1H, *J* = 1.9, 4.8, 6.6 Hz, CH_{ar}), 5.19 (s, 2H, CH_2O), 2.93 (s, 3H, CH_3). **^{13}C NMR**

($CDCl_3$, 100 MHz) δ 159.2 (C_Q), 149.9 (CH), 138.1 (C_Q), 137.5 (CH), 132.5 (C_Q), 129.7 (2 CH), 127.5 (2 CH), 121.3 (CH), 119.8 (CH), 70.8 (CH_2), 38.5 (CH_3).

4-(Pyridin-2-yldisulfaneyl)benzyl 5,5-difluoro-1,3,7,9-tetramethyl-5H-4λ4,5λ4-dipyrrolo[1,2-c:2',1'-f][1,3,2]diazaborinine-10-carboxylate **S-4**

To a solution of BODIPY **4** (50 mg, 0.17 mmol) and mesylate **S-3** (51 mg, 0.154 mmol) in dry DMF (1 mL) at rt was added K_2CO_3 (63 mg, 0.46 mmol). The resulting mixture was stirred 5 h at rt and then water and CH_2Cl_2 were added. The aqueous phase was extracted 3 times with CH_2Cl_2 and the resulting organic phase was dried over $MgSO_4$, filtered and concentrated *in vacuo*. The residue was eluted from a column of silica gel with 25% of EtOAc in cyclohexane to afford BODIPY **S-4** (37 mg, 46%) as a red iridescent solid. **^{1}H NMR** ($CDCl_3$, 400 MHz): δ 8.47 (d, 1H, *J* = 4.8 Hz, CH_{ar}), 7.57-7.64 (m, 2H, 2 CH_{ar}), 7.53 (d, 2H, *J* = 8.3 Hz, 2 CH_{ar}), 7.36 (d, 2H, *J* = 8.3 Hz, 2 CH_{ar}), 7.10 (ddd, 1H, *J* = 1.9, 4.8, 6.5 Hz, CH_{ar}), 6.01 (s, 2H, 2 CH_{ar}), 5.32 (s, 2H, CH_2), 2.50 (s, 6H, 2 CH_3) 1.97 (s, 6H, 2 CH_3),. **^{13}C NMR** ($CDCl_3$, 100 MHz) δ 165.0 (CO), 159.2 (C_Q), 157.8 (2 C_Q), 149.7 (CH), 141.1 (2 C_Q), 137.5 (CH, C_Q), 132.8 (C_Q), 130.1 (2 CH), 128.9 (2 C_Q), 128.6 (C_Q), 127.6 (2 CH), 121.3 (d, *J* = 2.5 Hz, 2 CH), 121.2 (CH), 119.8 (CH), 68.0 (CH_2), 14.9 (t, *J* = 1.9 Hz, 2 CH_3),12.8 (2 CH_3). **HRMS** (ESI) calcd for $C_{26}H_{25}O_2N_3S_2BF_2$ $[M+H]^+$ 524.1444, found 524.1431.

4-((2-Hydroxyethyl)disulfanyl)benzyl-5,5-difluoro-1,3,7,9-tetramethyl-5H-4λ4,5λ4-dipyrrolo[1,2-c:2',1'-f][1,3,2]diazaborinine-10-carboxylate **8**

To activated disulfide **S-4** (10 mg, 0.019 mmol) in CH_2Cl_2 (1 mL) was added AcOH (1vol%) as catalyst. The solution was cooled to 0 °C and 2-mercaptoethanol (0.023 mmol, 1.6 µL) dissolved in MeOH (1 mL) was added. The reaction was allowed to warm to r.t. over 17 h. Upon completion of the reaction, the mixture was diluted with CH_2Cl_2 (20 mL) and washed with 1 M NaOH. The organic layer was dried with $MgSO_4$ and concentrated *in vacuo*. The residue was eluted from a column of silica gel with 20% of EtOAc in cyclohexane to afford BODIPY **8** (9.7 mg, 85%) as a red iridescent solid. **^{1}H NMR** ($CDCl_3$, 400 MHz) δ 7.57 (d, 2H, *J* = 8.3 Hz, 2 CH_{ar}), 7.40 (d, 2H, *J* = 8.3 Hz, 2H, 2 CH_{ar}), 6.04 (s, 2H, 2 CH_{ar}), 5.35 (s, 2H, CH_2), 3.85 (t, 2H, *J* = 5.8 Hz, CH_2O), 2.89 (t, 2H, *J* = 5.8 Hz, CH_2S), 2.52 (t, 6H, *J* = 1.3 Hz, 2 CH_3), 2.02 (s, 6H, 2 CH_3). **^{13}C NMR** ($CDCl_3$, 100 MHz) δ 165.1 (CO), 157.9 (2 C_Q), 141.2 (2 C_Q), 138.6 (C_Q), 130.1 (2 CH_{ar}), 132.6 (C_Q), 128.9 (bs, 2 C_Q), 128.7 (C_Q), 127.9 (2 CH_{ar}), 121.4 (d, *J* = 2.7 Hz, 2 CH_{ar}), 68.1 (CH_2), 60.1 (CH_2), 41.5 (CH_2), 14.9 (t, *J* = 2 Hz, 2 CH_3),12.9 (2 CH_3). **HRMS** (ESI): calcd for $C_{23}H_{26}O_3N_2BF_2S_2$ $[M+H]^+$ 491.1441, found 491.1430.

4-((2-(2-Hydroxyethoxy)ethyl)disulfanyl)benzyl 5,5-difluoro-1,3,7,9-tetramethyl-5H-4λ4,5λ4-dipyrrolo[1,2-c:2',1'-f][1,3,2]diazaborinine-10-carboxylate **9**

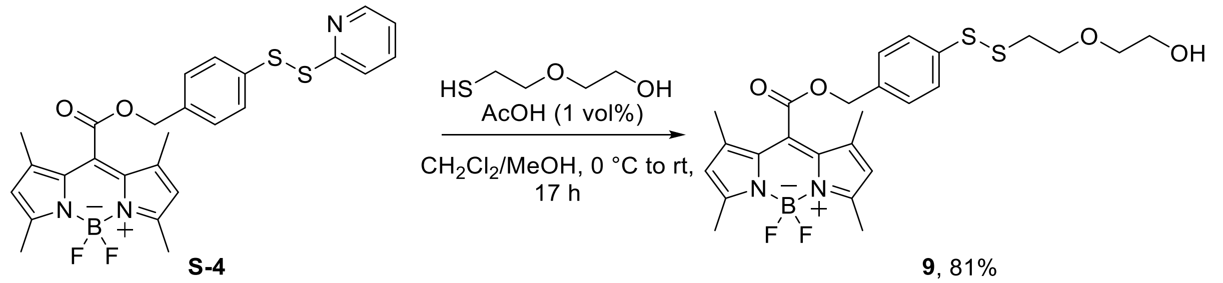

S-4 **9**, 81%

To activated disulfide **S-4** (37 mg, 0.079 mmol) in CH_2Cl_2 (1 mL) was added AcOH (1vol%) as catalyst. The solution was cooled to 0 °C and 2-mercaptoethanol (0.085 mmol, 10.5 mg) dissolved in MeOH (1 mL) was added. The reaction was allowed to warm to rt over 17 h. Upon completion of the reaction, the mixture was diluted with CH_2Cl_2 (20 mL) and washed with 1 M NaOH. The organic layer was dried with $MgSO_4$ and concentrated *in vacuo*. The residue was eluted from a column of silica gel with 25% of EtOAc in cyclohexane to afford BODIPY **9** (30 mg, 81%) as a red iridescent solid. **^{1}H NMR** ($CDCl_3$, 400 MHz) δ 7.56 (d, 2H, J = 6.9 Hz, 2 CH_{ar}), 7.39 (d, 2H, J = 6.9 Hz, 2 CH_{ar}), 6.03 (s, 2H, 2 CH_{ar}), 5.35 (s, 2H, CH_2), 3.72-3.70 (m 4H, 2 CH_2O), 3.52 (t, 2H, J = 4.3 Hz, CH_2S), 3.52 (t, 2H, J = 4.3 Hz, CH_2S), 2.93 (t, 2H, J = 5.6 Hz, CH_2S), 2.52 (s, 6H, 2 CH_3), 2.02 (s, 6H, 2 CH_3). **^{13}C NMR** ($CDCl_3$, 100 MHz) δ 161.1 (CO), 157.9 (2 C_Q), 141.2 (2 C_Q), 138.9 (2 C_Q), 132.3 (C_Q), 130.0 (2 CH_{ar}), 128.9 (C_Q), 128.7 (C_Q), 127.5 (2 CH_{ar}), 121.4 (2 CH_{ar}), 72.2 (CH_2), 68.9 (CH_2), 68.1 (CH_2), 61.9 (CH_2), 38.6 (CH_2), 14.9 (2 CH_3), 12.9 (2 CH_3). **HRMS** (ESI): calcd for $C_{25}H_{28}O_4N_2BF_2S_2$ $[M-H]^-$ 533.1558, found 533.1548.

4-(Phenyldisulfanyl)benzyl 5,5-difluoro-1,3,7,9-tetramethyl-5H-4λ4,5λ4-dipyrrolo[1,2-c:2',1'-f][1,3,2]diazaborinine-10-carboxylate **10**

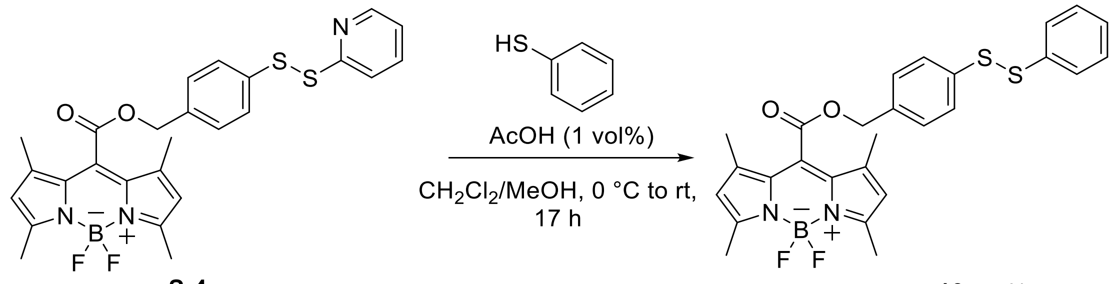

S-4 **10**, 55%

To activated disulfide **S-4** (10 mg, 0.019 mmol) in CH_2Cl_2 (1 mL) was added AcOH (1vol%) as catalyst. The solution was cooled to 0 °C and thiophenol (0.023 mmol, 2.5 mg) dissolved in MeOH (1 mL) was added. The reaction was allowed to warm to rt over 17 h. Upon completion of the reaction, the mixture was diluted with CH_2Cl_2 (20 mL) and washed with 1 M NaOH. The organic layer was dried with $MgSO_4$ and concentrated *in vacuo*. The residue was eluted from a column of silica gel with 10% of EtOAc in cyclohexane to afford BODIPY **10** (5.5 mg, 55%) as a red solid. **^{1}H NMR** ($CDCl_3$, 500 MHz) δ 7.52 (d, 2H, J = 8.2 Hz, 2 CH_{ar}), 7.47 (dd, 2H, J = 7.5, 1.5 Hz, 2 CH_{ar}), 7.37 (d, 2H, J = 8.2 Hz, 2 CH_{ar}), 7.30 (t, 2H, J = 7.5, 7.5 Hz, 2 CH_{ar}), 7.24 (tt, 1H, J = 7.5, 1.5 Hz, CH_{ar}), 6.02 (s, 2H, 2 CH_{ar}), 5.34 (s, 2H, CH_2), 2.52 (t, 6H, J = 1.3 Hz, 2 CH_3), 1.98 (s, 6H, 2 CH_3). **^{13}C NMR** ($CDCl_3$, 125 MHz) δ 165.1 (CO), 157.9 (2 C_Q), 141.2 (2 C_Q), 138.5 (CH), 136.7 (CH), 132.6 (CH), 130.1 (2 CH), 129.3 (2 CH), 128.9 (bs, 2 C_Q), 128.7 (C_Q), 127.7 (2 CH), 127.7 (2 CH), 127.5 (CH), 121.4 (d, J = 3.4 Hz, 2 CH), 68.1 (CH_2), 14.9 (t, J = 2 Hz, 2 CH_3),12.8 (2×CH_3). **HRMS** (ESI) calcd for $C_{27}H_{26}O_2N_2BF_2S_2$ $[M+H]^+$ 523.1492, found 523.1493.

4-(Benzyldisulfanyl)benzyl 5,5-difluoro-1,3,7,9-tetramethyl-5H-4λ4,5λ4-dipyrrolo[1,2-c:2',1'-f][1,3,2]diazaborinine-10-carboxylate **11**

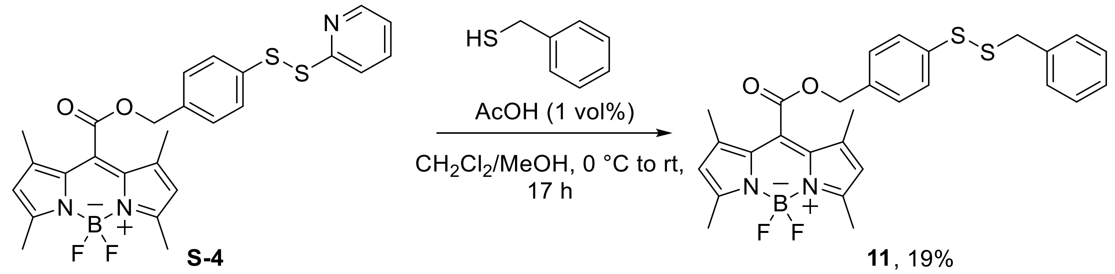

To activated disulfide **S-4** (10 mg, 0.019 mmol) in CH_2Cl_2 (1 mL) was added AcOH (1vol%) as catalyst. The solution was cooled to 0 °C and benzyl mercaptan (0.023 mmol, 2.9 mg) dissolved in MeOH (1 mL) was added. The reaction was allowed to warm to rt over 17 h. Upon completion of the reaction, the mixture was diluted with CH_2Cl_2 (20 mL) and washed with 1 M NaOH. The organic layer was dried with $MgSO_4$ and concentrated *in vacuo*. The residue was eluted from a column of silica gel with 10% of EtOAc in cyclohexane to afford BODIPY **11** (5.5 mg, 19%) as a red solid. **^{1}H NMR** ($CDCl_3$, 500 MHz) δ 7.44 (d, 2H, *J* = 8.3 Hz, 2 CH_{ar}), 7.34 (d, 2H, *J* = 8.3 Hz, 2 CH_{ar}), 7.21 – 7.31 (m, 5H, 5 CH_{ar}), 6.04 (s, 2H, 2 CH_{ar}), 5.34 (s, 2H,CH_2), 3.93 (s, 2H, CH_2), 2.52 (t, *J* = 1.2 Hz, 6H, 2 CH_3), 2.03 (s, 6H, 2 CH_3). **^{13}C NMR** ($CDCl_3$, 125 MHz) δ 165.1 (CO), 157.9 (2 C_Q), 141.2 (2 C_Q), 138.7 (C_Q), 136.5 (C_Q), 132.2 (C_Q), 129.9 (2 CH), 129.5 (2 CH), 129.0 (2 C_Q), 128.8 (C_Q), 128.7 (2 CH), 12.9 (2×CH3, 6), 127.8 (CH), 127.6 (2 CH), 121.4 (d, *J* = 2.55 Hz, 2 CH), 68.2 (CH_2), 43.5 (CH_2), 14.9 (t, *J* = 2.06, 2.06 Hz, 2 CH_3). **HRMS** (ESI) calcd for $C_{28}H_{26}O_2N_2BF_2S_2$ $[M-H]^-$ 535.1503, found 535.1496.

4-(Dodecyldisulfanyl)benzyl 5,5-difluoro-1,3,7,9-tetramethyl-5H-4λ4,5λ4-dipyrrolo[1,2-c:2',1'-f][1,3,2]diazaborinine-10-carboxylate **12**

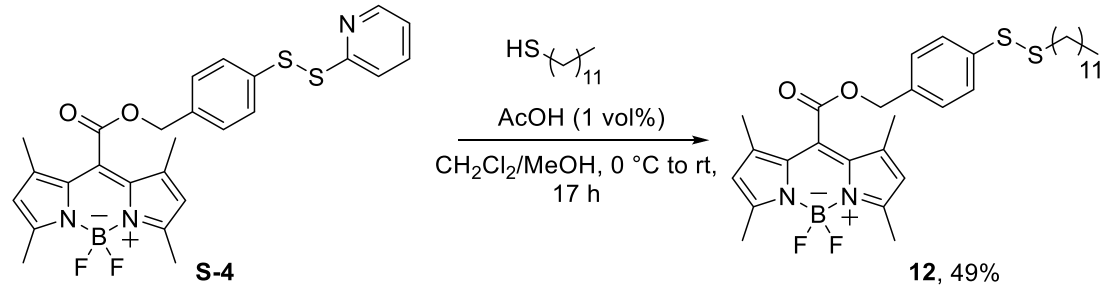

To activated disulfide **S-4** (10 mg, 0.019 mmol) in CH_2Cl_2 (1 mL) was added AcOH (1vol%) as catalyst. The solution was cooled to 0 °C and 1-dodecanethiol (0.023 mmol, 4.6 mg) dissolved in MeOH (1 mL) was added. The reaction was allowed to warm to rt over 17 h. Upon completion of the reaction, the mixture was diluted with CH_2Cl_2 (20 mL) and washed with 1 M NaOH. The organic layer was dried with $MgSO_4$ and concentrated *in vacuo*. The residue was eluted from a column of silica gel with 10% of EtOAc in cyclohexane to afford BODIPY **12** (5.7 mg, 49%) as a red solid. **^{1}H NMR** ($CDCl_3$, 500 MHz) δ 7.55 (d, 2H, *J* = 8.3 Hz, 2 CH_{ar}), 7.38 (d, 2H, *J* = 8.3 Hz, 2 CH_{ar}), 6.03 (s, 2H, 2 CH_{ar}), 5.35 (s, 2H, CH_2), 2.72 (t, 2H, *J* = 7.4 Hz, CH_2), 2.52 (s, 6H, 2 CH_3), 2.01 (s, 6H, 2 CH_3), 1.65 (p, 2H, *J* = 7.4 Hz, CH_2), 1.19 – 1.41 (m, 18H, 9 CH_2), 0.88 (t, 3H, *J* = 6.9 Hz, CH_3).**^{13}C NMR** ($CDCl_3$, 125 MHz) δ 165.0 (CO), 157.7 (2 C_Q), 141.08

(2 C_Q), 139.23 (C_Q), 131.85 (C_Q), 129.82 (2 CH), 128.81 (t, J = 2 Hz, 2 C_Q), 127.21 (2 CH), 128.63 (C_Q), 121.21 (d, J = 3.4 Hz, 2 CH), 68.05 (CH_2), 38.96 (CH_2), 31.92 (CH_2), 29.64 (CH_2), 29.63 (CH_2), 29.57 (CH_2), 29.48 (CH_2), 29.35 (CH_2), 29.16 (CH_2), 28.84 (CH_2), 28.49 (CH_2), 22.69 (CH_2), 14.77 (t, J = 2.3 Hz, 2 CH_3), 14.12 (CH_3), 12.73 (2 CH_3). **HRMS** (ESI): calcd for $C_{33}H_{46}O_2N_2BF_2S_2$ $[M+H]^+$ 615.3062, found 615.3044.

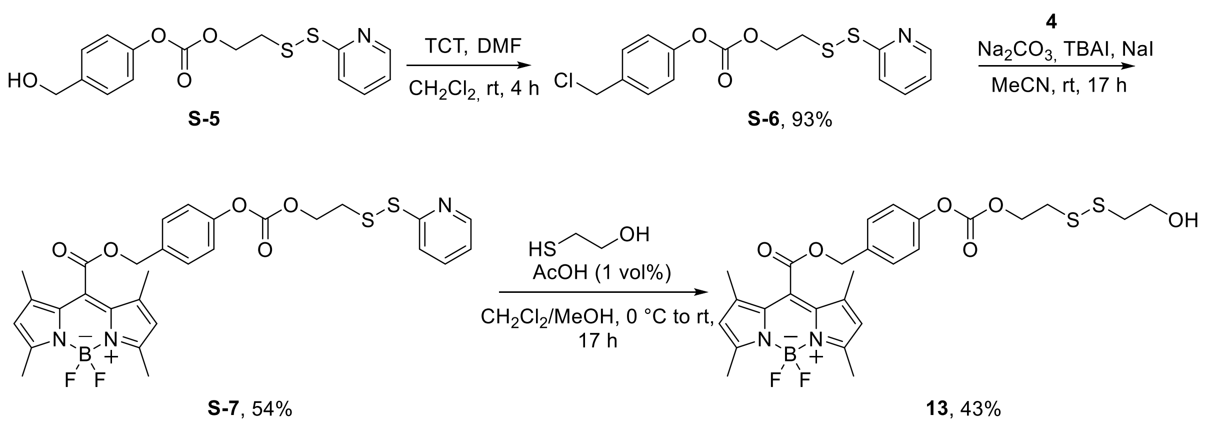

4-(Chloromethyl)phenyl (2-(pyridin-2-yldisulfanyl)ethyl) carbonate **S-6**

To a suspension of 2,4,6-trichloro-[1,3,5]triazine (82 mg, 0.44 mmol) in CH_2Cl_2 (1 mL) was added DMF (97 mg, 0.1 mL) at rt. After the formation of a white solid, the reaction was monitored (TLC) until complete disappearance of TCT, and a solution of alcohol **S-5**[4] (140 mg, 0.42 mmol) in CH_2Cl_2 (2 mL) was added. After 4 h, the reaction was stopped by the addition of water (10 mL) and CH_2Cl_2 (10 mL), then the organic phase was washed with sat. Na_2CO_3, followed by 1 M HCl and brine. The organic layers were dried with $MgSO_4$ and concentrated *in vacuo* to give chloride **S-6** (137 mg, 93%) as light-yellow oil. **^{1}H NMR** ($CDCl_3$, 400 MHz) δ 8.49 (ddd, 1H, J = 1.0, 1.8, 4.9 Hz, CH_{ar}), 7.69 (ddd, 1H, J = 1.0, 1.4, 8.1 Hz, CH_{ar}), 7.64 (ddd, 1H, J = 1.8, 7.1, 8.1 Hz, CH_{ar}), 7.40 (d, 2H, J = 8.7 Hz, 2 CH_{ar}), 7.16 (d, 2H, J = 8.7 Hz, 2 CH_{ar}), 7.11 (ddd, 1H, J = 1.4, 4.9, 7.1 Hz, CH_{ar}), 4.57 (s, 2H, CH_2), 4.51 (t, 2H, J = 6.5 Hz, CH_2), 3.14 (t, 2H, J = 6.5 Hz, CH_2). **^{13}C NMR** ($CDCl_3$, 100 MHz) δ 159.4 (C_Q), 153.3 (CO), 151.0 (C_Q), 149.8 (CH_{ar}), 137.3 (CH_{ar}), 135.5(C_Q), 129.9 (2 CH_{ar}), 121.4 (2 CH_{ar}), 121.2 (CH_{ar}), 120.2 (CH_{ar}), 66.3 (CH_2), 45.5 (CH_2), 37.1 (CH_2). **HRMS** (ESI) calcd for $C_{15}H_{15}O_3N_2ClS_2$ $[M+H]^+$ 356.0176, found 356.0172.

4-(((2-(Pyridin-2-yldisulfanyl)ethoxy)carbonyl)oxy)benzyl 5,5-difluoro-1,3,7,9-tetramethyl-5H-4λ4,5λ4-dipyrrolo[1,2-c:2',1'-f][1,3,2]diazaborinine-10-carboxylate **S-7**

To a solution of chloride **S-6** (30 mg, 0.084 mmol) in MeCN (5 mL) was added BODIPY **4** (29.6 mg, 0.101 mmol), fine powdered anhydrous Na_2CO_3 (25 mg, 0.236 mmol), tetrabutylammonium fluoride (3.1 mg, 0.0084 mmol) as catalyst and NaI (5 mg, 0.034 mmol) as initiator. After 17 h at rt, the reaction was stopped by the addition of H_2O (20 mL) followed by extraction with CH_2Cl_2. the combined organic layer was washed with 1 M NaOH, a solution of citric acid (10wt%) and brine. The organic layer was dried with $MgSO_4$ and concentrated *in vacuo*. The residue was eluted from a column of silica gel with 30% of EtOAc in cyclohexane to afford BODIPY **S-7** (27.8 mg, 54%) as a red solid. **^{1}H NMR** ($CDCl_3$, 400 MHz) δ 8.50 (dd, 1H, J = 4.8, 1.6 Hz, CH_{ar}), 7.72 (dd, 1H, J = 7.1, 1.6 Hz, CH_{ar}), 7.68 (ddd, 1H, J = 7.1, 1.5 Hz, CH_{ar}), 7.46 (d, 2H, J = 8.6 Hz, 2 CH_{ar}), 7.20 (d, 2H, J = 8.6 Hz, 2 CH_{ar}), 7.14 (ddd, 1H, J = 7.0, 4.8, 1.5 Hz, CH_{ar}), 6.03 (s, 2H, 2 CH_{ar}), 5.37 (s, 2H, CH_2), 4.52 (t, 2H, J = 6.4 Hz, CH_2), 3.16 (t, 2H, J = 6.4 Hz, CH_2), 2.51

(t, 6H, J = 1.2 Hz, 2 CH_3), 2.00 (s, 6H, 2 CH_3). **^{13}C NMR** ($CDCl_3$, 100 MHz) δ 165.0 (CO), 159.3 (C_Q), 157.9 (2 C_Q), 153.2 (CO), 151.6 (C_Q), 149.4 (CH_{ar}), 141.2 (2 C_Q), 137.8 (C_Q), 131.7 (C_Q), 130.6 (2 CH_{ar}), 128.9 (bs, 2 C_Q), 128.6 (C_Q), 121.6 (2 CH_{ar}), 121.4 (dd, J = 2.6, 1.2 Hz, 2 CH_{ar}), 121.3 (CH_{ar}), 120.6 (CH_{ar}), 67.8 (CH_2), 66.3 (CH_2), 37.2 (CH_2), 14.9 (t, J = 2.1 Hz, 2 CH_3), 12.8 (2 CH_3). **HRMS** (ESI) calcd for $C_{29}H_{27}O_5N_3BF_2S_2$ [M–H]$^-$ 610.1464, found 610.1454.

4-(((2-((2-Hydroxyethyl)disulfanyl)ethoxy)carbonyl)oxy)benzyl 5,5-difluoro-1,3,7,9-tetramethyl-5H-4λ4,5λ4-dipyrrolo[1,2-c:2',1'-f][1,3,2]diazaborinine-10-carboxylate **13**

To activated disulfide **S-7** (5 mg, 0.0081 mmol) in CH_2Cl_2 (1 mL) was added AcOH (1vol%) as catalyst. The solution was cooled to 0 °C and 2-mercaptoethanol (0.01 mmol, 0.8 mg) dissolved in MeOH (1 mL) was added. The reaction was allowed to warm to rt over 17 h. Upon completion of the reaction, the mixture was diluted with CH_2Cl_2 (20 mL) and washed with 1 M NaOH. The organic layer was dried with $MgSO_4$ and concentrated *in vacuo*. The residue was eluted from a column of silica gel with 33% of EtOAc in cyclohexane to afford BODIPY **13** (2.0 mg, 43%) as a red solid. **^{1}H NMR** ($CDCl_3$, 500 MHz) δ 7.47 (d, 2H, J = 8.6 Hz, CH_{ar}), 7.22 (d, 2H, J = 8.6 Hz, CH_{ar}), 6.03 (s, 2H, CH_{ar}), 5.38 (s, 2H, CH_2), 4.53 (t, 2H, J = 6.7 Hz, CH_2), 3.91 (t, 2H, J = 5.7 Hz, CH_2), 3.04 (t, 2H, J = 6.7 Hz, CH_2), 2.91 (t, 2H, J = 5.7 Hz, CH_2), 2.52 (s, 6H, 2 CH_3), 2.00 (s, 6H, 2 CH_3). **^{13}C NMR** ($CDCl_3$, 125 MHz) δ 165.1 (CO), 157.9 (2 C_Q), 153.4 (CO), 151.6 (C_Q), 141.2 (2 C_Q), 131.8 (C_Q), 130.6 (2 CH_{ar}), 128.9 (2 C_Q), 128.6 (C_Q), 121.6 (2 CH_{ar}), 121.4 (2 CH_{ar}), 67.8 (CH_2), 66.6 (CH_2), 60.4 (CH_2), 41.7 (CH_2), 36.8 (CH_2), 14.9 (2 CH_3), 12.8 (2 CH_3). **HRMS** (ESI) calcd for $C_{26}H_{28}O_6N_2BF_2S_2$ [M–H]$^-$ 577.1460, found 577.1444.

4-(((2-(Phenyldisulfanyl)ethoxy)carbonyl)oxy)benzyl 5,5-difluoro-1,3,7,9-tetramethyl-5H-4λ4,5λ4-dipyrrolo[1,2-c:2',1'-f][1,3,2]diazaborinine-10-carboxylate **14**

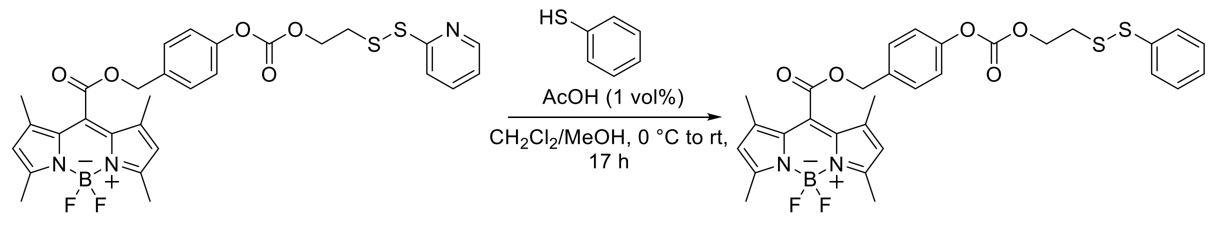

To activated disulfide **S-7** (10 mg, 0.016 mmol) in CH_2Cl_2 (1 mL) was added AcOH (1vol%) as catalyst. The solution was cooled to 0 °C and thiophenol (0.02 mmol, 2.2 mg) dissolved in MeOH (1 mL) was added. The reaction was allowed to warm to r.t. over 17 h. Upon completion of the reaction, the mixture was diluted with CH_2Cl_2 (20 mL) and washed with 1 M NaOH. The organic layer was dried with $MgSO_4$ and concentrated *in vacuo*. The residue was eluted from a column of silica gel with 20% of EtOAc in cyclohexane to afford BODIPY **14** (9.6 mg, 96%) as a red solid. **^{1}H NMR** ($CDCl_3$, 400 MHz) δ 7.56 (dd, 2H, J = 1.5, 7.7 Hz, 2 CH_{ar}), 7.47 (d, 2H, J = 8.6 Hz, 2 CH_{ar}), 7.34 (dd, 2H, J = 7.7 Hz, 2 CH_2), 7.23-7.28 (m, 1H, CH_{ar}), 7.20 (d, 2H, J = 8.6 Hz, 2 CH_{ar}), 6.03 (s, 2H, 2 CH_{ar}), 5.38(s, 2H, CH_2), 4.48 (t, 2H, J = 6.6 Hz, CH_2), 3.05 (t, 2H, J = 6.6 Hz, CH_2), 2.52 (s, 6H, 2 CH_3), 2.00 (s, 6H,2 CH_3). **^{13}C NMR** ($CDCl_3$, 100 MHz) δ 165.1 (CO), 157.9 (2 C_Q), 153.2 (CO), 151.6 (C_Q), 141.2 (2 C_Q), 136.8 (C_Q), 131.7 (C_Q), 130.6 (2 CH_{ar}), 129.3 (2 CH_{ar}), 128.9 (2 C_Q), 128.7 (C_Q), 128.2 (2 CH_{ar}), 127.5 (CH_{ar}), 121.6 (2 CH_{ar}), 121.4 (d, J =

3.2 Hz, 2 CH$_{ar}$), 67.8 (CH_2), 66.3 (CH_2), 36.7 (CH_2), 14.9 (t, J = 1.9 Hz, 2 CH_3), 12.8 (2 CH_3). **HRMS** (ESI) calcd for $C_{30}H_{30}O_5N_2BF_2S_2$ $[M+H]^+$ 661.1657, found: 6611.654.

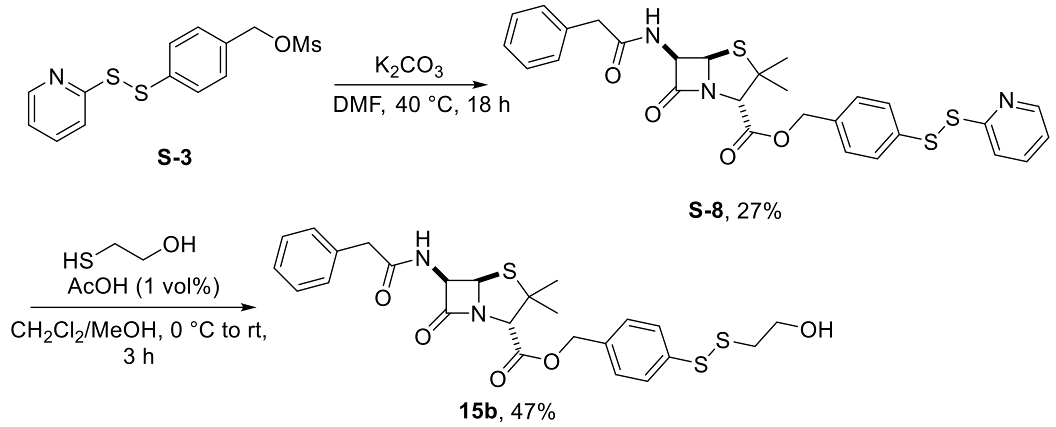

(2S,5R,6R)-4-(Pyridin-2-yldisulfanyl)benzyl 3,3-dimethyl-7-oxo-6-(2-phenylacetamido)-4-thia-1-azabicyclo[3.2.0]heptane-2-carboxylate **S-8**

To a solution of **S-3** (128 mg, 0.39 mmol) in dry DMF (800 μL) under argon atmosphere was added Penicilin G sodium salt (153 mg, 0.43 mmol). The resulting yellow solution was stirred at 40 °C for 18 h and then concentrated *in vacuo*. The residue was eluted from a column of silica gel with 25% to 33% EtOAc in cyclohexane to give **S-8** (60 mg, 27%) as a light-yellow oil. **^{1}H NMR** ($CDCl_3$, 400 MHz) δ 8.49-8.45 (m, 1H, CH$_{ar}$), 7.65-7.59 (m, 2H, CH$_{ar}$), 7.51 (d, 2H, J = 8.4 Hz, CH$_{ar}$), 7.38-7.23 (m, 7H, CH$_{ar}$), 7.12-7.09 (m, 1H, CH$_{ar}$), 6.11 (d, 1H, J = 9.0 Hz, NH), 5.63 (dd, 1H, J = 4.2, 9.0 Hz, CH), 5.47 (d, 1H, J = 4.2 Hz, CH), 5.13 (d, 1H, J = 12.3 Hz, CH_2a), 5.09 (d, 1H, J = 12.3 Hz, CH_2b), 4.37 (s, 1H, CH), 3.62 (s, 2H, CH_2), 1.39 (s, 3H, CH_3), 1.34 (s, 3H, CH_3). **^{13}C NMR** ($CDCl_3$, 100 MHz) δ 173.5 (CO), 170.4 (CO), 167.4 (CO), 159.3 (C$_Q$), 149.8 (CH$_{ar}$), 137.5 (CH$_{ar}$), 137.2 (C$_Q$), 133.9 (C$_Q$), 133.8 (C$_Q$), 129.7 (2 CH$_{ar}$), 129.5 (2 CH$_{ar}$), 129.2 (2 CH$_{ar}$), 127.8 (CH$_{ar}$), 127.5 (2 CH$_{ar}$), 121.2 (CH$_{ar}$), 119.7 (CH$_{ar}$), 70.3 (CH), 68.2 (CH), 66.9 (CH_2), 64.6 (C$_Q$), 59.0 (CH), 43.5 (CH_2), 32.2 (CH_3), 26.8 (CH_3). **HRMS** (ESI) m/z calcd. for $C_{28}H_{28}O_4N_3S_3$ $[M+H]^+$ 566.1236, found 566.1222.

(2S,5R,6R)-4-((2-Hydroxyethyl)disulfanyl)benzyl 3,3-dimethyl-7-oxo-6-(2-phenylacetamido)-4-thia-1-azabicyclo[3.2.0]heptane-2-carboxylate **15b**

To activated disulfide **S-8** (36 mg, 0.064 mmol) in CH_2Cl_2 (1 mL) was added AcOH (1vol%) as catalyst. The solution was cooled to 0 °C and 2-mercaptoethanol (0.076 mmol, 6 mg, 1.2 equiv.) dissolved in MeOH (1 mL) was added. The reaction was allowed to warm to rt over 3 h. Upon completion of the reaction, the mixture was diluted with CH_2Cl_2 (20 mL) and washed with 1 M NaOH. The organic layer was dried with $MgSO_4$ and concentrated *in vacuo*. The residue was eluted from a column of silica gel with 50% of EtOAc in cyclohexane to afford **15b** (16 mg, 47%) as a translucid oil. **^{1}H NMR** ($CDCl_3$, 400 MHz) δ 7.55 (d, 2H, J = 8.3 Hz, CH$_{ar}$), 7.38-7.25 (m, 7H, CH$_{ar}$), 6.10 (d, 1H, J = 6.7 Hz, NH), 5.63 (dd, 1H, J = 4.2, 9.0 Hz, CH), 5.48 (d, 1H, J = 4.2 Hz, CH), 5.15 (d, 1H, J = 12.5 Hz, CH_2a), 5.12 (d, 1H, J = 12.5 Hz, CH_2b), 4.39 (s, 1H, CH), 3.84 (t, 2H, J = 5.7 Hz, CH_2), 3.63 (s, 2H, CH_2), 2.89 (t, 2H, J = 5.7 Hz, CH_2), 1.40 (s, 3H, CH_3), 1.35 (s, 3H, CH_3). **^{13}C NMR** ($CDCl_3$, 100 MHz) δ 173.5 (CO), 170.5 (CO), 167.5 (CO), 138.2 (C$_Q$), 133.9 (C$_Q$), 133.7 (C$_Q$), 129.7 (2 CH$_{ar}$), 129.6 (2 CH$_{ar}$), 129.3 (2 CH$_{ar}$), 127.8 (3 CH$_{ar}$), 70.4 (CH), 68.3

(CH), 67.0 (CH_2), 64.7 (C_Q), 60.1 (CH_2), 59.0 (CH), 43.5 (CH_2), 41.4 (CH_2), 32.2 (CH_3), 26.8 (CH_3). **HRMS** (ESI) *m/z* calcd. for $C_{25}H_{29}O_5N_2S_3$ $[M+H]^+$ 533.1233, found 533.1218.

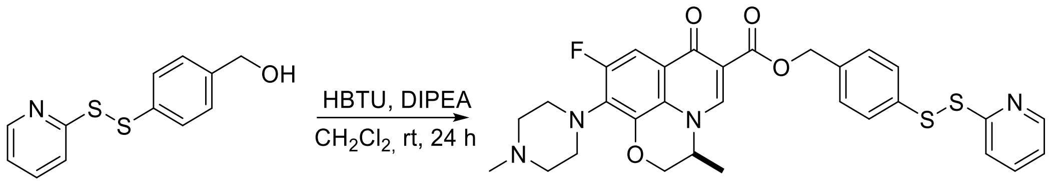

S-2 S-9, 58%

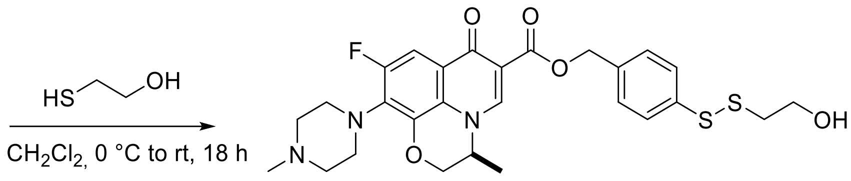

16b, 45%

4-(Pyridin-2-yldisulfanyl)benzyl (S)-9-fluoro-3-methyl-10-(4-methylpiperazin-1-yl)-7-oxo-2,3-dihydro-7H-[1,4]oxazino[2,3,4-ij]quinoline-6-carboxylate **S-9**

To a solution of alcohol **S-2** (80 mg, 0.32 mmol) in anhydrous DCM (12 mL) were added levofloxacin (116 mg, 0.32 mmol), HBTU (182 mg, 0.48 mmol) and DIPEA (217 µL, 1.28 mmol). The resulting mixture was stirred for 24 h then water was added. The resulting mixture was extracted with CH_2Cl_2, washed with saturated $NaHCO_3$ solution. The organic phase was then dried with $MgSO_4$ and concentrated *in vacuo*. The residue was eluted from a column of silica gel with 5 to 10% of MeOH in CH_2Cl_2 to give **S-9** (111 mg, 58%) as a white foam. **^{1}H NMR** ($CDCl_3$, 400 MHz) δ 8.45-8.43 (m, 1H, CH_{ar}), 8.12 (s, 1H, CH_{ar}), 7,62-7,56 (m, 2H, 2 CH_{ar}), 7.51 (d, 1H, J = 8.3 Hz, 2 CH_{ar}), 7.44 (d, 1H, J = 10.9 Hz, CH_{ar}), 7.53 (d, 1H, J = 8.2 Hz, 2 CH_{ar}), 7.43 (d, 1H, J = 8.3 Hz, 2 CH_{ar}), 7.09-7.05 (m, 1H, CH_{ar}), 5.29 (d, 1H, J = 12.7 Hz, C*H*H), 5.24 (d, 1H, J = 12.7 Hz, CH*H*), 4.37 (dd, 1H, J = 11.5, 2.6 Hz, C*H*H), 4.26 (dd, 1H, J = 11.5, 2.2 Hz, C*H*H), 4.13-4.07 (m, 1H, CH), 3.36-3.29 (m, 4H, 2 CH_2), 2.60-2.50 (m, 4H, 2 CH_2), 2.36 (s, CH_3), 1.41 (d, J = 6.7 Hz, CH_3).). **^{13}C NMR** ($CDCl_3$, 100 MHz) δ 172.7 (CO), 165.5 (CO), 159.5 (C_Q), 155.8 (d, 1C, J = 247.3 Hz, C_Q), 149.7 (CH_{ar}), 145.6 (CH_{ar}), 139.7 (d, 1C, J = 6.5 Hz, C_Q), 137.5 (C_Q), 137.4 (CH_{ar}), 135.8 (C_Q), 131.8 (d, 1C, J = 14.5 Hz, C_Q), 129.2 (2 CH_{ar}), 127.7 (C_Q), 127.6 (2 CH_{ar}), 123.3 (d, 1C, J = 7.8 Hz, C_Q), 121.1 (CH_{ar}), 119.8 (CH_{ar}) 109.2 (C_Q), 105.4 (d, 1C, $J_{C,F}$ = 24.1 Hz, CH_{ar}), 68.1 (CH_2), 65.8 (CH_2), 55.8 (2 CH_2), 54.8 (CH), 50.6 (d, 1C, $J_{C,F}$ = 3.8 Hz, 2 CH_2), 46.4 (CH_3), 18.3 (CH_3). **HRMS** (ESI) *m/z* calcd. for $C_{30}H_{30}O_4N_4FS_2$ $[M+H]^+$ 593.1687, found 593.1684.

4-((2-Hydroxyethyl)disulfanyl)benzyl (S)-9-fluoro-3-methyl-10-(4-methylpiperazin-1-yl)-7-oxo-2,3-dihydro-7H-[1,4]oxazino[2,3,4-ij]quinoline-6-carboxylate **16b**

To activated disulfide **S-9** (60 mg, 0.10 mmol) in CH_2Cl_2 (1 mL) at 0 °C was added 2-mercaptoethanol (9.4 mg, 0.12 mmol). The reaction was allowed to warm to rt and stirred for 18 h. Upon completion of the reaction, the mixture concentrated *in vacuo*. The residue was eluted from a column of silica gel with 7.5 to 15% of MeOH in CH_2Cl_2 to afford **16b** (25 mg, 45%) as a white foam. **^{1}H NMR** ($CDCl_3$, 400 MHz) δ 8.19 (s, 1H, CH_{ar}), 7.53 (d, 1H, J = 8.2 Hz, 2 CH_{ar}), 7.49 (d, 1H, J = 12.7 Hz, CH_{ar}), 7.53 (d, 1H, J = 8.2 Hz, 2 CH_{ar}), 7.46 (d, 1H, J = 8.2 Hz, 2 CH_{ar}), 5.32 (d, 1H, J = 12.7 Hz, C*H*H), 5.27 (d, 1H, J = 12.7 Hz, CH*H*), 4.37 (dd, 1H, J = 11.3, 2.0 Hz, C*H*H), 4.29 (dd, 1H, J = 11.3, 2.7 Hz, C*H*H), 4.22-4.14 (m, 1H, CH),

3.83 (t, J = 5.9 Hz, CH_2), 3.40-3.26 (m, 4H, 2 CH_2), 2.88 (t, J = 5.9 Hz, CH_2), 2.60-2.51 (m, 4H, 2 CH_2), 2.36 (s, CH_3), 1.45 (d, J = 6.8 Hz, CH_3). **^{13}C NMR** ($CDCl_3$, 100 MHz) δ 172.9 (CO), 165.6 (CO), 155.8 (d, 1C, J = 247.4 Hz, C_Q), 145.6 (CH_{ar}), 139.7 (d, 1C, J = 6.6 Hz, C_Q), 137.2 (C_Q), 135.5 (C_Q), 131.8 (d, 1C, J = 14.5 Hz, C_Q), 129.2 (2 CH_{ar}), 128.0 (2 CH_{ar}), 127.7 (C_Q), 123.4 (d, 1C, J = 8.4 Hz, C_Q), 109.2 (C_Q), 105.5 (d, 1C, $J_{C,F}$ = 24.1 Hz, CH_{ar}), 68.2 (CH_2), 65.9 (CH_2), 60.10 (CH_2), 55.8 (2 CH_2), 54.9 (CH), 50.5 (d, 1C, $J_{C,F}$ = 4.2 Hz, 2 CH_2), 46.4 (CH_3), 41.5 (CH_2), 18.3 (CH_3). **HRMS** (ESI) *m/z* calcd. for $C_{27}H_{31}O_5N_3FS_2$ $[M+H]^+$ 560.1684, found 560.1676.

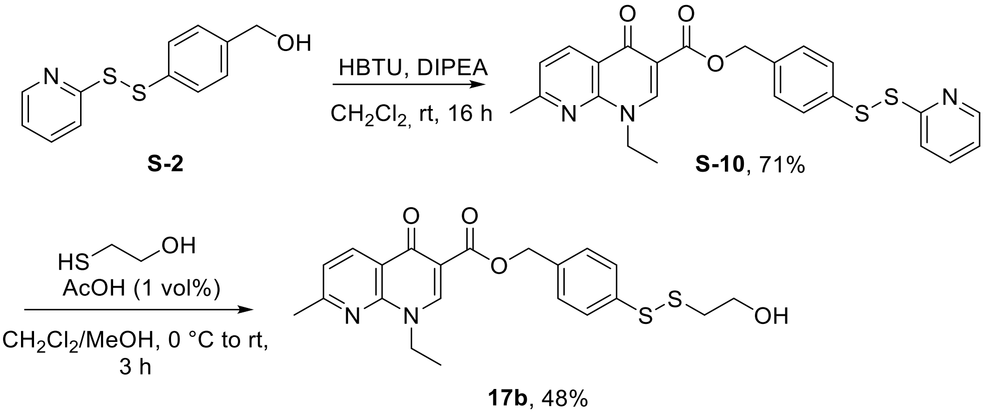

4-(Pyridin-2-yldisulfanyl)benzyl 1-ethyl-7-methyl-4-oxo-1,4-dihydro-1,8-naphthyridine-3- carboxylate **S-10**

To a solution of alcohol **S-2** (50 mg, 0.2 mmol) in anhydrous DCM (7.5 mL) were added Nalidixic acid (47 mg, 0.2 mmol), HBTU (114 mg, 0.3 mmol) and DIPEA (136 µL, 0.8 mmol). The resulting mixture was stirred for the night and then washed with water, extracted with CH_2Cl_2, washed with a saturated $NaHCO_3$ solution, dried with $MgSO_4$ and concentrated *in vacuo*. The residue was eluted from a column of silica gel with 33 to 66% of AcOEt in cyclohexane to give **S-10** (65.8 mg, 71%) as a light-yellow oil. **^{1}H NMR** ($CDCl_3$, 400 MHz) δ 8.62 (d, 1H, J = 8.1 Hz, CH_{ar}), 8.59 (s, 1H, CH_{ar}), 8.48-8.45 (m, 1H, CH_{ar}), 7.65-7.58 (m, 2H, 2 CH_{ar}), 7.52 (d, 2H, J = 8.5 Hz, 2 CH_{ar}), 7.46 (d, 2H, J = 8.5 Hz, 2 CH_{ar}), 7.23 (d, 1H, J = 8.1 Hz, CH_{ar}), 7.11-7.06 (m, 1H, CH_{ar}), 5.34 (s, 2H, CH_2), 4.45 (q, 2H, J = 7.2 Hz, CH_2), 2.64 (s, 3H, CH_3), 1.46 (t, 3H, J = 7.2 Hz, CH_3). **^{13}C NMR** ($CDCl_3$, 100 MHz) δ 174.7 (CO), 165.5 (CO), 162.8 (C_Q), 159.7 (C_Q), 149.7 (CH_{ar}), 149.0 (CH_{ar}), 148.7 (C_Q), 137.4 (CH_{ar}), 136.9 (CH_{ar}), 135.8 (C_Q), 135.7 (C_Q), 128.9 (2 CH_{ar}), 127.6 (2 CH_{ar}), 121.7 (C_Q), 121.3 (CH_{ar}), 121.0 (CH_{ar}), 119.7 (CH_{ar}), 111.6 (C_Q), 65.9 (CH_2), 46.8 (CH_2), 25.2 (CH_3), 15.3 (CH_3). **HRMS** (ESI) *m/z* calcd. for $C_{24}H_{22}O_3N_3S_2$ $[M+H]^+$ 464.10971, found 464.10894.

4-((2-Hydroxyethyl)disulfanyl)benzyl 1-ethyl-7-methyl-4-oxo-1,4-dihydro-1,8-naphthyridine3-carboxylate **17b**

To activated disulfide **S-10** (38 mg, 0.081 mmol) in CH_2Cl_2 (1 mL) was added AcOH (1vol%) as catalyst. The solution was cooled to 0 °C and 2-mercaptoethanol (7.6 mg, 0.097 mmol) dissolved in MeOH (1 mL) was added. The reaction was allowed to warm to rt over 17 h. Upon completion of the reaction, the mixture was diluted with CH_2Cl_2 (20 mL) and washed with 1 M NaOH. The organic layer was dried with $MgSO_4$ and concentrated *in vacuo*. The residue was eluted from a column of silica gel with 80% of EtOAc in cyclohexane to afford **17b** (18 mg, 48%) as a white amorphous solid. **^{1}H NMR** ($CDCl_3$, 400

MHz) δ 8.63 (s, 1H, CH_{ar}), 8.61 (d, 1H, *J* = 8.1 Hz, CH_{ar}), 7.53 (d, 2H, *J* = 8.2 Hz, 2 CH_{ar}), 7.47 (d, 2H, *J* = 8.2 Hz, 2 CH_{ar}), 7.23 (d, 2H, *J* = 8.1 Hz, CH_{ar}), 5.34 (s, 2H, CH_2), 4.46 (q, 2H, *J* = 7.1 Hz, CH_2), 3.83 (t, 2H, *J* = 5.8 Hz, CH_2), 2.88 (t, 2H, *J* = 5.8 Hz, CH_2), 2.65 (s, 3H, CH_3), 1.47 (t, 3H, *J* = 7.1 Hz, CH_3). **^{13}C NMR** ($CDCl_3$, 100 MHz) δ 175.0 (CO), 165.3 (CO), 163.0 (C_Q), 149.0 (C_Q), 148.6 (C_Q), 136.9 (CH_{ar}), 136.8 (CH_{ar}), 135.4 (C_Q), 129.0 (2 CH_{ar}), 128.0 (2 CH_{ar}), 121.4 (CH_{ar}), 121.4 (C_Q), 111.2 (C_Q), 65.9 (CH_2), 60.0 (CH_2), 46.8 (CH_2), 41.2 (CH_2), 25.1 (CH_3), 15.2 (CH_3). **HRMS** (ESI) *m/z* calcd. for $C_{21}H_{23}O_4N_2S_2$ $[M+H]^+$ 431.10938, found 431.10898.

3. NMR spectra

6: ^{1}H NMR ($CDCl_3$, 400 MHz)

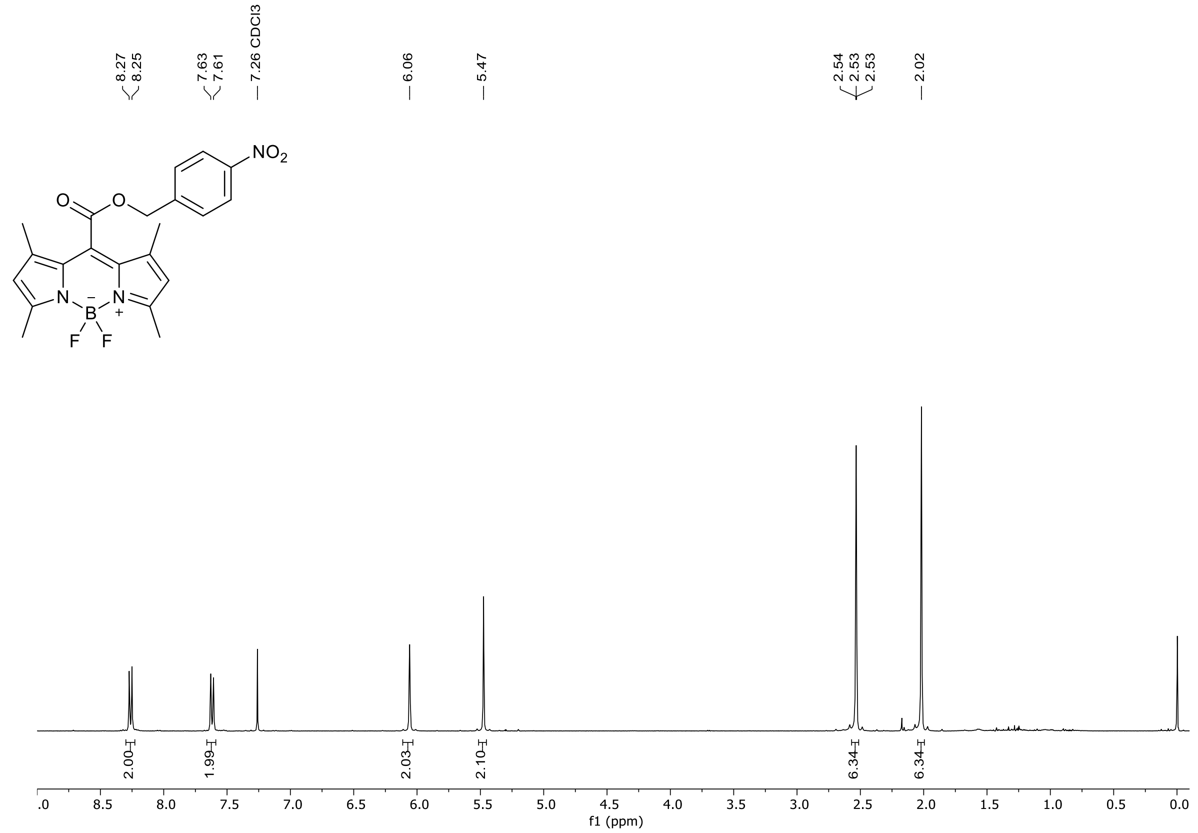

6: ^{13}C NMR ($CDCl_3$, 100 MHz)

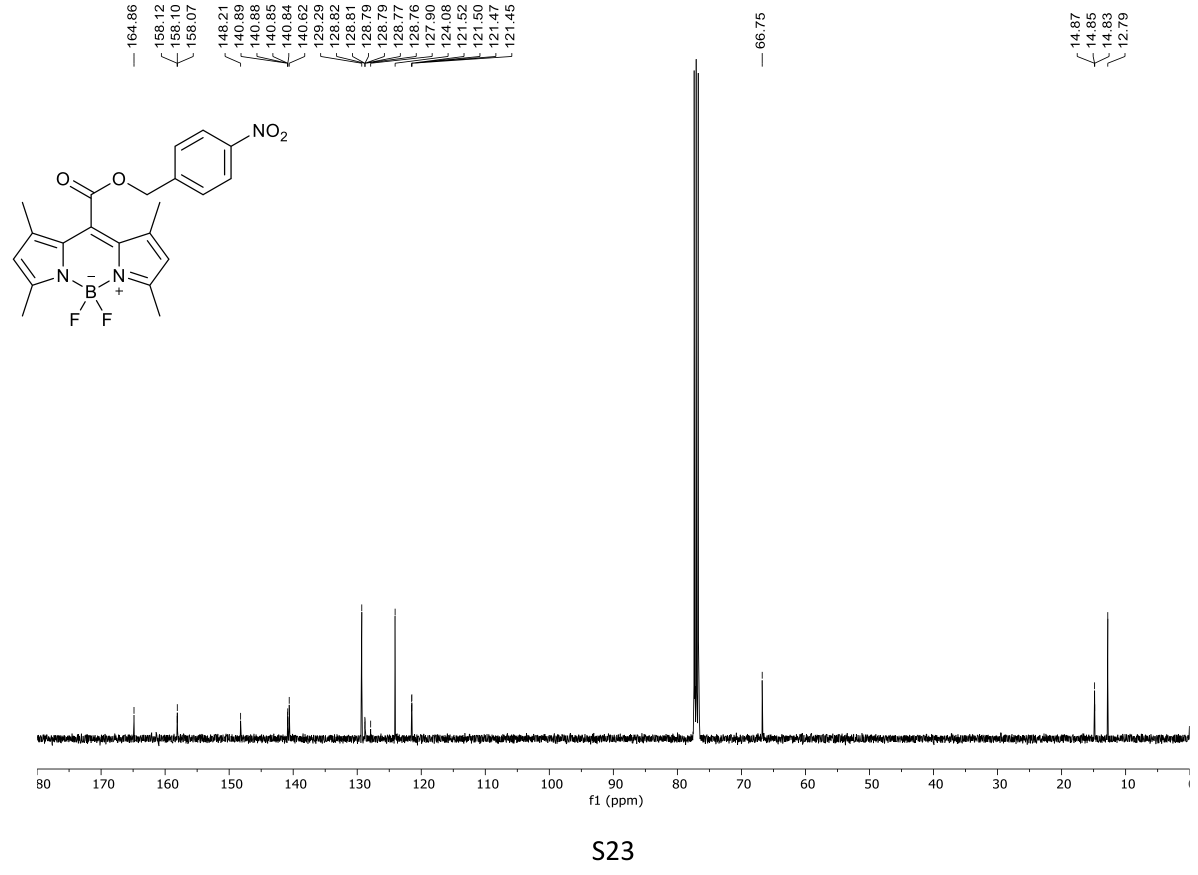

7: ^{1}H NMR ($CDCl_3$, 500 MHz)

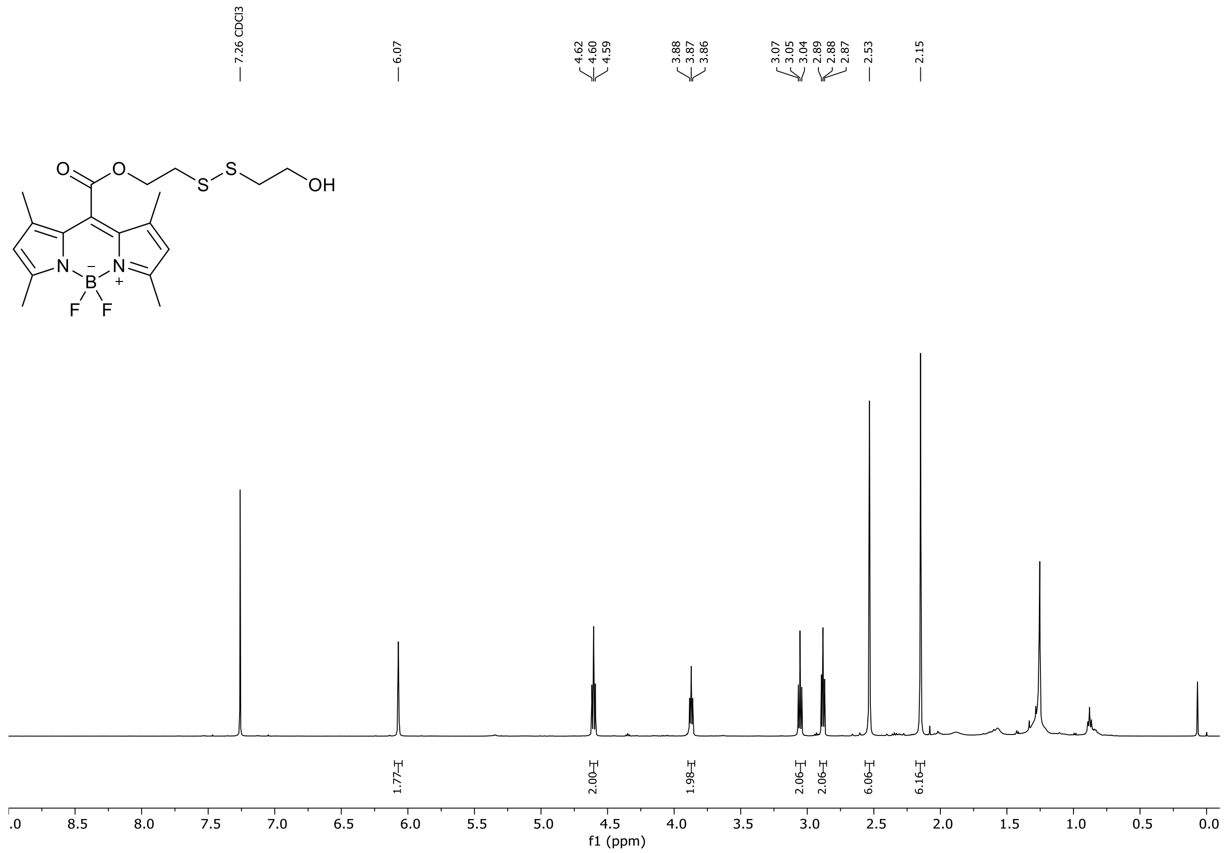

7: ^{13}C NMR ($CDCl_3$, 125 MHz)

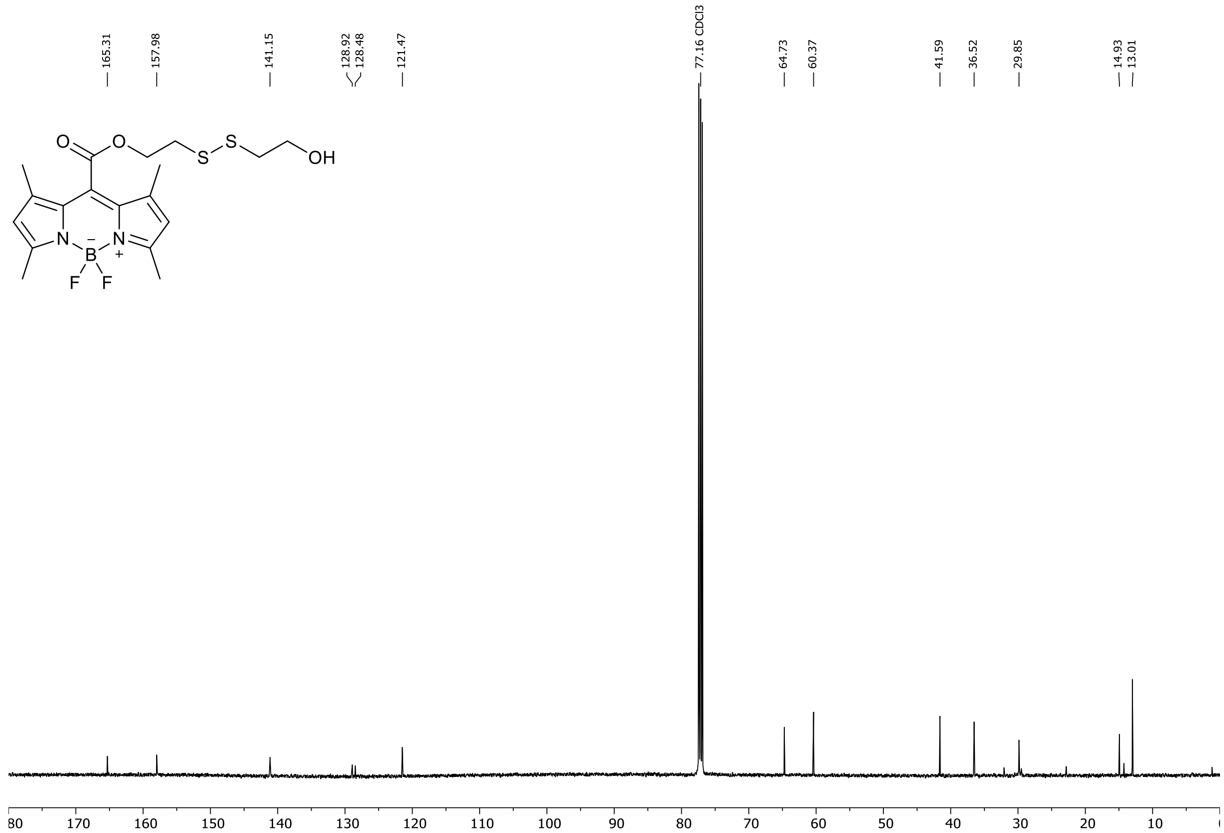

S-3: ^{1}H NMR ($CDCl_3$, 400 MHz)

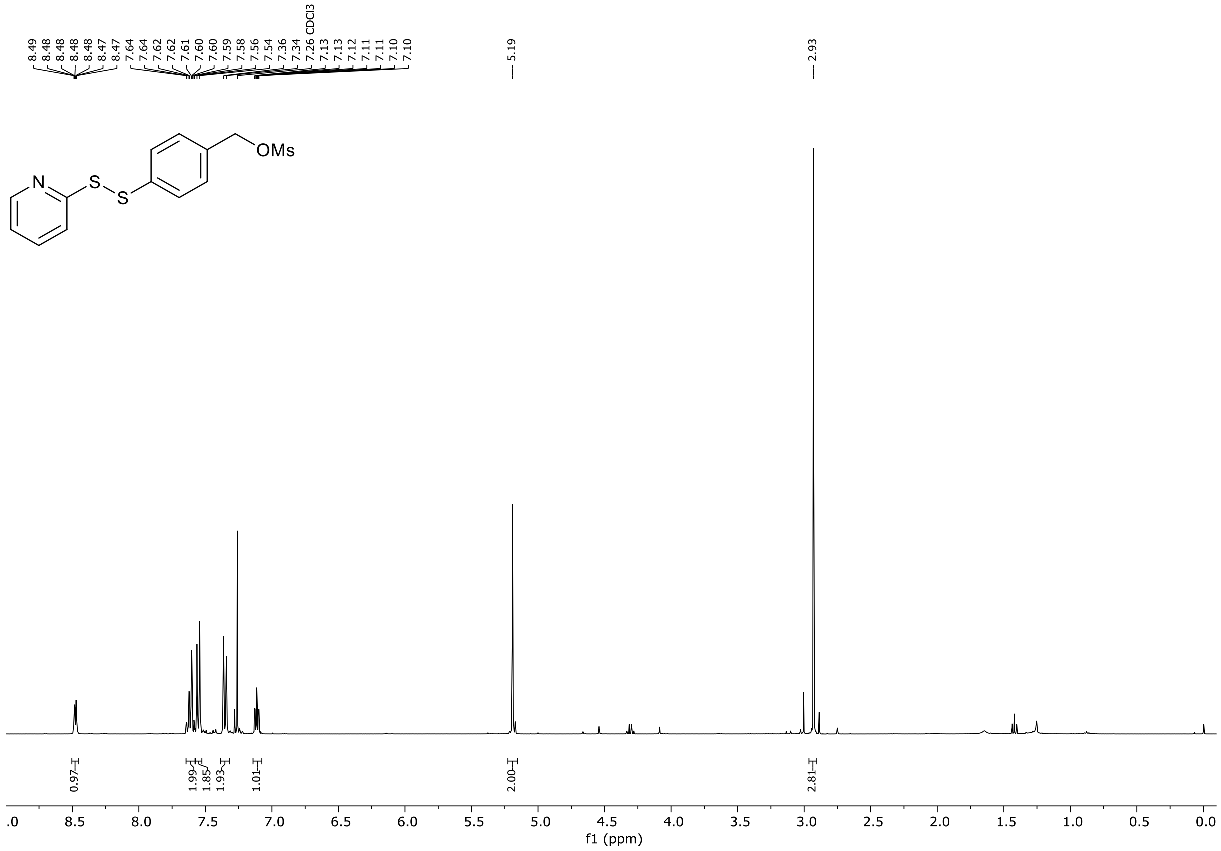

S-3: ^{13}C NMR ($CDCl_3$, 100 MHz)

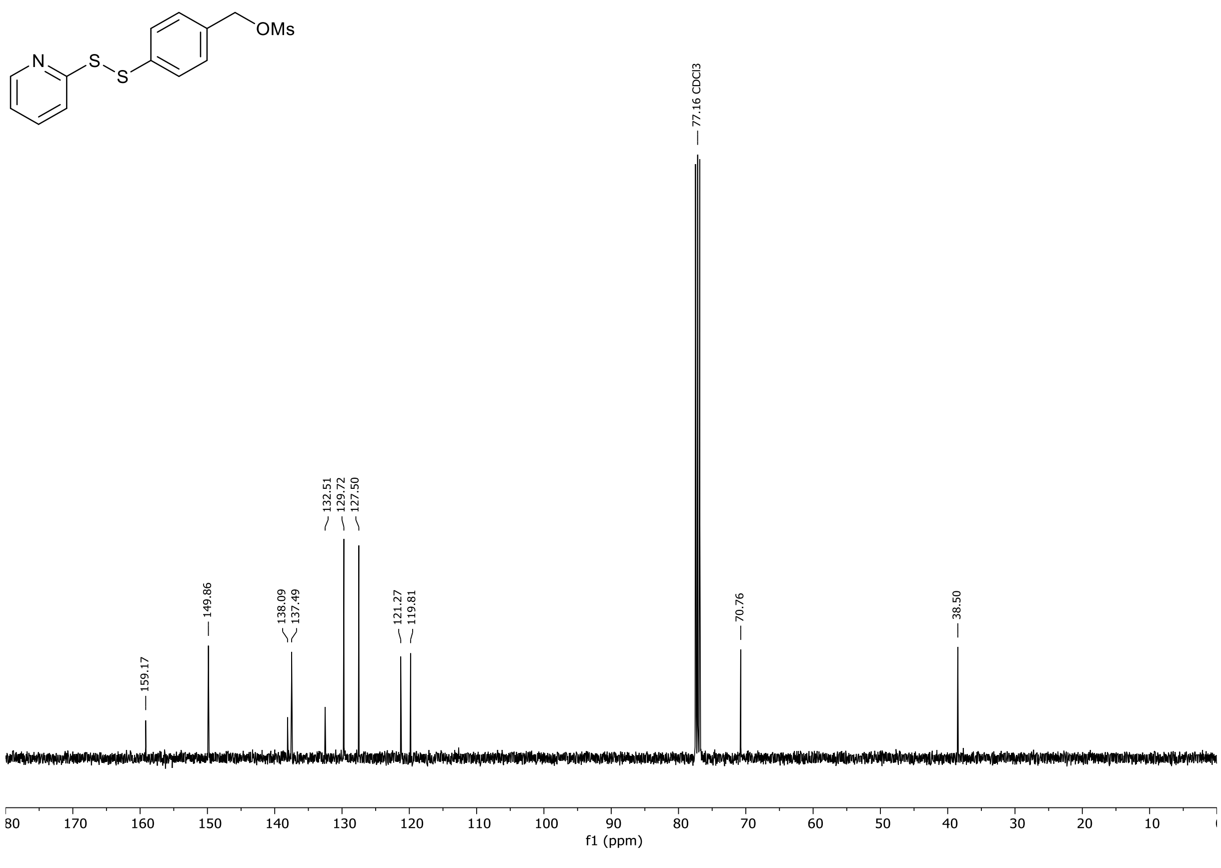

S-4: ^{1}H NMR ($CDCl_3$, 400 MHz)

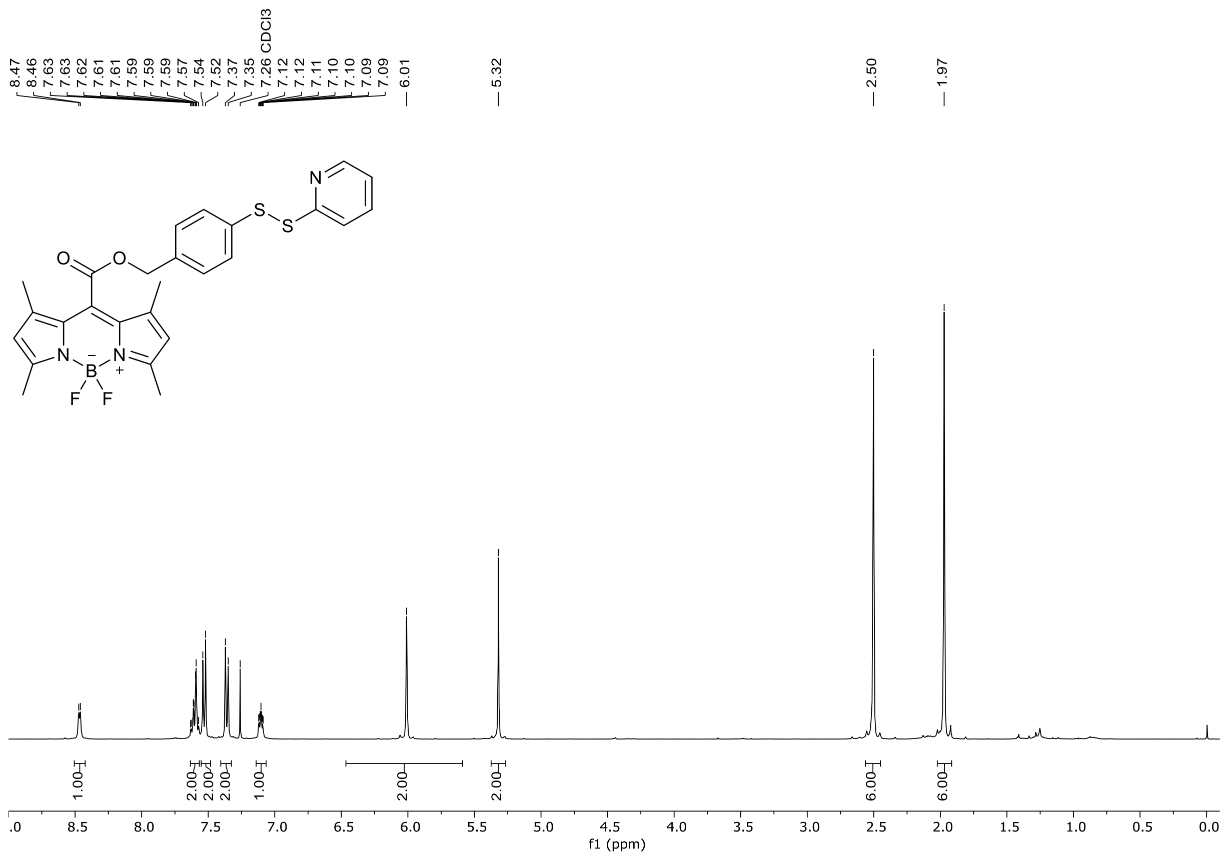

S-4: ^{13}C NMR ($CDCl_3$, 100 MHz)

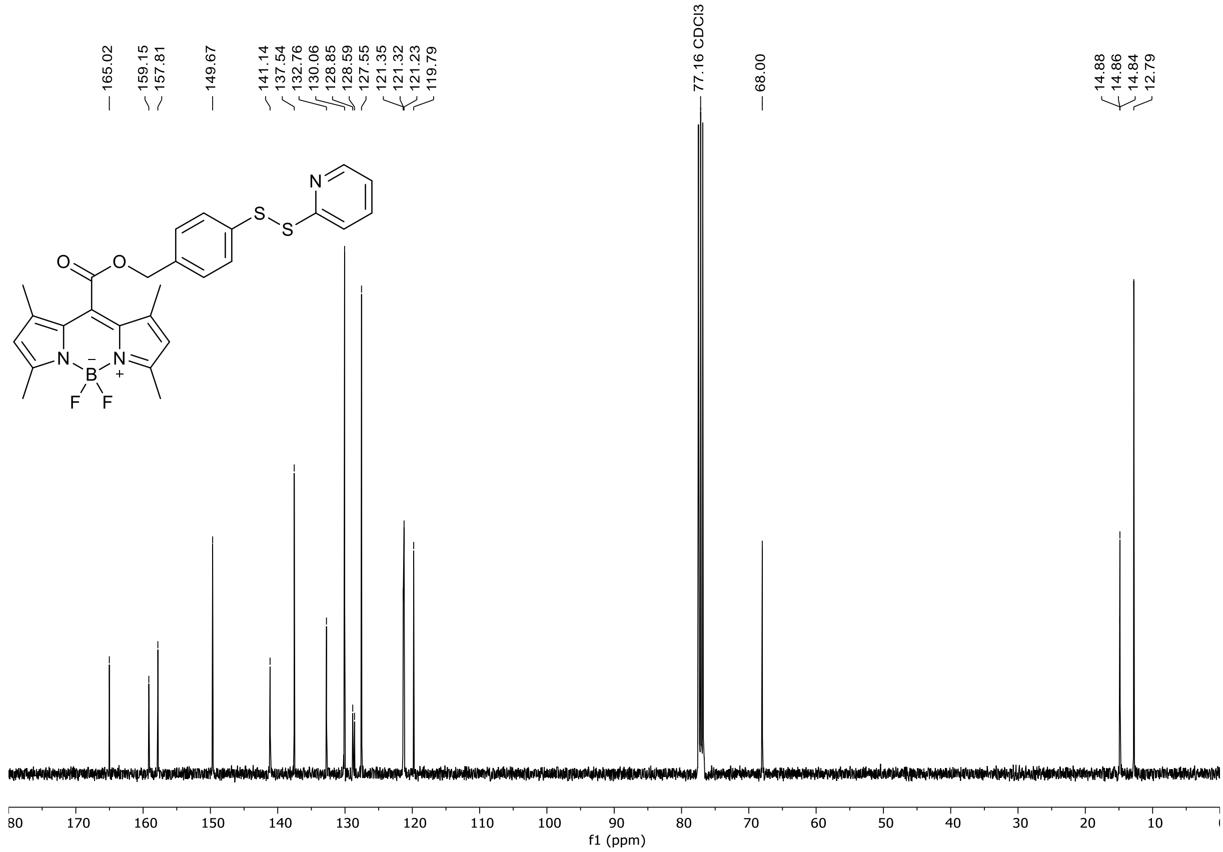

8: ^{1}H NMR ($CDCl_3$, 400 MHz)

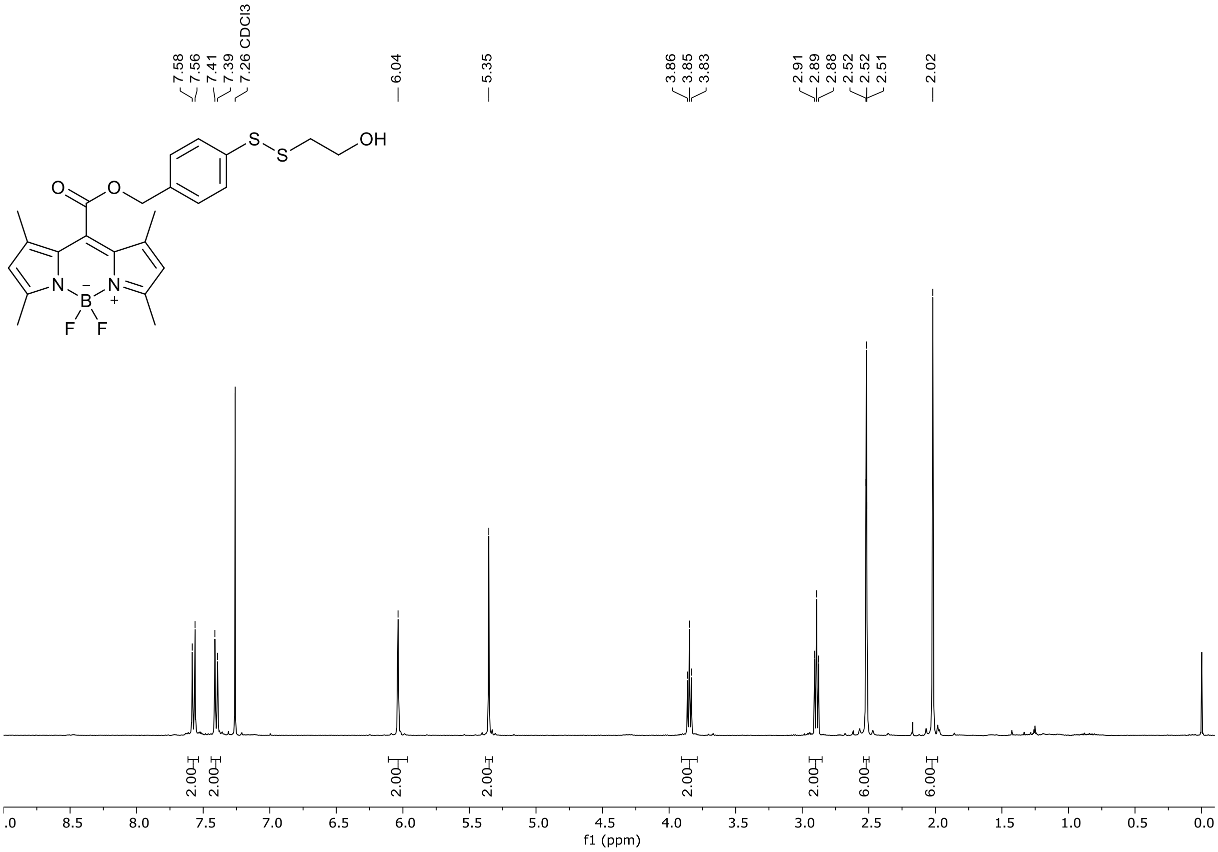

8: ^{13}C NMR ($CDCl_3$, 100 MHz)

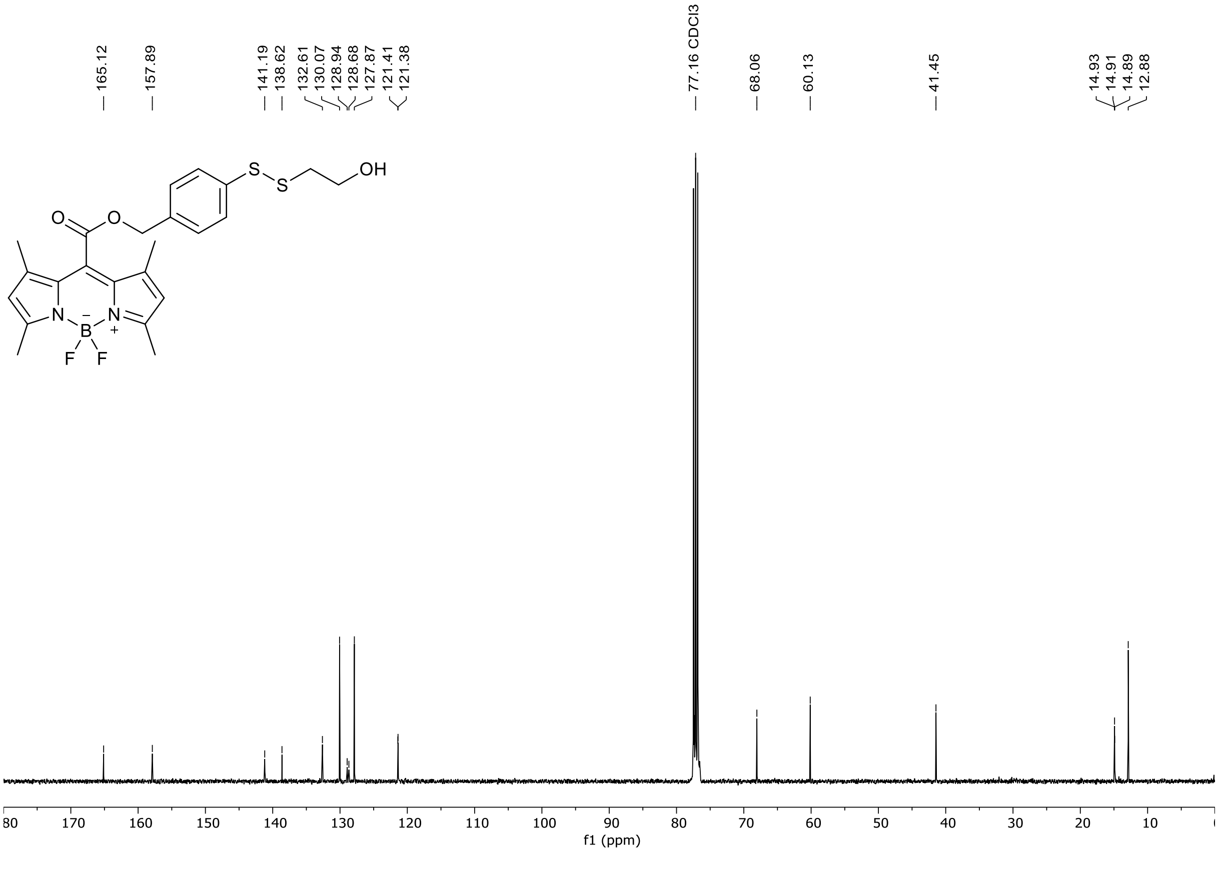

9: ^{1}H NMR ($CDCl_3$, 400 MHz)

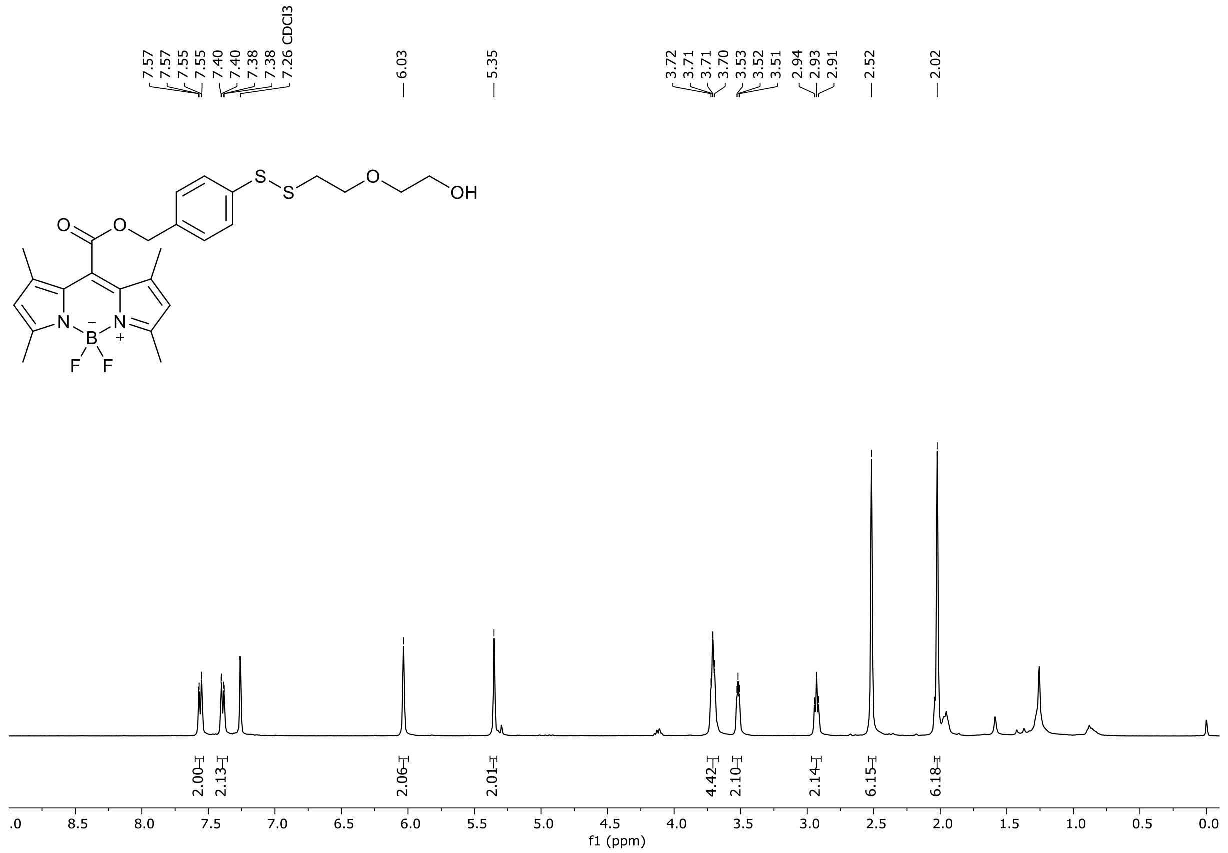

9: ^{13}C NMR ($CDCl_3$, 100 MHz)

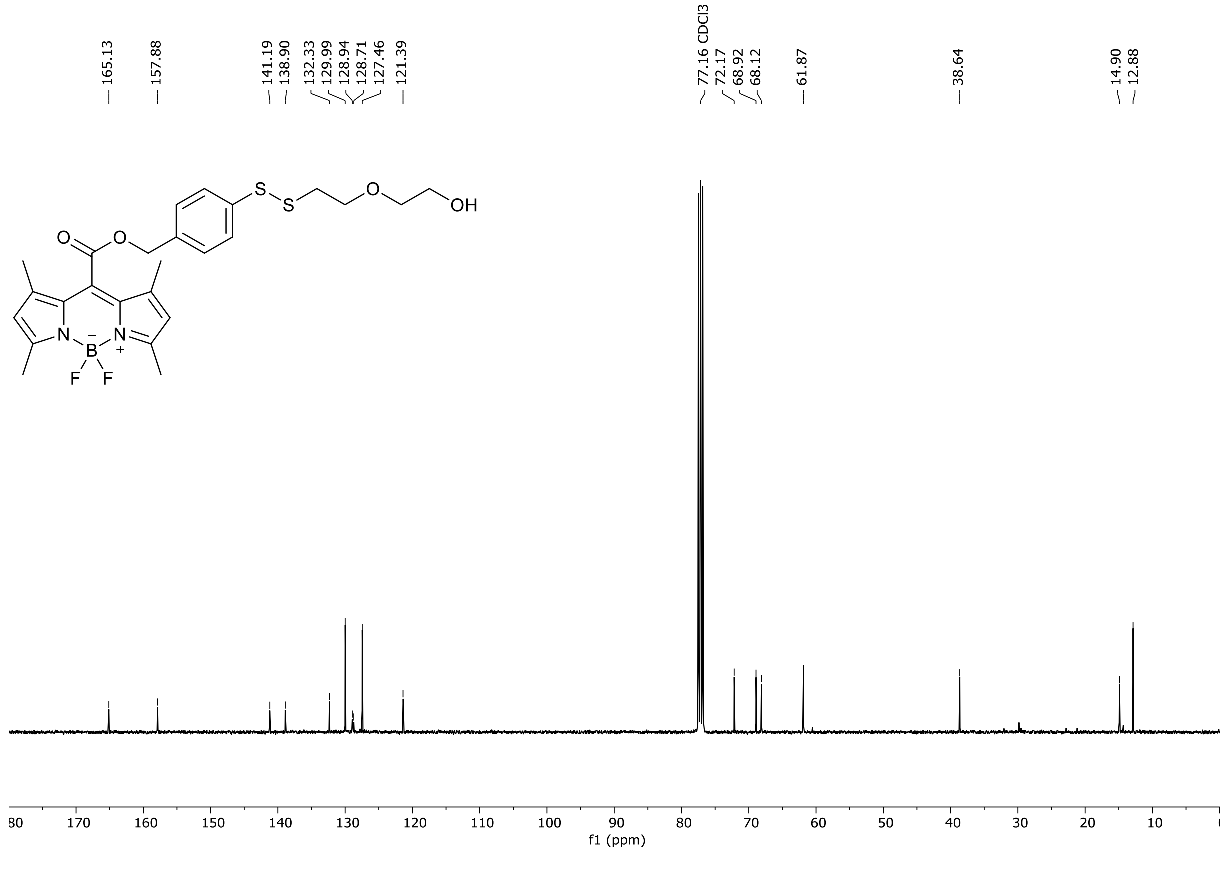

10: ^{1}H NMR ($CDCl_3$, 500 MHz)

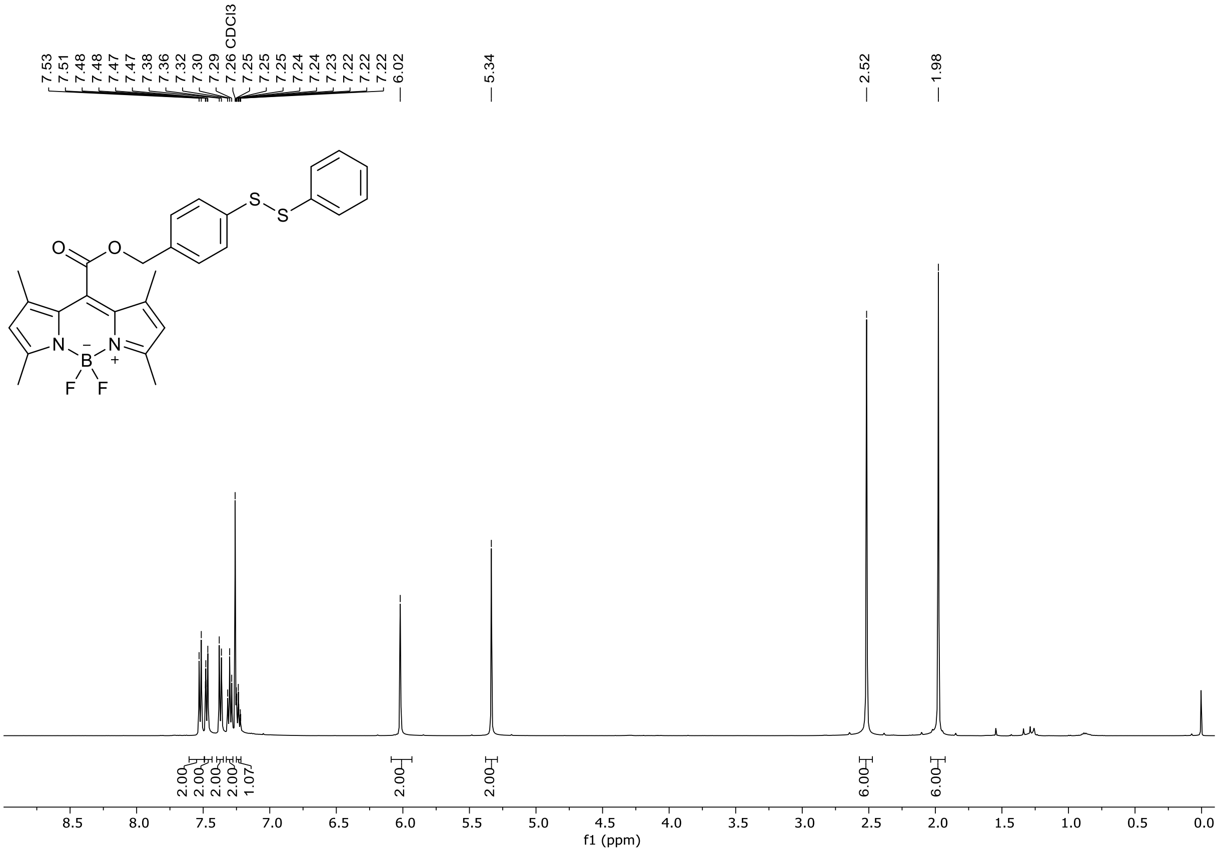

10: ^{13}C NMR ($CDCl_3$, 125 MHz)

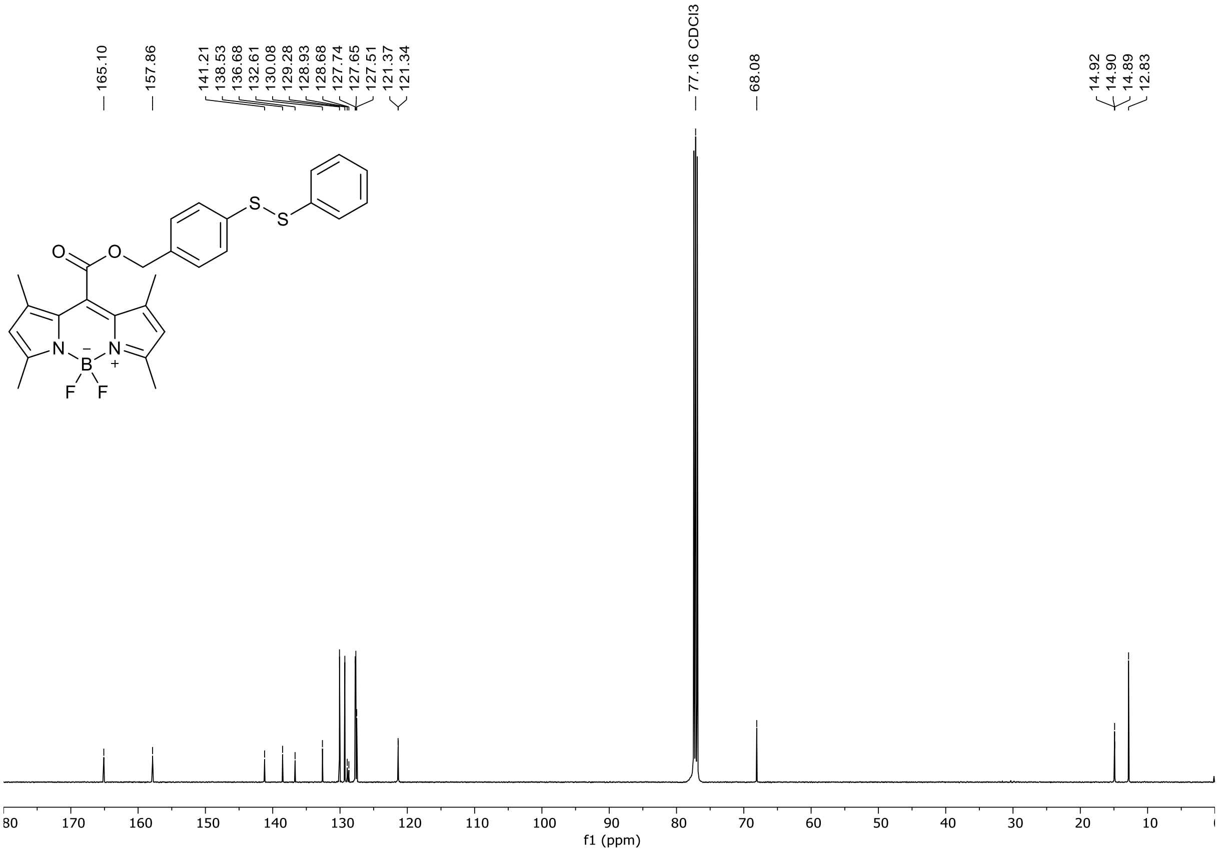

11: ^{1}H NMR ($CDCl_3$, 500 MHz)

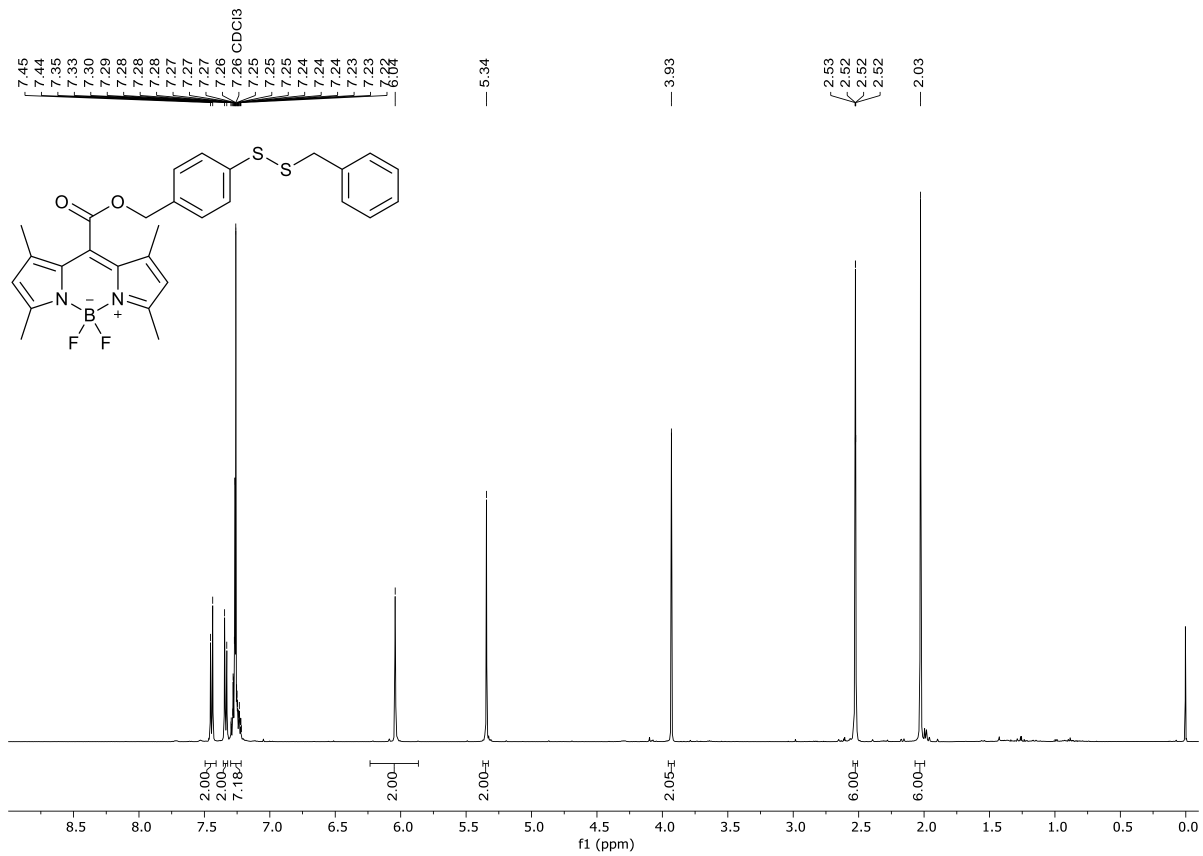

11: ^{13}C NMR ($CDCl_3$, 125 MHz)

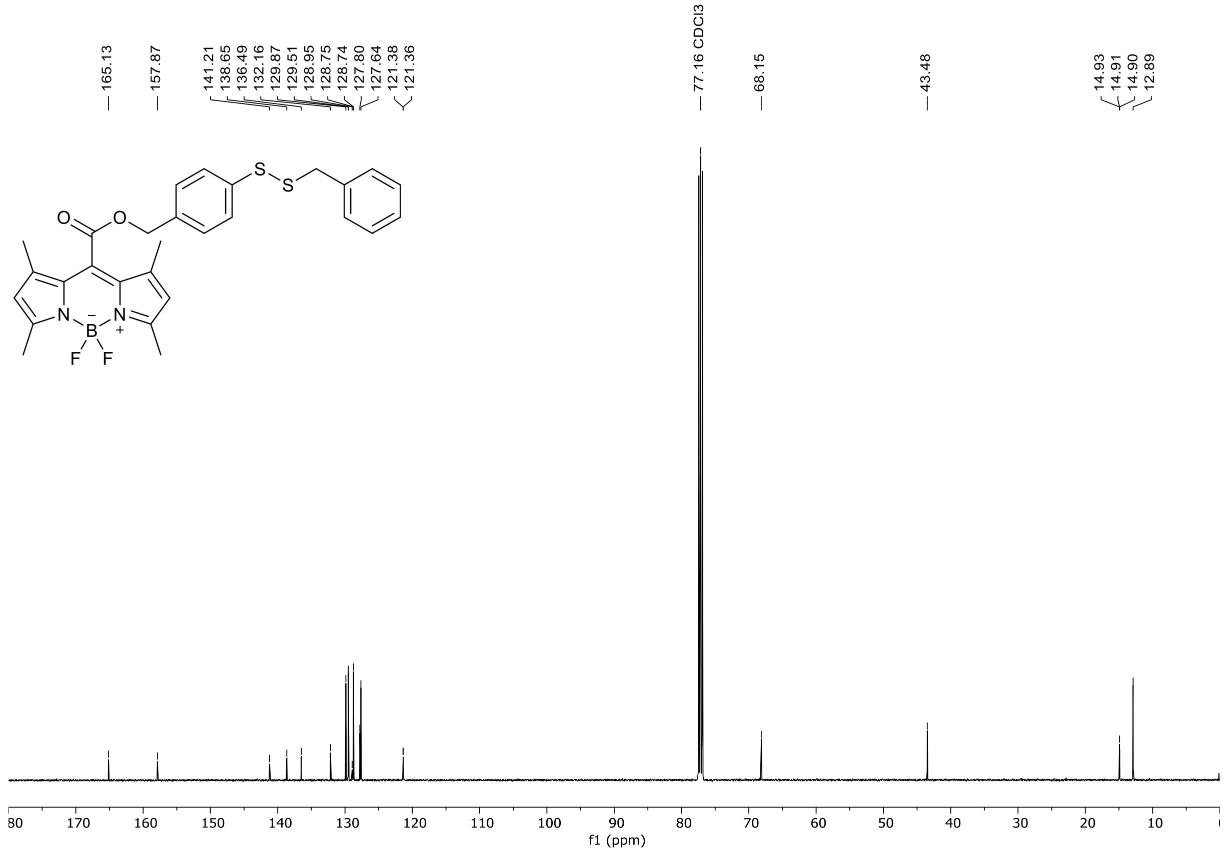

12: ^{1}H NMR ($CDCl_3$, 500 MHz)

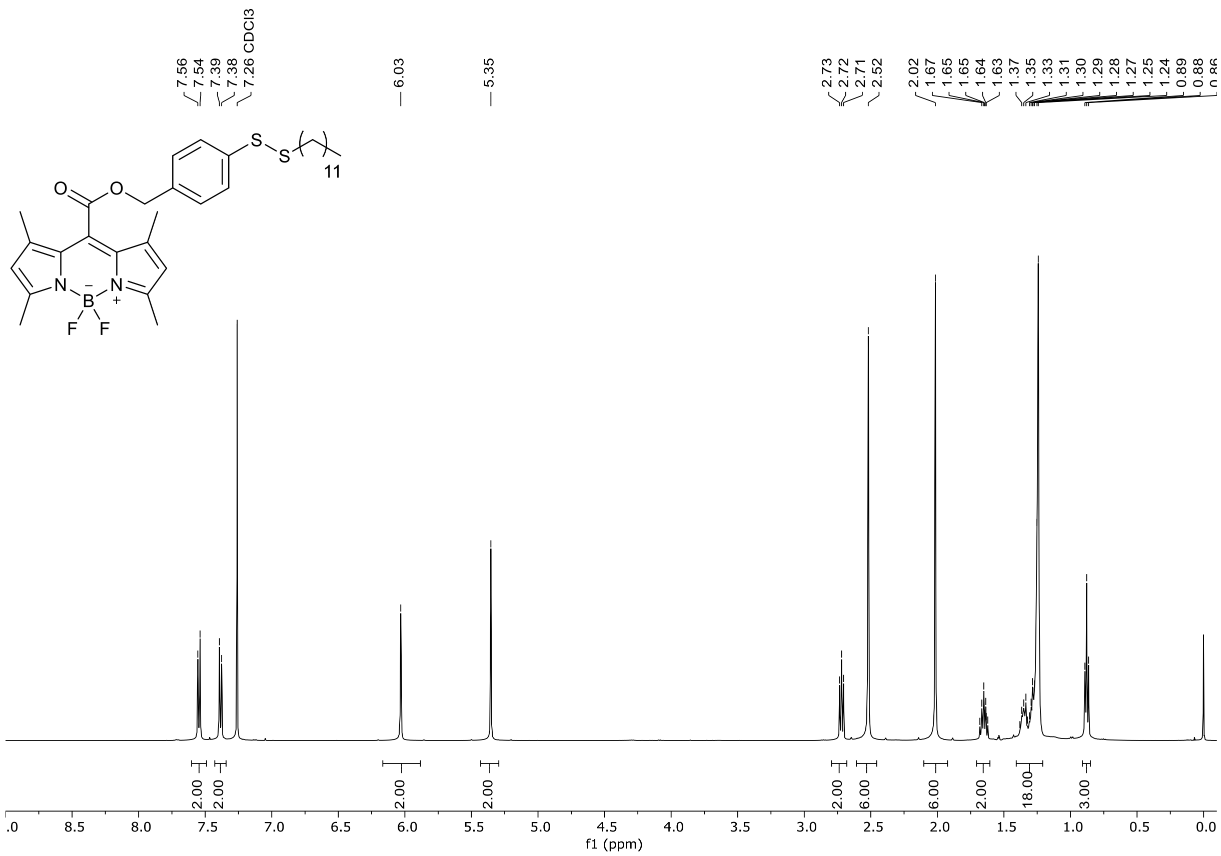

12: ^{13}C NMR ($CDCl_3$, 125 MHz)

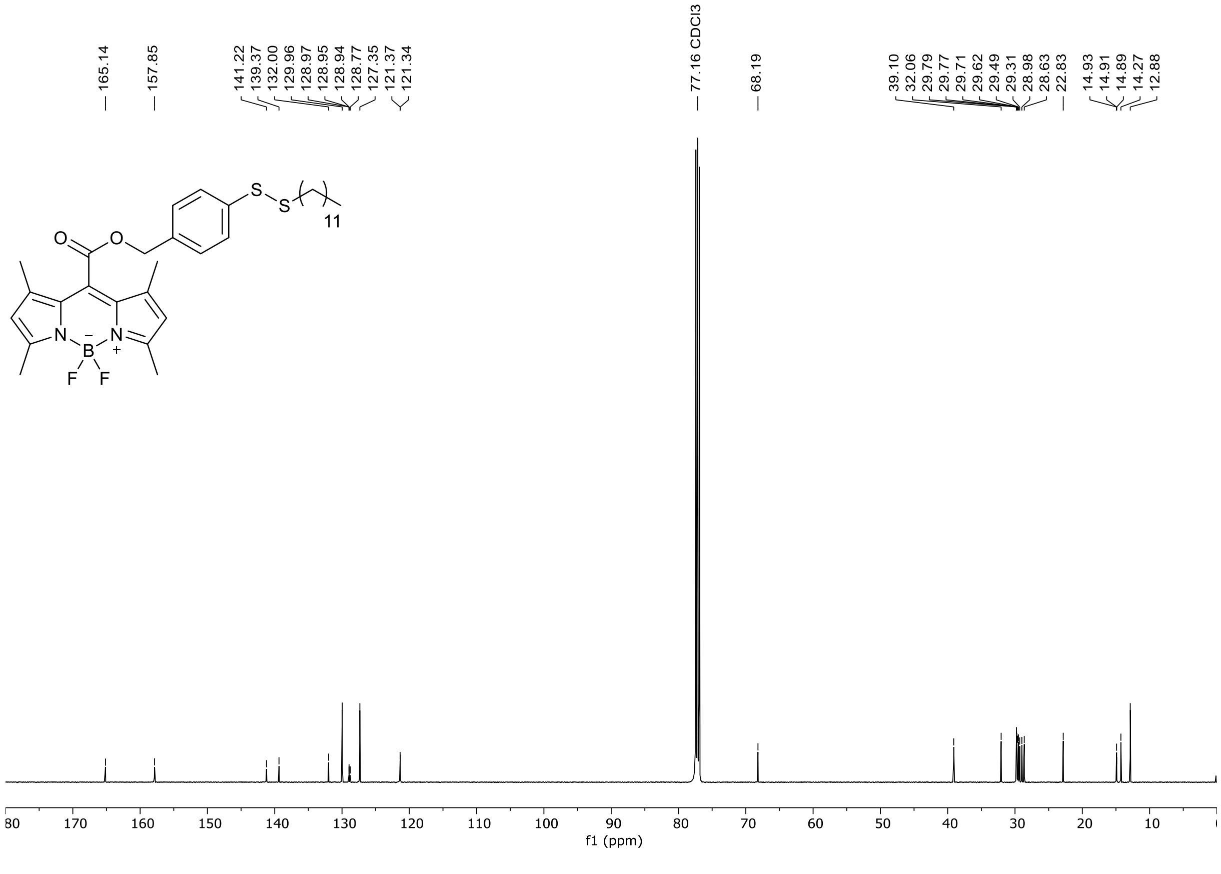

S-6: ^{1}H NMR ($CDCl_3$, 400 MHz)

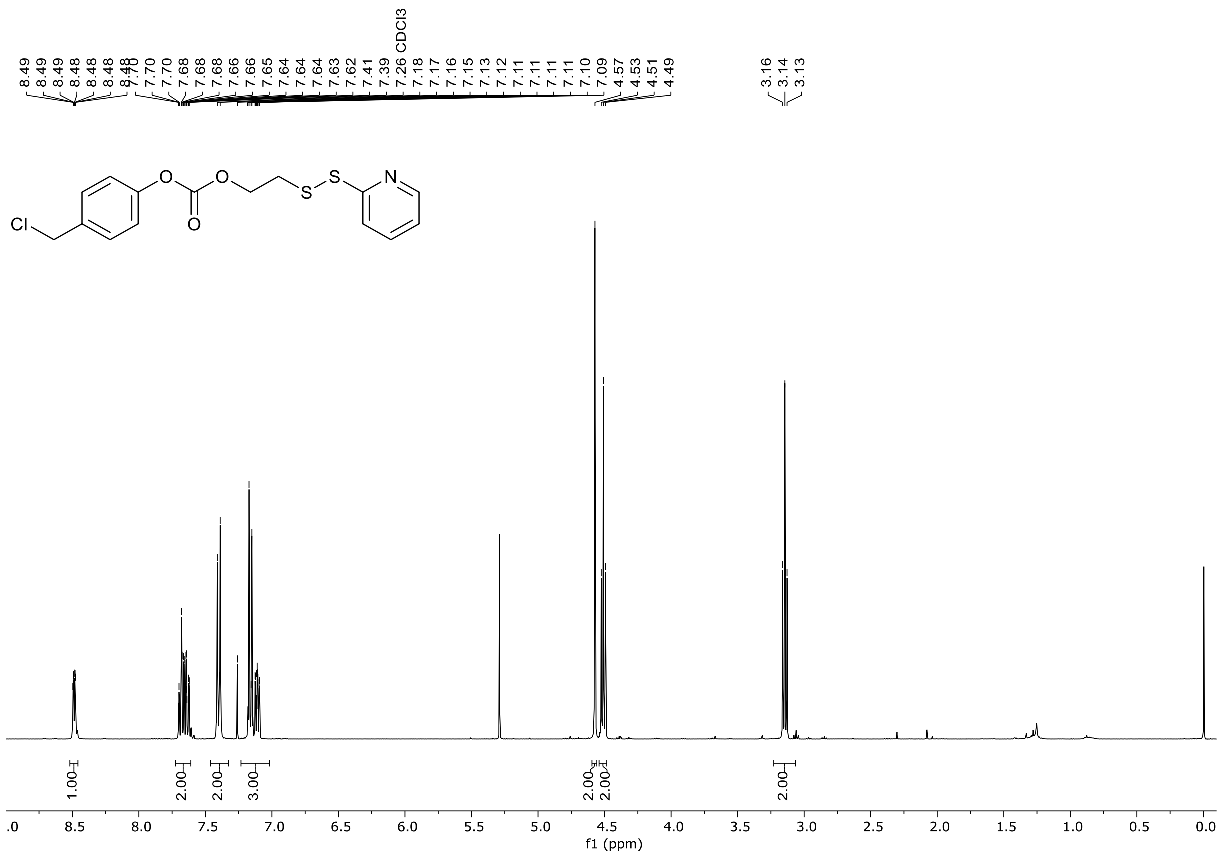

S-6: ^{13}C NMR ($CDCl_3$, 100 MHz)

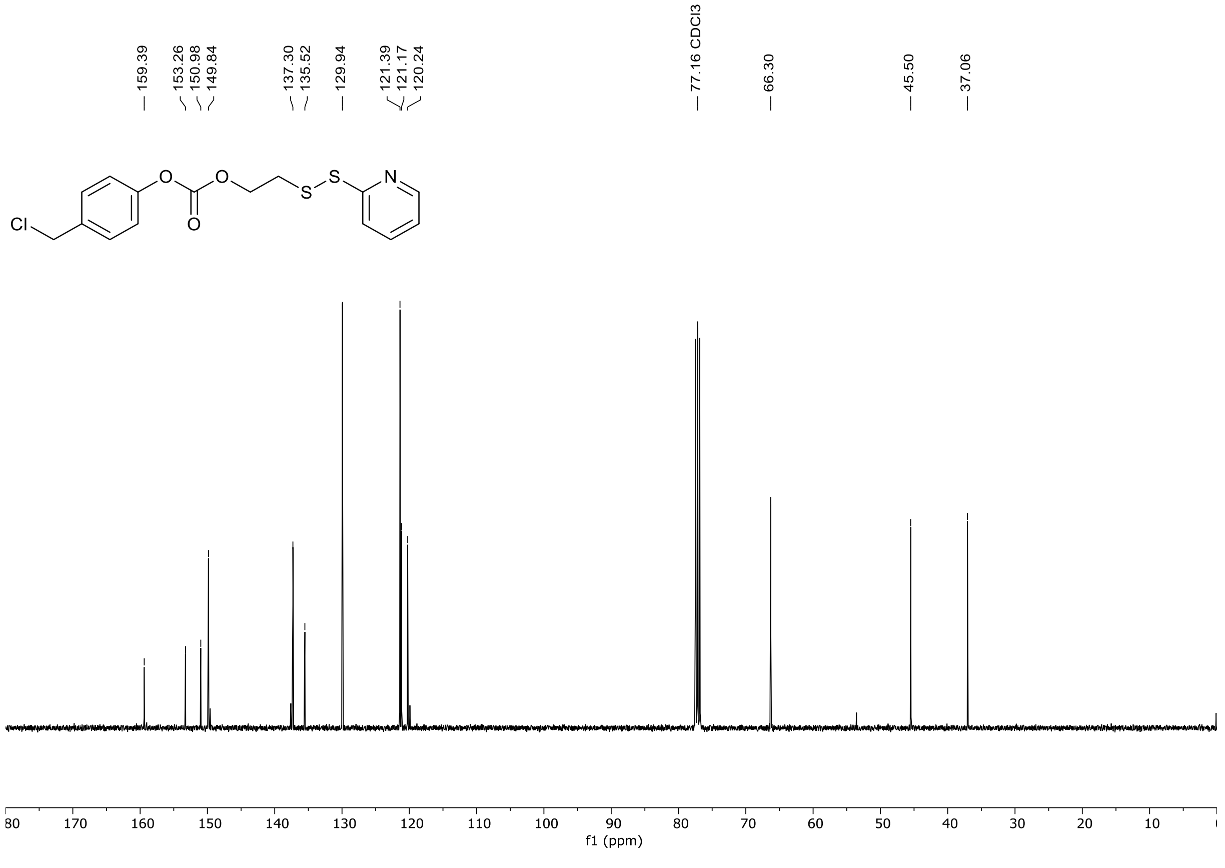

S-7: ^{1}H NMR ($CDCl_3$, 400 MHz)

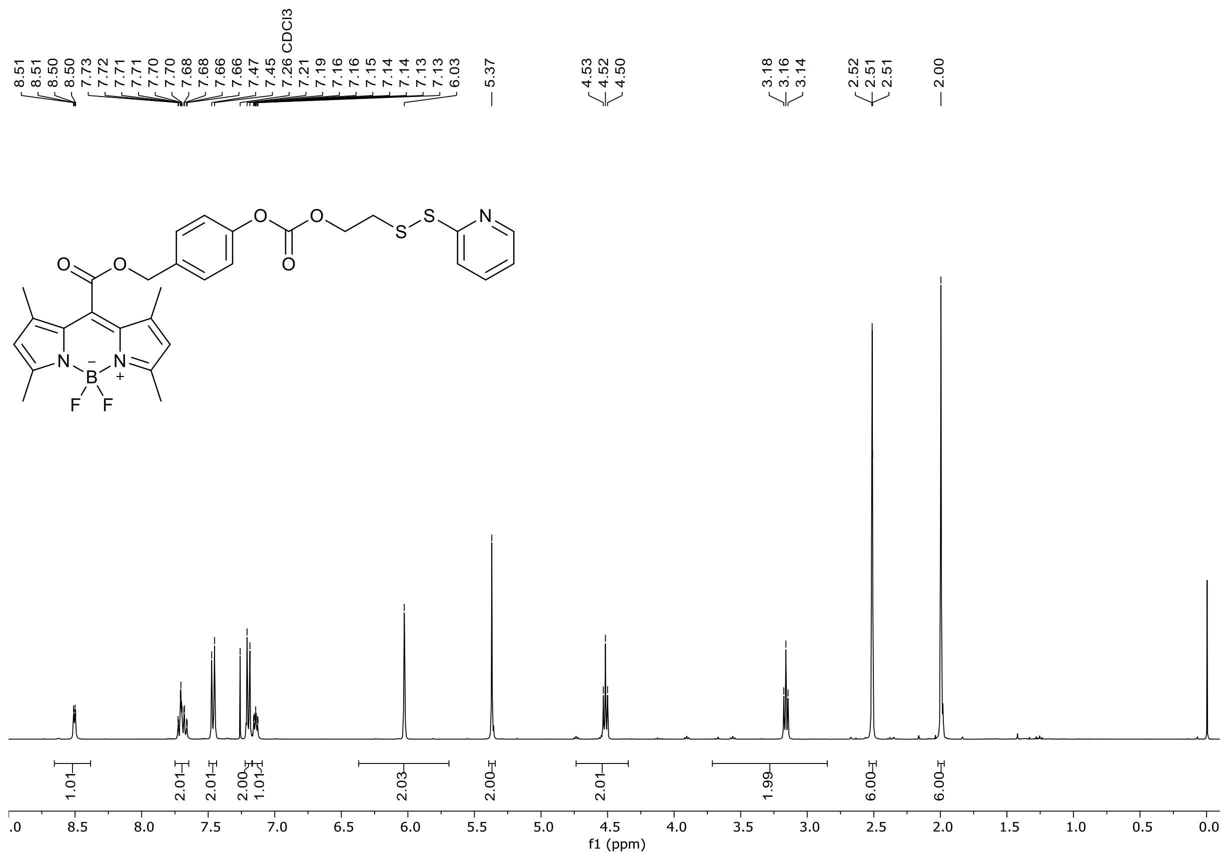

S-7: ^{13}C NMR ($CDCl_3$, 100 MHz)

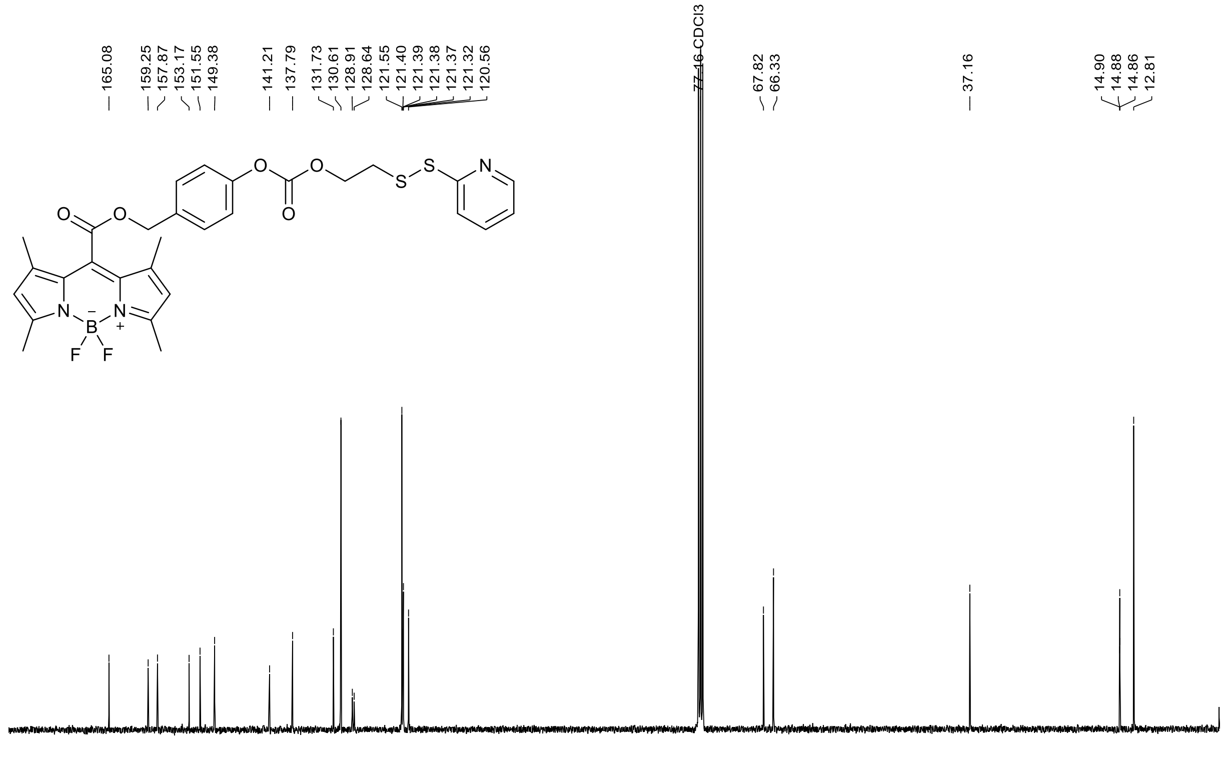

13: ^{1}H NMR ($CDCl_3$, 500 MHz)

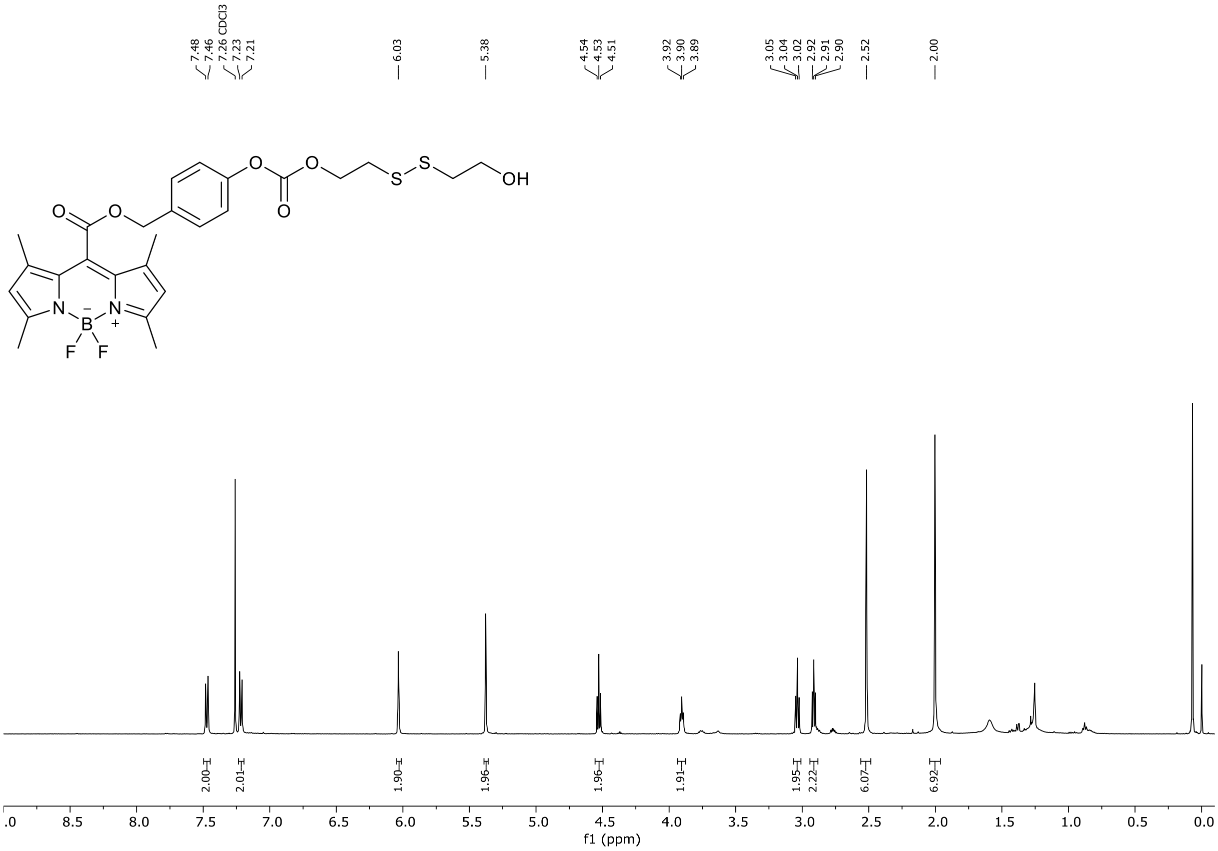

13: ^{13}C NMR ($CDCl_3$, 125 MHz)

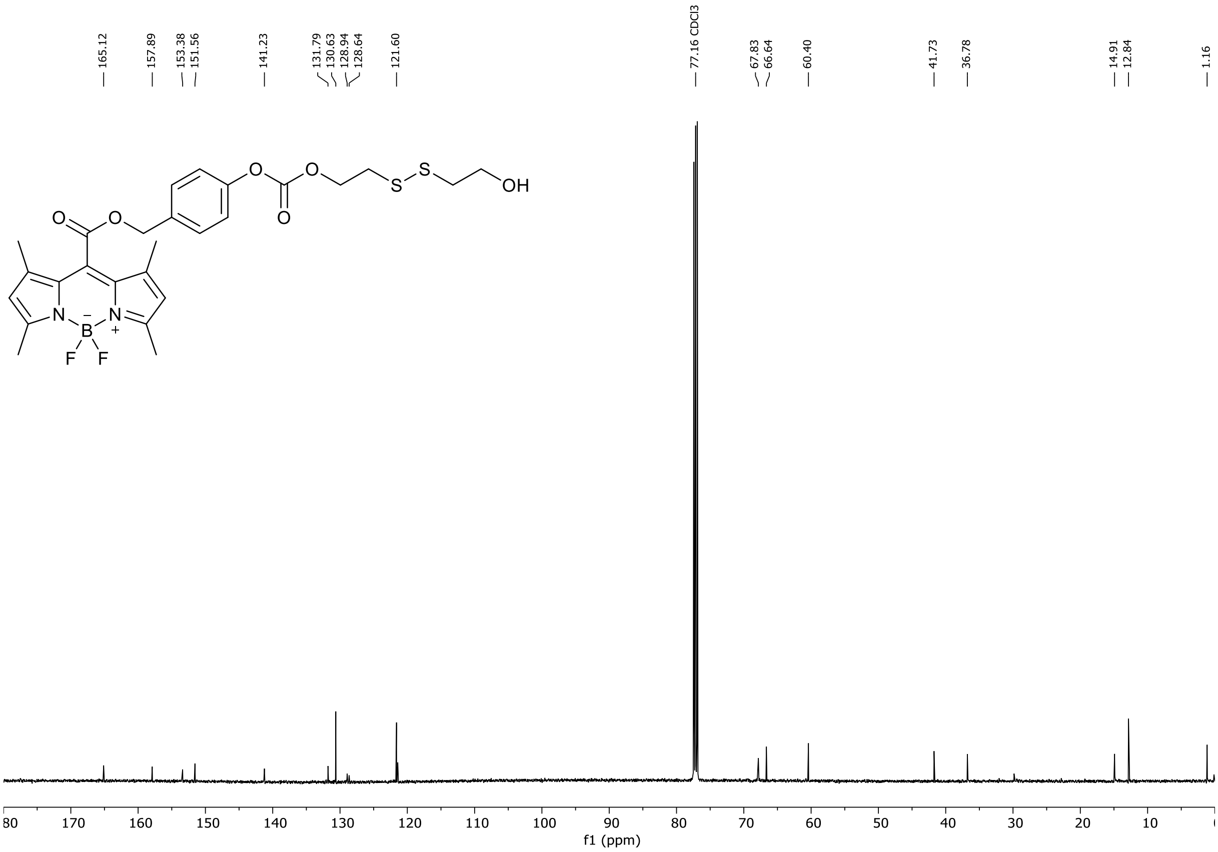

13: ^{1}H NMR ($CDCl_3$, 400 MHz)

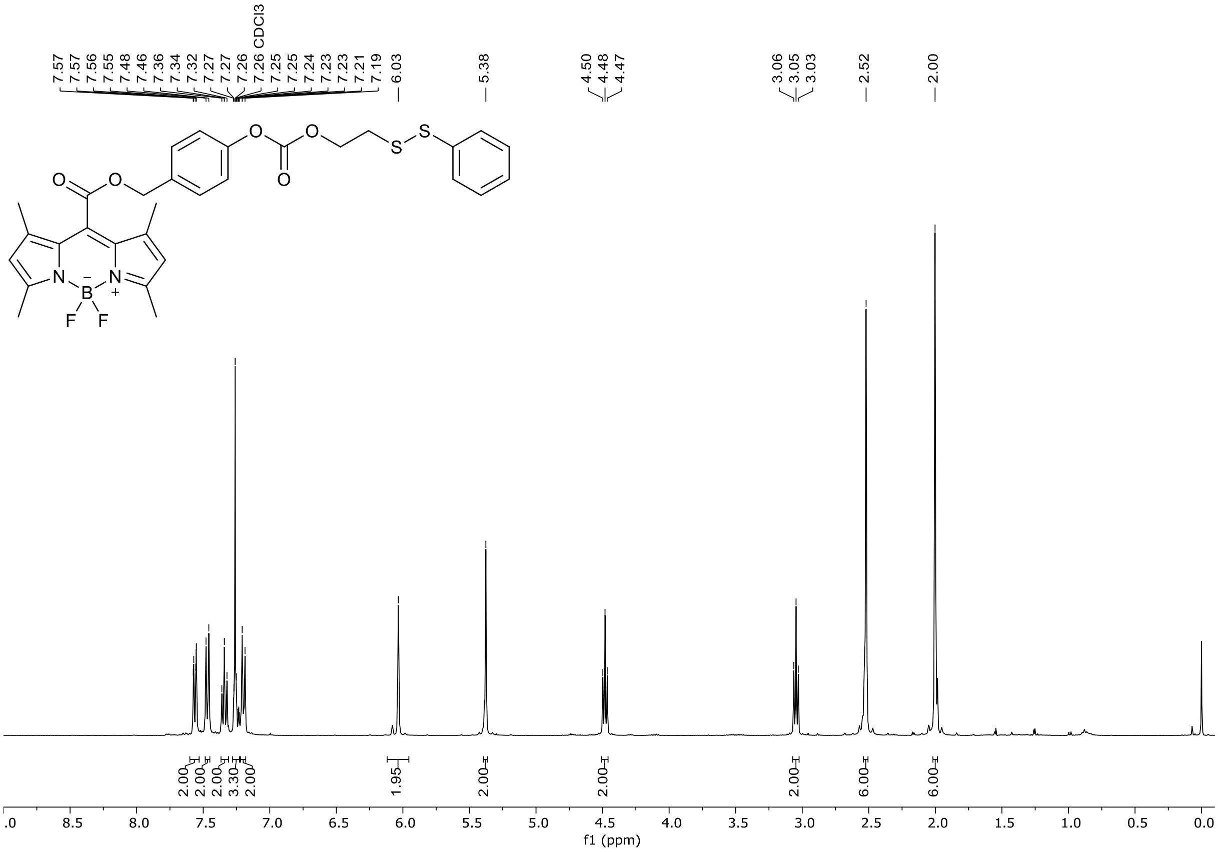

13: ^{13}C NMR ($CDCl_3$, 100 MHz)

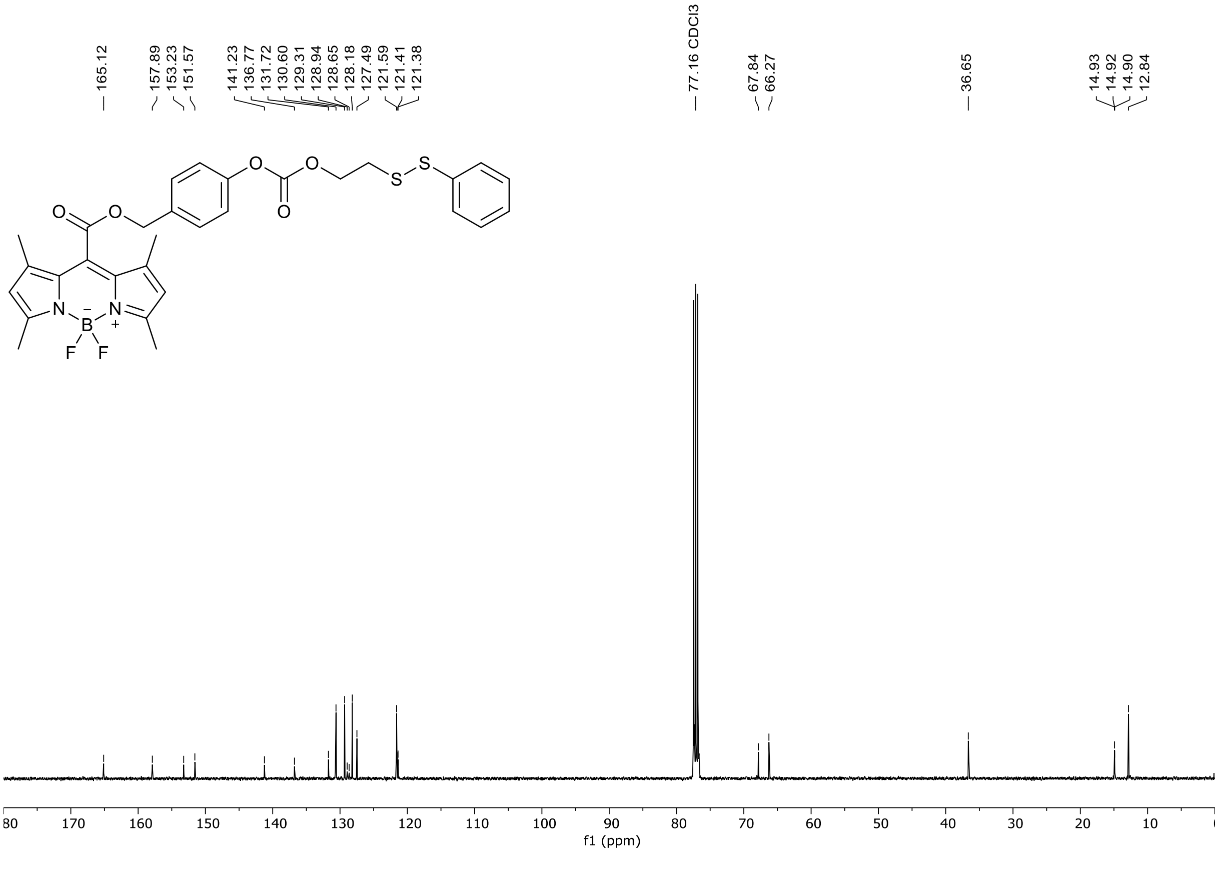

S-8: ^{1}H NMR ($CDCl_3$, 400 MHz)

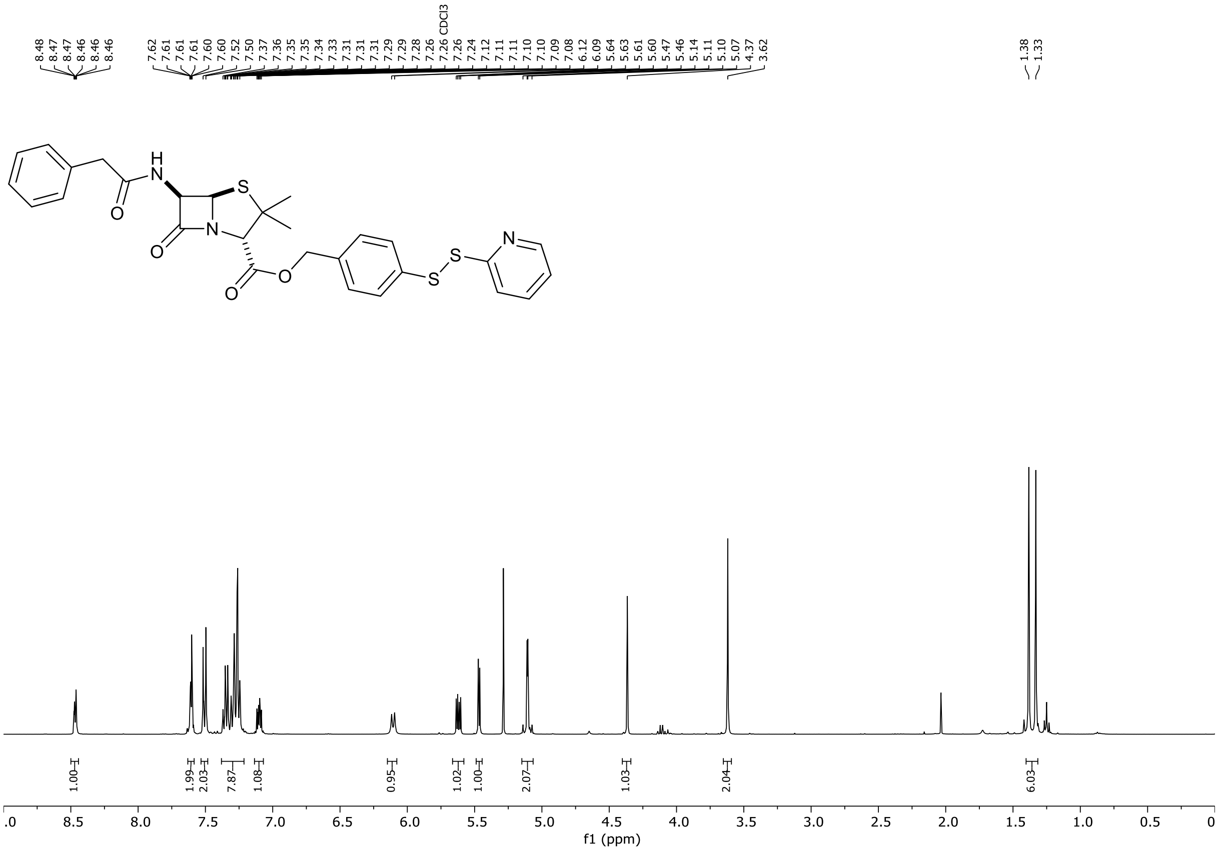

S-8: ^{13}C NMR ($CDCl_3$, 100 MHz)

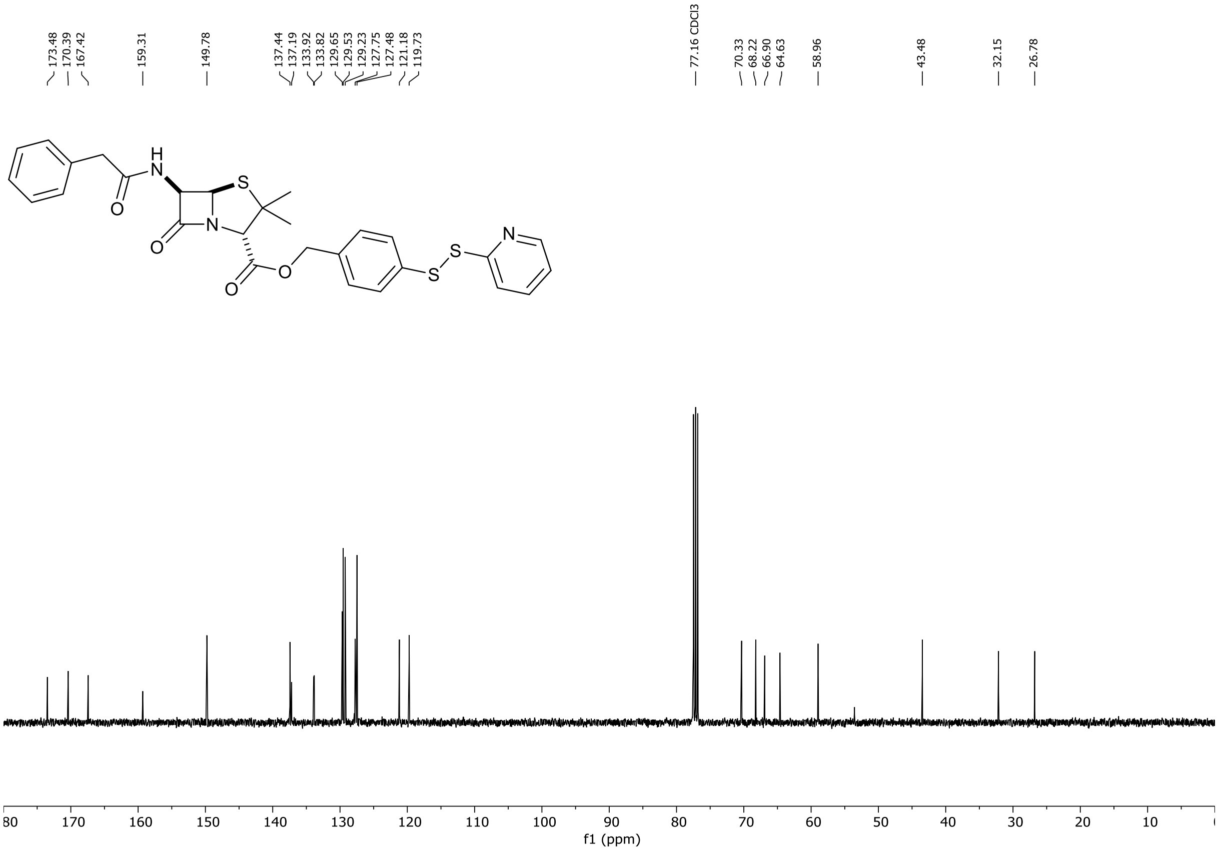

15b: ^{1}H NMR ($CDCl_3$, 400 MHz)

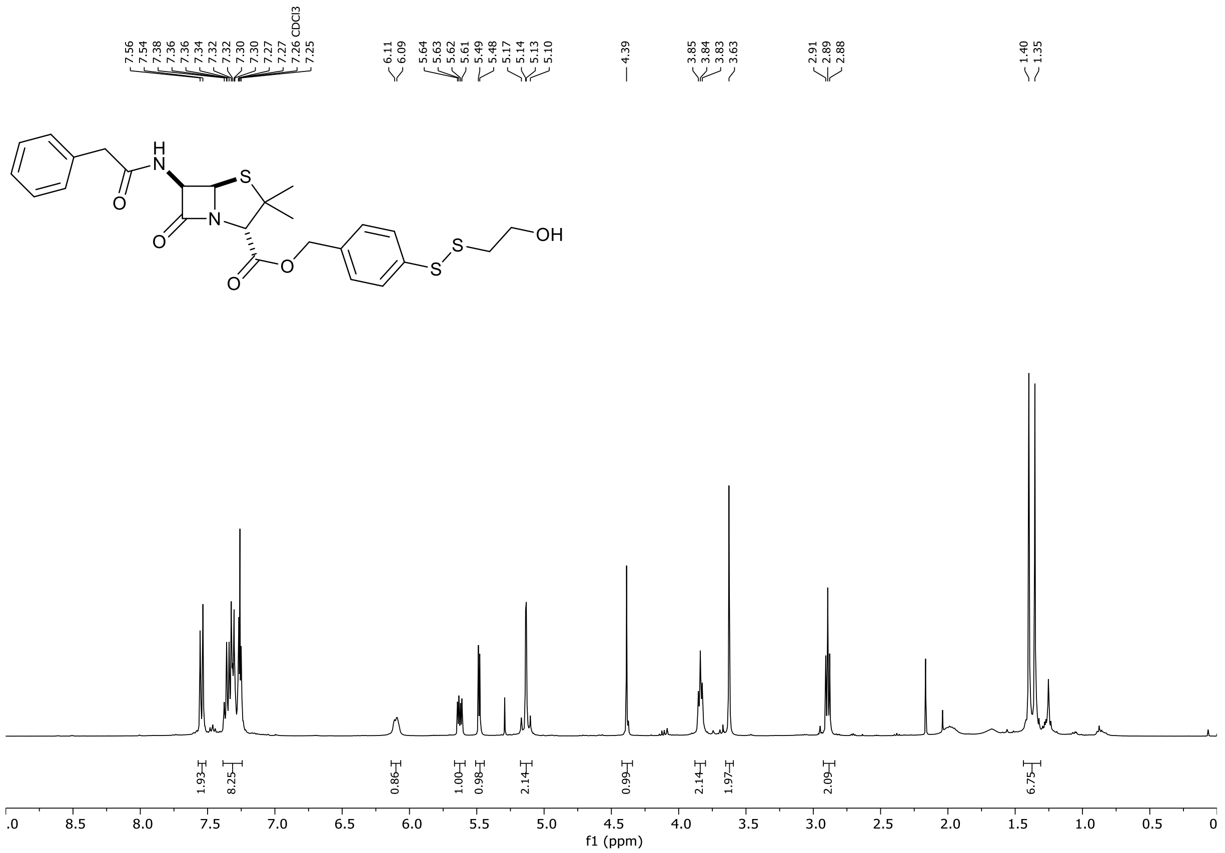

15b: ^{13}C NMR ($CDCl_3$, 100 MHz)

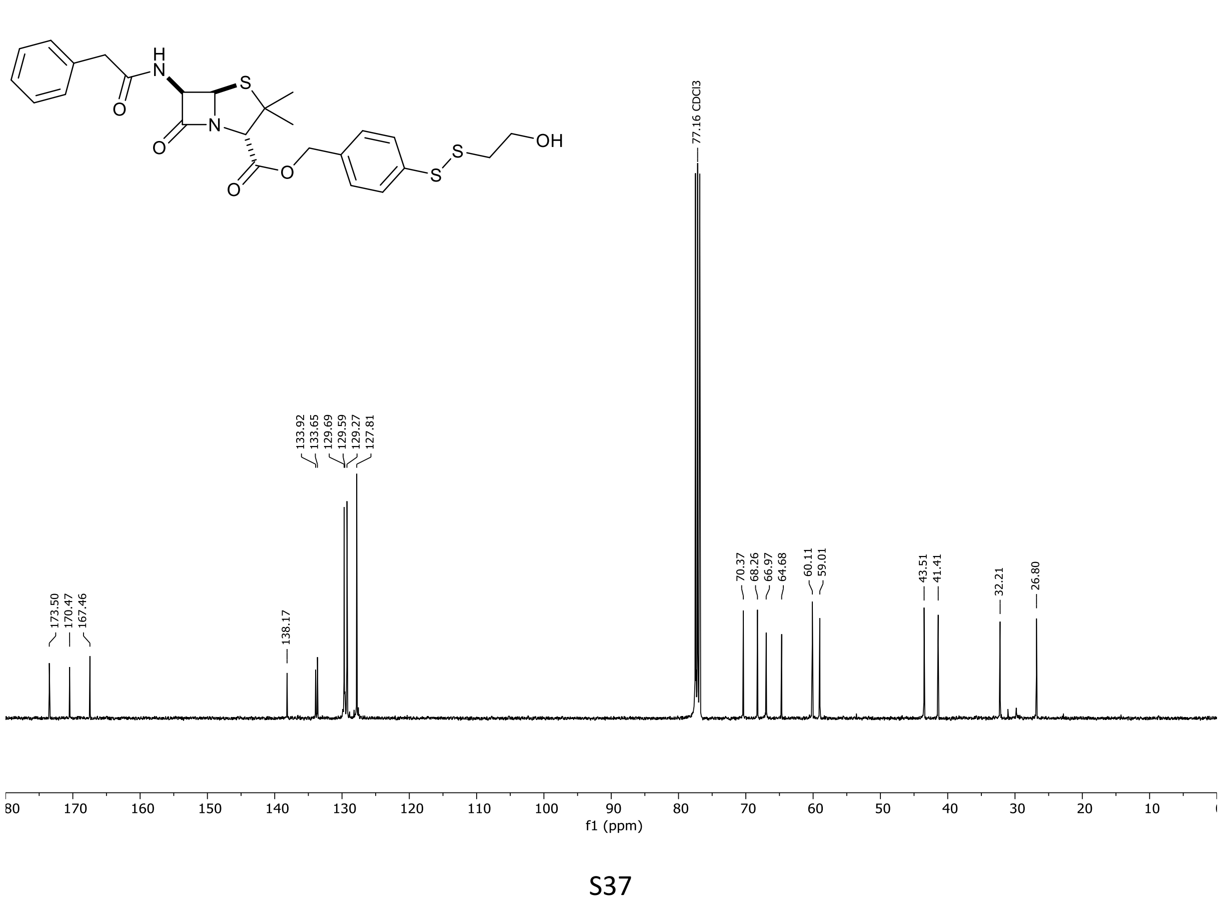

S-9: ^{1}H NMR ($CDCl_3$, 400 MHz)

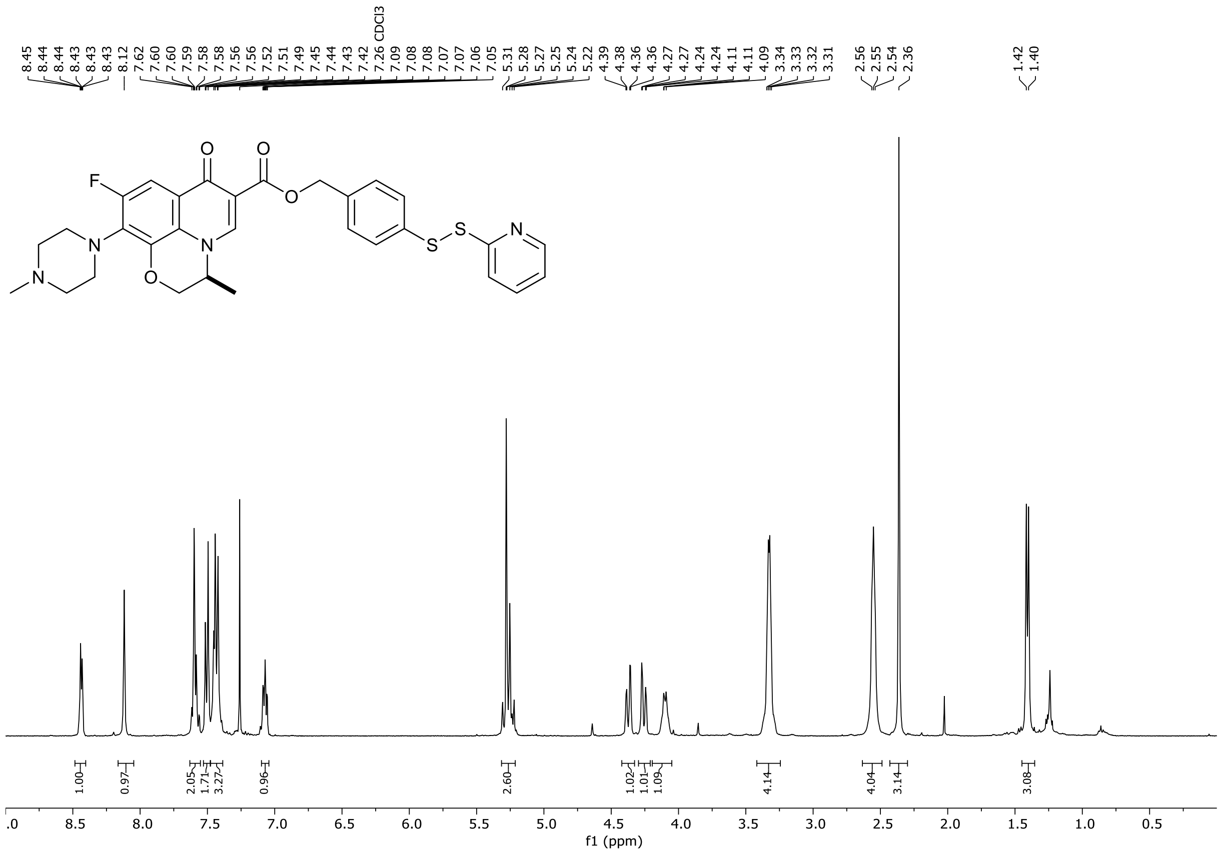

S-9: ^{13}C NMR ($CDCl_3$, 100 MHz)

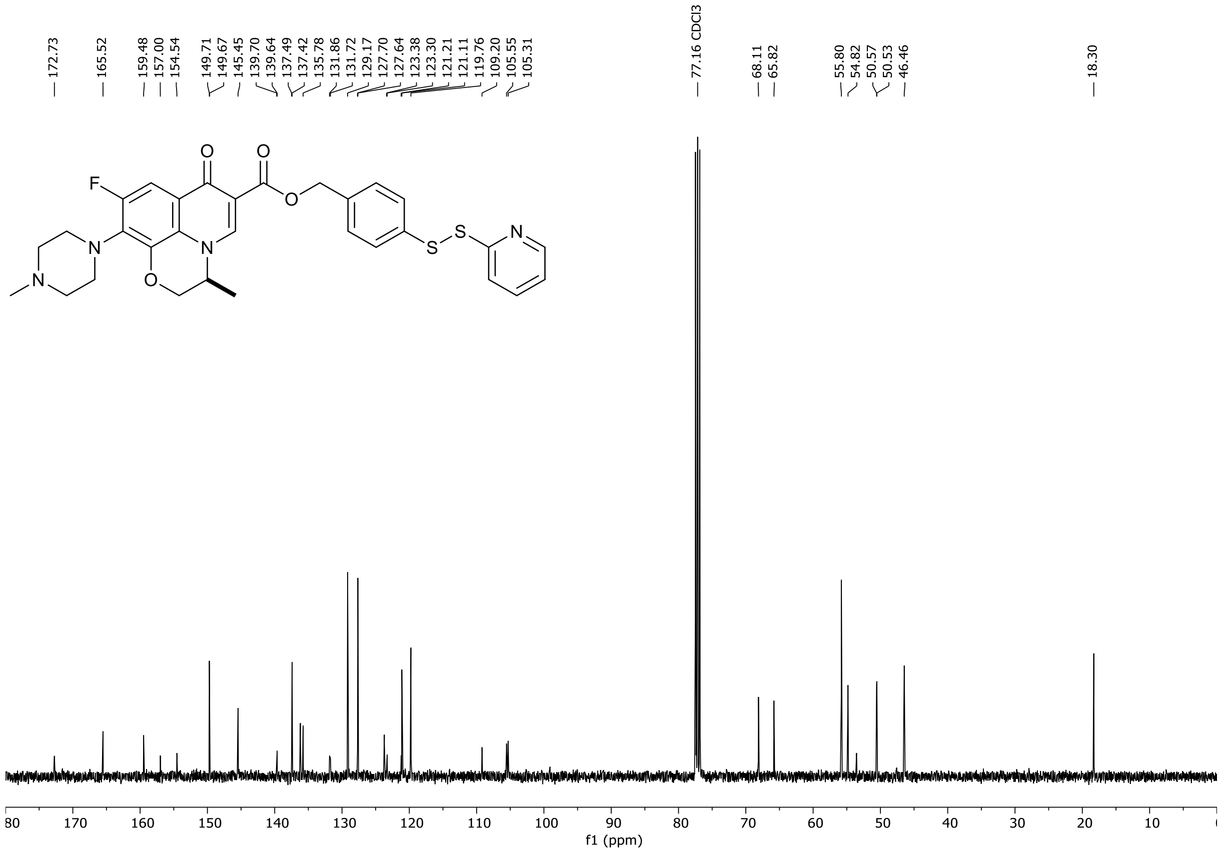

16b: ^{1}H NMR ($CDCl_3$, 400 MHz)

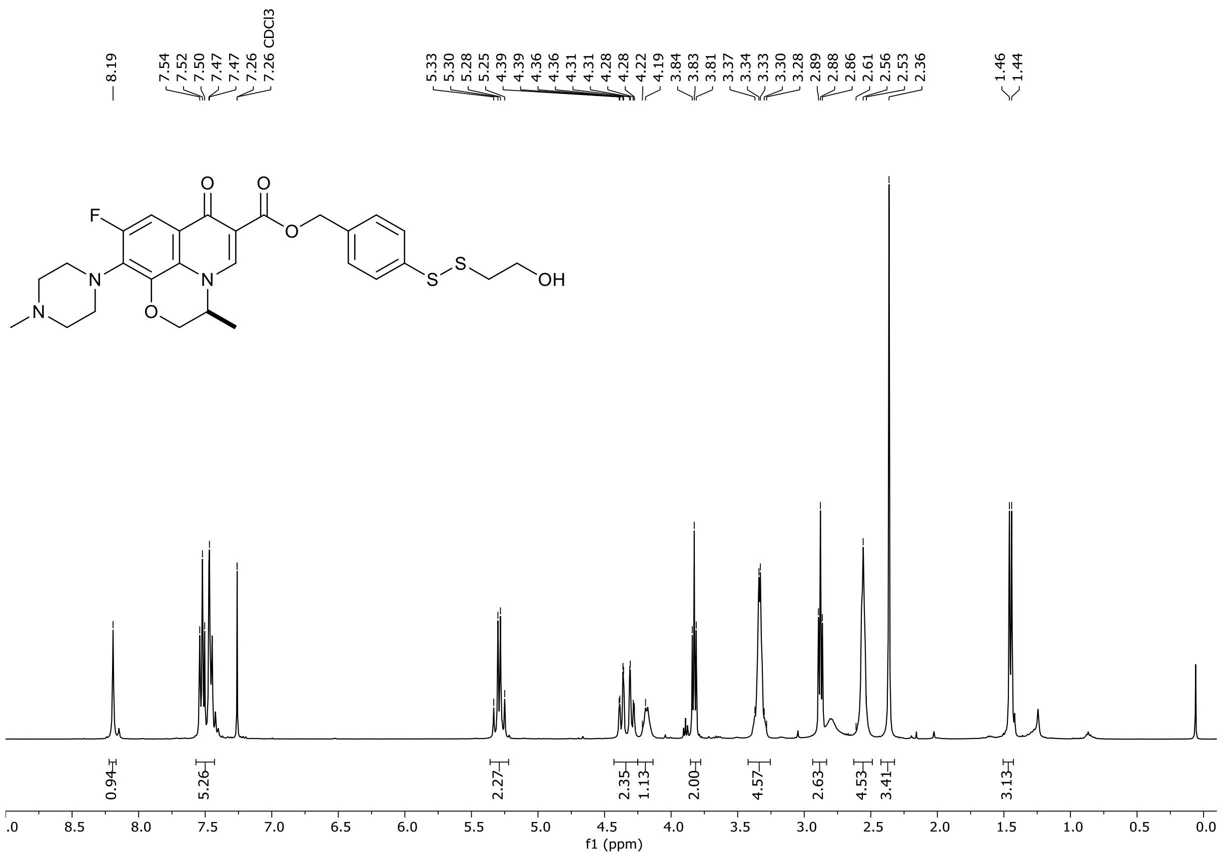

16b: ^{13}C NMR ($CDCl_3$, 100 MHz)

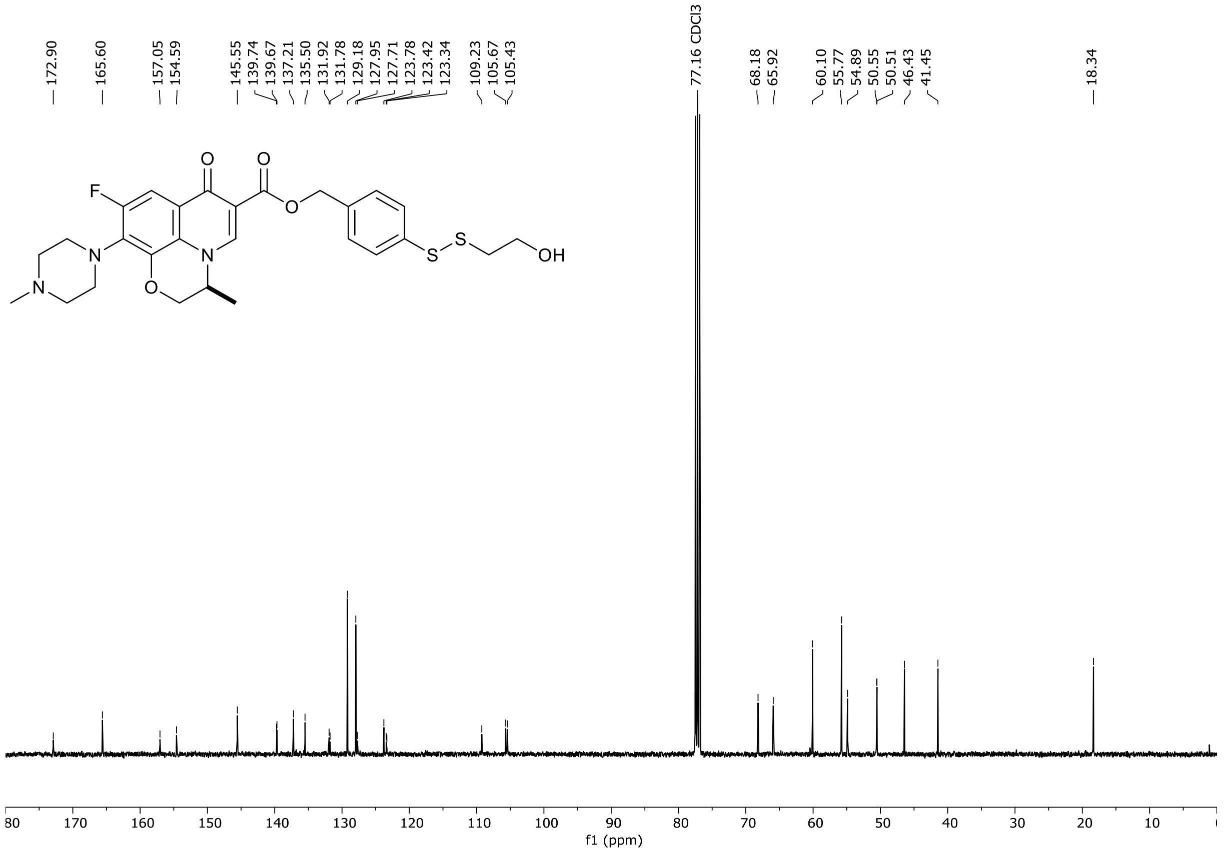

S-10: ^{1}H NMR ($CDCl_3$, 400 MHz)

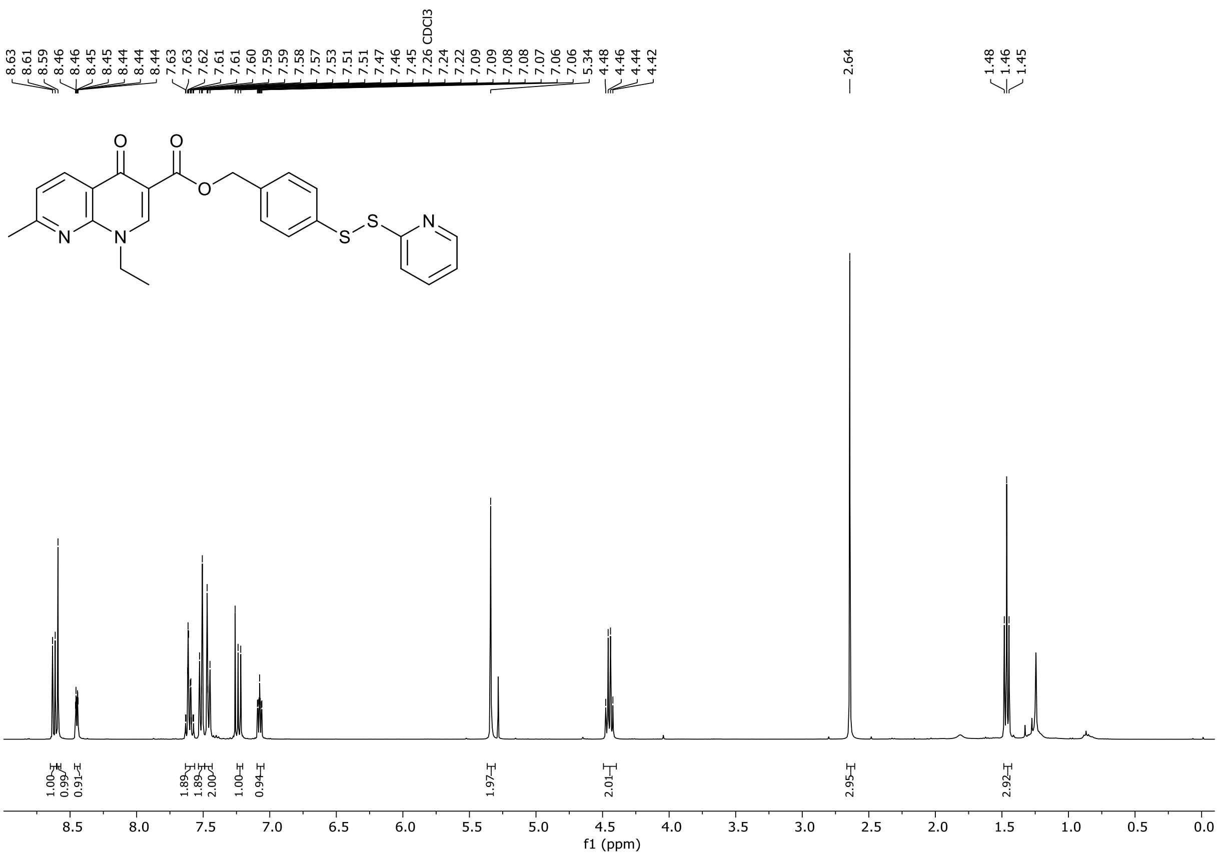

S-10: ^{13}C NMR ($CDCl_3$, 100 MHz)

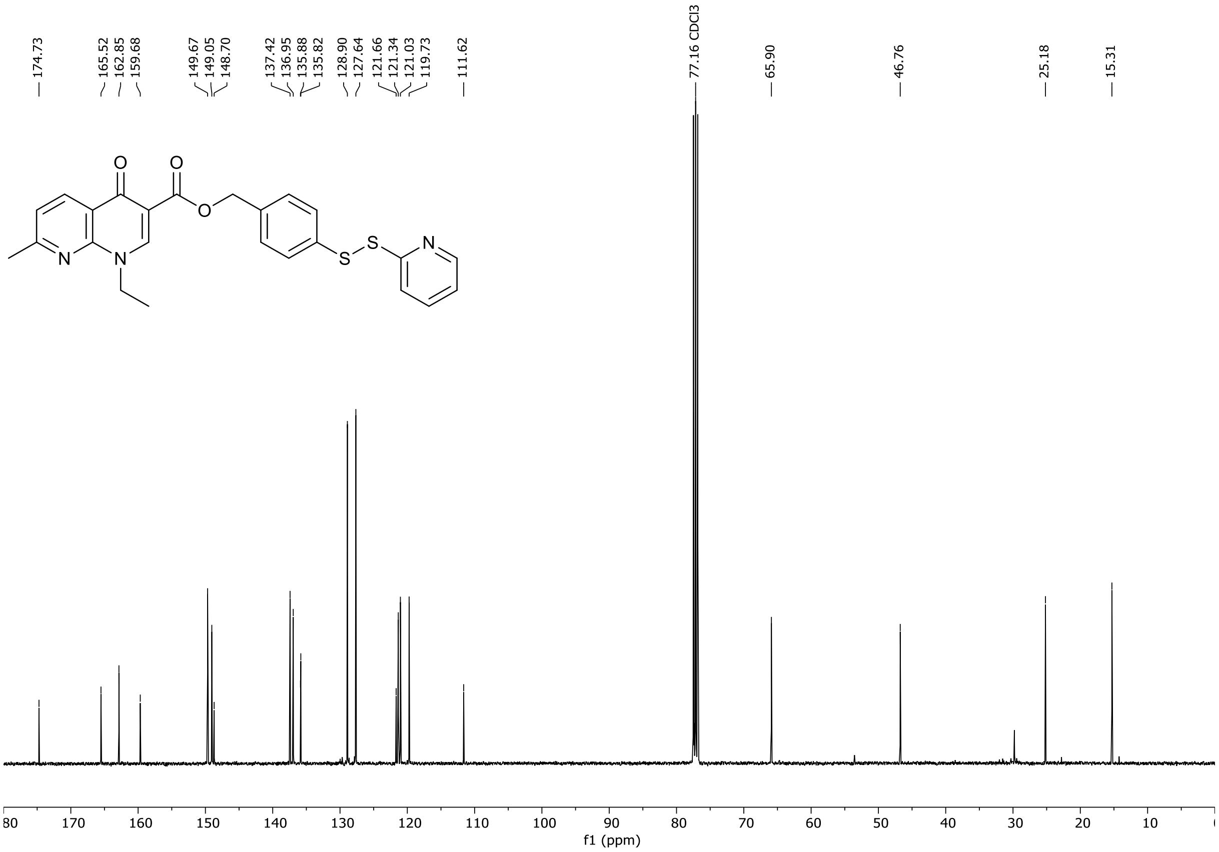

17b: ^{1}H NMR ($CDCl_3$, 400 MHz)

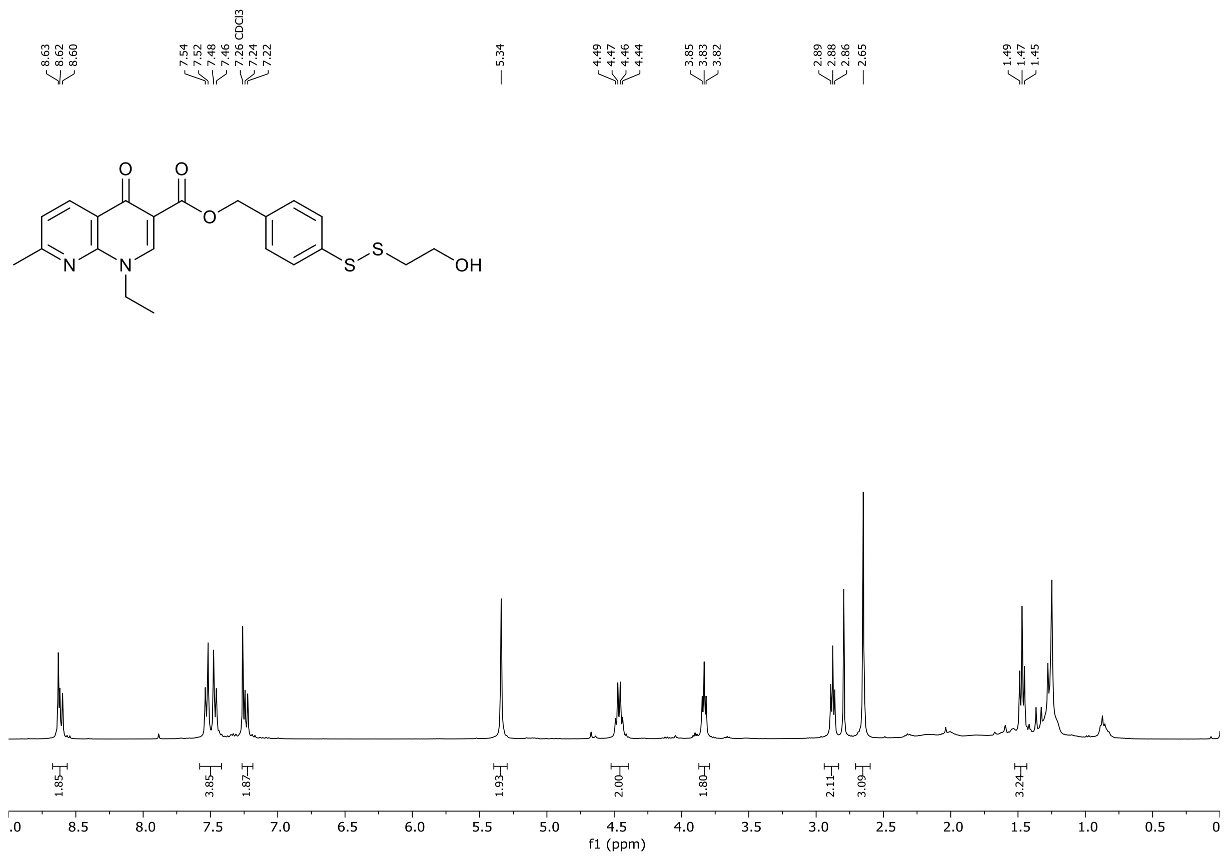

17b: ^{13}C NMR ($CDCl_3$, 100 MHz)

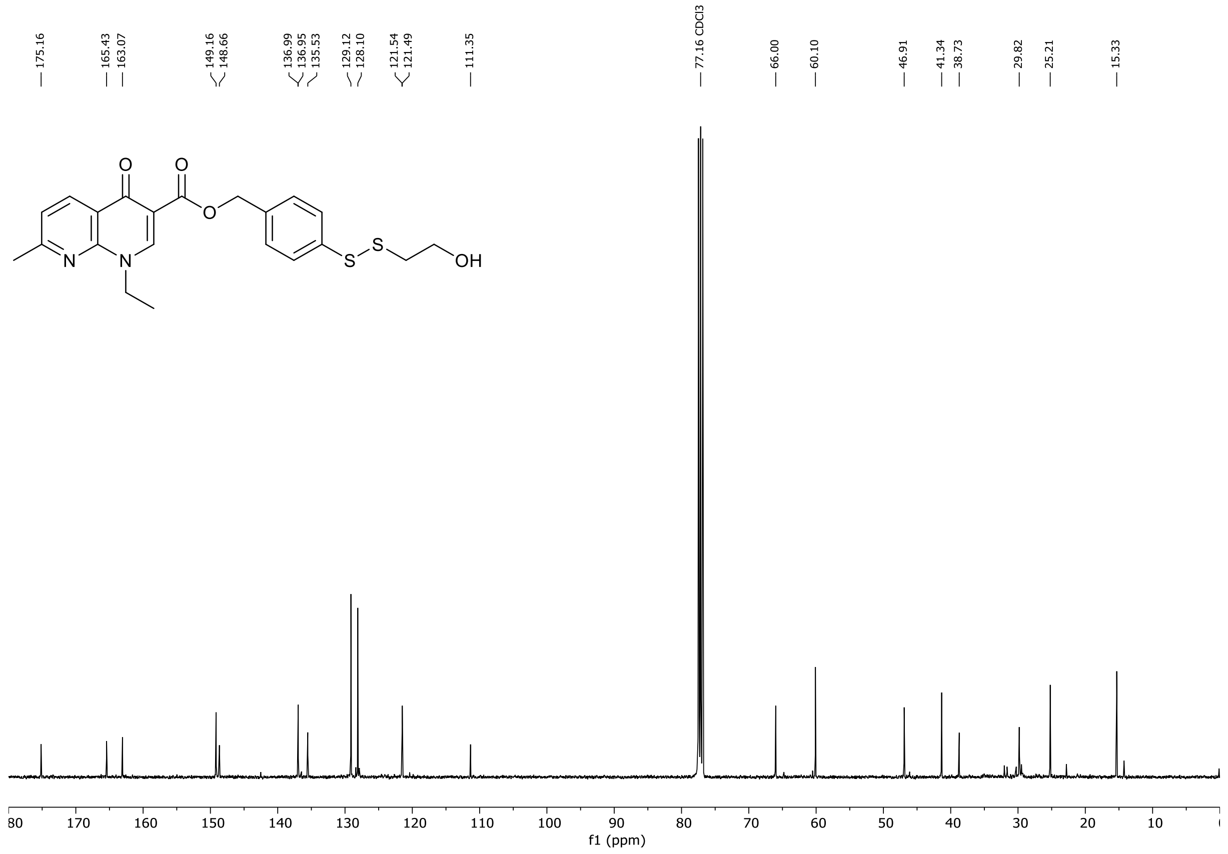

4. References

1) C. Rousset, M. Fontcave, S. Ollagnier de Choudens, *FEBS Lett.* **2008**, *582*, 2944.

2) A. Berwa, Y. Caspar, *J. Glob. Antimicrob. Resist.* **2024**, *39*, 153.

3) T. Sun, A. Morger, B. Castagner, J.-C. Leroux, *Chem. Commun*. **2015**, *51*, 5721.

4) G. Liu, Z. Jiang, J. F. Lovell, L. Zhang, Y. Zhang, *ACS Appl. Bio Mater*. **2021**, *4*, 4982.
